# Longitudinal trends in master track and field performance throughout the aging process: 83,209 results from Sweden in 16 athletics disciplines

**DOI:** 10.1007/s11357-020-00275-0

**Published:** 2020-10-13

**Authors:** Bergita Ganse, Anthony Kleerekoper, Matthias Knobe, Frank Hildebrand, Hans Degens

**Affiliations:** 1 Research Centre for Musculoskeletal Science & Sports Medicine, Faculty of Science and Engineering, Manchester Metropolitan University, John Dalton Building, Manchester, UK; 2 Department of Computing and Mathematics, Faculty of Science and Engineering, Manchester Metropolitan University, John Dalton Building, Manchester, UK; 3 Department of Orthopaedic and Trauma Surgery, Lucerne Cantonal Hospital, Lucerne, Switzerland; 4 Department of Orthopaedic Trauma Surgery, RWTH Aachen University, Aachen, Germany; 5 Institute of Sport Science and Innovations, Lithuanian Sports University, Kaunas, Lithuania

**Keywords:** Longevity, Lifespan, Big data, Athletics, Sports, Successful aging

## Abstract

In the research of age-related performance declines, the value of cross-sectional versus longitudinal data is an ongoing debate. This paper analyses the largest longitudinal master track and field data set ever published to compare the age-related decline in performance between 16 athletics disciplines in cross-sectional and longitudinal data. The data set contained 83,209 results (64,948 from men, 78.1%; 18,261 from women, 21.9%) from 34,132 athletes (26,186 men, 76.7%; 7946 women, 23.3%), aged 35–97 years. In 61 athletes, 20 or more, and in 312 athletes, 15 or more results were available. The data were analyzed by regression statistics/ANCOVA. Men had a higher performance than women, irrespective of discipline in both cross-sectional and longitudinal data (*p* < 0.001). The performance in cross-sectional data was lower compared with the longitudinal data in all events and at any age (*p* ≤ 0.007) except for 1000 m men. The average age was lower in the cross-sectional than the longitudinal data (*p* < 0.001); men 46 and 58 years, women 44 and 56 years, respectively. The annual percentage rate of decline did not differ significantly between cross-sectional and longitudinal data, or between sexes in most disciplines. Performance declines after age 70 were 1.7 times (men) and 1.4 times (women) as steep as before. In conclusion, although longitudinal master athletics data of athletes with 10 and more results has higher average performance and age compared with cross-sectional data, cross-sectional data give a good impression of the annual percentage decline in performance, which was similar in men and women.

**Electronic supplementary material:**

The online version of this article (10.1007/s11357-020-00275-0) contains supplementary material, which is available to authorized users.

## Introduction

Athletic performance declines with age, despite our best efforts to stop or minimize losses in physical capabilities by exercise, nutrition and further anti-aging interventions [1–3]. While much is known on performance declines in running with age [4–8], less is known on performance trajectories in other athletics disciplines, such as jumping and throwing [9].

In the research of effects of countermeasures, diseases and injuries on the rate of performance declines, it has often been argued that cross-sectional (CS) data may not adequately represent longitudinal (LN) changes [7, 10, 11]. For example, in a data set of Canadian Masters track rankings, the decline over a 5-year period was found to be approximately twice as steep when using CS data than the decline determined from LN data [7]. The discrepancy between CS and LN data to assess performance decline was also illustrated by a linear performance decline in running performance of 15 master runners over a 20-year period as derived from LN data and a curvilinear decline when using CS [8]. LN data sets with identifiable individual decline trajectories allow for a more accurate characterization of actual decline rates, taking into account inter-individual differences, and are therefore most desirable [7, 11, 12]. Yet, because of the larger availability of CS data, most studies on age-related performance declines in athletes are based on CS data [1, 6, 13–15], world records or results from international championships [9, 16–19]. Indeed, longitudinal data are not widely available and in particular LN data spanning more than one decade. It remains to be seen whether such discrepancies in the age-related trajectory of performance decline persist also in a large data set.

Master athletes are 35 years and older and compete in 5-year bands, often until well into their 80s or even 90s. The popularity of master athletics has massively risen over the last decades, and ever-increasing numbers of athletes leave their highly standardized results in the annual ranking lists. Some countries publish lists of annual best results for competing master athletes, but those are often hard to access, some only published as books or in PDF format. The Swedish Track and Field Association, however, publishes their master athletics annual rankings online and open to the public. This database comprises an unprecedented amount of individual LN data, by far exceeding that in all previous studies [8, 11, 20]. Therefore, we used this Swedish database with the most comprehensive retrospective LN athletics data set ever published to compare the age-related trajectories in performance decline derived from its CS and LN data for 16 disciplines, including sprinting, running, jumping and throwing. As athletes who perform better usually compete longer and do not stop after 1 year or one competition, we hypothesized that (1) CS data is associated with a lower average performance than LN data, (2) athletes with only one result in the data set have a lower average age compared with athletes with 10 and more years of results and (3) that the rate of age-related decline is steeper in CS than LN data.

## Materials and methods

The Institutional Review Board of the Faculty of Medicine of Rheinisch-Westfälische Technische Hochschule Aachen (reference number EK 300/17) has approved the study.

### Generation of data set

The database of ‘Swedish Veteran Athletics’ (http://www.friidrott.info/veteran/index.php) that is publicly available was scraped to extract the annual best results of all master athletes participating in the following athletic disciplines in the years 1901 to 2018: 100 m, 200 m, 400 m, 800 m, 1000 m, 1500 m, 3000 m, 5000 m, 10 km, high jump, long jump, triple jump, pole vault, discus throw, shot put and javelin throw. Scraping and data formatting were performed using the Python-scripts (scraper, parser and combiner/formatter) as shown in Online Resource 1. The output files contain the best result of each year for each athlete (anonymization by numbers) arranged by age for each discipline. The total athlete number shown is the total number of athlete appearances.

### Statistical analysis

All statistical tests were executed with IBM® SPSS® Statistics version 25. *t* tests were applied to compare the average age of the participants for each discipline in the CS and LN data set. For direct visual comparison of decline trajectories, second-order polynomial models were used as this represents the trajectory of the age-related decline in performance better than a linear model [8, 11, 20]. However, in Online Resource 2, the regression equations resulting in the highest *R*^2^ values are shown for each discipline for both CS and LN data. To allow for a comparison of the annual percentage decline in performance with previous studies, we also presented the slopes of the linear regressions. To compare the performance between athletes with only one result in the data set (CS) and those with 10 and more results (LN), analyses of covariance (ANCOVAs) were conducted with factor data set (CS or LN) and covariate age. An additional ANCOVA analysis was performed on the data normalized to the average result value at age 35 to assess differences in the decline functions between CS and LN data and between the sexes. Disciplines were only included in the analysis if the data set provided more than three athletes with 10 results and more. Further analyses for athletes with 15, 20 or 30 results and more in the data set were conducted if three or more athletes were available. Significance was assumed at *p* < 0.05. Sufficient data of the discipline 1500 m was only available for the women and not for the men, while 5000 m, triple jump and pole vault did not meet the criteria in the women. For this reason, only 15 disciplines are shown for the men and 12 for the women, even though 16 disciplines were considered and analyzed in total.

### Implement specifications

For the interpretation of performance declines, it is important to consider that changing implement weights cause a bias in the results of regression statistics. In the present study, this phenomenon affected the shot put, discus and javelin throw. Implement specifications are regulated by World Master Athletics (Appendix A-C Hurdles & Implements).^1^ For the women, implement weights are as follows: shot put: 35–49 years: 4 kg, 50–74 years: 3 kg, 75+ years: 2 kg; discus: 35–74 years: 1 kg, 75+ years 0.75 kg; javelin: 35–49 years: 600 g, 50–74 years: 500 g, 75+ years: 400 g. The men’s specifications are shot put: 35–49 years: 7.26 kg, 50–59 years: 6 kg, 60–69 years: 5 kg, 70–79 years: 4 kg, 80+ years: 3 kg; discus: 35–49 years: 2 kg, 50–59 years: 1.5 kg, 60+ years: 1 kg; javelin: 35–49 years: 800 g, 50–59 years: 700 g, 60–69 years: 600 g, 70–79 years: 500 g, 80+ years: 400 g. The women’s javelin specification changed in April 1999, following a design change of the men’s javelin in April 1986. As several athletes had thrown so far that the grass field in standard stadiums was not long enough anymore, it was necessary to reduce the distance a javelin would fly by shifting the balance point forward to make the javelin drop earlier [21].

As changes in implements may decrease the apparent rate of the age-related decline in performance, we have analyzed the declines of the throwing disciplines separate from the sprint, runs and jumps.

## Results

A total of 83,209 results (64,948 from men, 78.1%; 18,261 from women, 21.9%) from 34,132 athletes (26,186 men, 76.7%; 7946 women, 23.3%) with an age between 35 and 97 years were analyzed. Table 1 shows the distribution of the athletes over the different disciplines and for how many athletes we had a single data point for a given discipline, or longitudinal data. Online Resource 2 shows the individual performance trajectories for all disciplines with regression lines and equations for those athletes with only one data point, or 10, 15, 20 and 30 or more LN results whenever three or more athletes were available.

**Table 1:** Athlete and result numbers in the data set

	Men						Women					
	Number of athletes with				Total no of athletes	Total no of results	Number of athletes with				Total no of athletes	Total no of results
	≥ 10 results	≥ 15 results	≥ 20 results	Only one result			≥ 10 results	≥ 15 results	≥ 20 results	Only one result		
100 m	48	12	2	778	1355	3201	19	9	4	311	520	1255
200 m	39	6	0	661	1196	2884	15	8	3	179	365	999
400 m	33	4	0	665	1234	2925	14	8	4	207	358	915
800 m	44	3	0	925	1708	4044	19	9	4	371	598	1385
1000 m	4	1	0	570	798	1267	n/a	n/a	n/a	n/a	n/a	n/a
1500 m	n/a	n/a	n/a	n/a	n/a	n/a	17	9	3	417	686	1534
3000 m	9	1	0	1142	1734	2988	4	0	0	394	609	1045
5000 m	73	13	0	1783	3282	7545	n/a	n/a	n/a	n/a	n/a	n/a
10 k	61	7	0	1728	3278	7462	10	3	0	402	699	1431
High jump	40	15	3	557	1088	3052	8	3	1	207	335	722
Long jump	34	5	0	605	1074	2579	11	4	1	249	397	846
Triple jump	16	4	0	287	527	1287	3	1	0	68	102	210
Pole vault	30	10	3	318	629	1786	1	0	0	24	42	97
Discus	198	49	14	1368	2819	8692	44	16	4	590	1033	2643
Shot put	159	50	7	1562	2988	8436	37	15	5	780	1333	3147
Javelin	130	43	1	1302	2476	6800	27	4	2	519	869	2032
Sum	918	223	30	14,251	26,186	64,948	229	89	31	4718	7946	18,261

### Comparison of disciplines

**Fig. 1:**
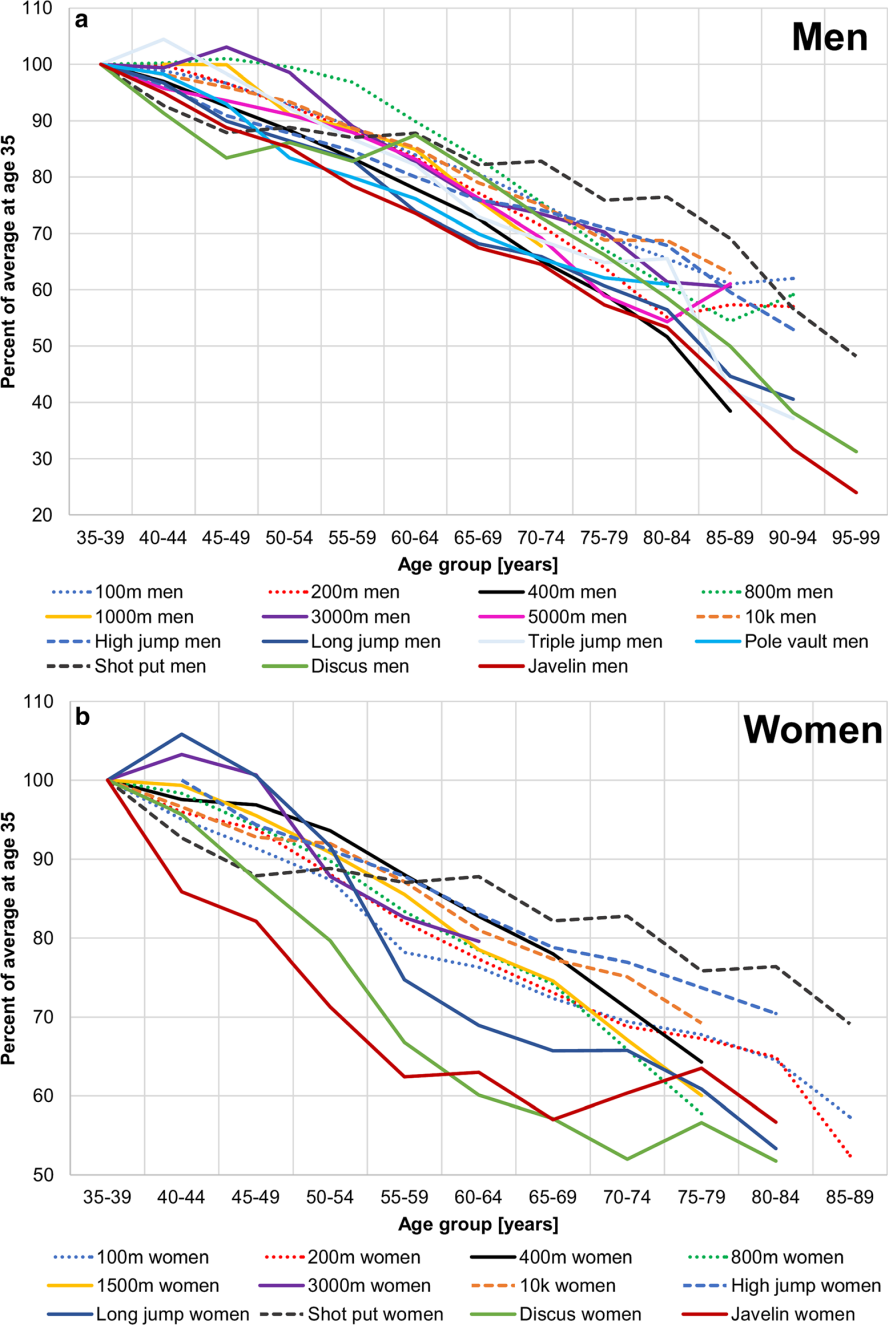
Comparison of performance declines in the analyzed athletics disciplines in percent normalized to age 35. The 5-year steps are pooled from all athletes with 10 and more results in the data set. Disciplines are only shown when data of more than 3 athletes with 10 results and more exists. 100 m men and high jump women were normalized to the average at age 40 due to a lack of data in group 35–39. **a** Men, **b** women. Note the particularly steep declines of the women’s discus and javelin throw before the age of 60 years

The oldest female athletes in the data set were under 90 years, while the oldest male athletes were in their mid-90s. Figure 1 shows the percent performance declines with age for each sex in 5-year groups for all disciplines normalized to the average performance at age 35 in athletes with 10 or more data points. The figure suggests that the women’s discus and javelin declined the steepest before age 60 years (declines differed significantly between men and women; for javelin and discus both *p* < 0.001). The figure also suggests that in the men’s triple jump, 800 m and 3000 m, and in the women’s long jump and 3000 m, an initial increase in performance occurred before the age of 50 years. Table 2 shows the rates of performance declines in %/year for each discipline.

**Table 2:** Performance decline rates in %/year normalized to age 35. It should be noted that the polynomial regression fit the data better than linear data, as shown in Online Resources 2 and 3. Performance decline rates are shown for before and after 70 years, as the rate of performance decline is known to accelerate around age 70 after staying relatively constant before that age [1, 2, 17]

	All athletes with 10 results or more in the data set								Only one result in data set			
	All athletes (35 until oldest)		35–69 years		70 years until oldest		Increase in older group divided by younger group		All athletes (35 until oldest)		Slope CS/slope LN	
	Men	Women	Men	Women	Men	Women	Men	Women	Men	Women	Men	Women
100 m	0.66	0.84	0.65	0.71	0.72	1.12	1.11	1.58	0.91	0.66	1.38	0.78
200 m	0.82	1.03	0.75	0.78	0.94	1.54	1.25	1.97	0.97	0.98	1.19	0.95
400 m	1.36	0.92	0.86	0.67	2.36	1.81	2.75	2.72	1.02	0.73	0.75	0.80
800 m	0.79	0.99	0.71	0.96	0.98	1.28	1.37	1.34	1.01	0.85	1.29	0.86
1000 m	0.85^a^	n/a	0.88^a^	n/a	n/a	n/a	n/a	n/a	0.94	n/a	n/a	n/a
1500 m	n/a	0.82	n/a	0.71	n/a	1.02	n/a	1.43	n/a	0.87	n/a	1.06
3000 m	0.78	0.63	0.73	0.63	0.99	n/a	1.36	n/a	1.02	0.66	1.30	1.05
5000 m	0.82	n/a	0.84	n/a	0.72	n/a	0.86	n/a	0.84	n/a	1.02	n/a
10 k	0.75	0.84	0.69	0.84	0.95	1.56	1.37	1.85	0.77	0.63	1.03	0.74
High jump	0.88	0.73	0.88	0.42	1.12	1.53	1.27	3.62	0.91	0.84	1.03	1.14
Long jump	1.14	1.13	1.07	1.18	1.57	1.75	1.46	1.48	1.25	1.28	1.10	1.13
Triple jump	1.11	n/a	0.75	n/a	3.58	n/a	4.76	n/a	1.20	n/a	1.08	n/a
Pole vault	0.98	n/a	1.01	n/a	0.98	n/a	0.97	n/a	1.08	n/a	1.11	n/a
Discus	1.13	1.27	0.79	1.37	1.59	1.15	2.03	0.84	1.09	1.07	0.97	0.84
Shot put	0.95	0.95	0.73	1.19	1.48	0.44	2.02	0.37	0.99	0.91	1.05	0.97
Javelin	1.29	1.29	1.10	1.44	1.67	1.19	1.52	0.82	1.42	1.62	1.10	1.26

^a^Normalized to age 40 due to lack of earlier data. These slopes of linear regressions are shown to allow for a comparison of the data to the literature

### Performance trajectories

The supplementary Figs. 1 and 2 in online resource 3 show second-order polynomial regression lines of the athletes with only one result in the data set (CS data, data points shown) to athletes with 10, 15, 20 and 30 results in the data set (LN data). It can be seen that the CS data is on average associated with a worse performance than the LN data at any age.

The statistical analysis by ANCOVA performed on the non-normalized data delivered the following results. The age effect, indicating an age-related decrease in performance, was highly significant (*p* < 0.001) in all disciplines and for both sexes. Sex differences were found in all disciplines where data from both sexes were available (100 m *p* < 0.001; 200 m *p* = 0.002; 400 m *p* < 0.001; 800 m *p* < 0.001; 3000 m *p* < 0.001; 10 km *p* = 0.001; high and long jump each *p* < 0.001; discus and shot put both *p* = 0.033; javelin throw *p* < 0.033). The performance in the CS data was less than that in the LN data at any age (with *p* < 0.001) in 100 m, 200 m, 400 m, 800 m, 1500 m, 3000 m, 5000 m, 10 km, high jump, long jump, discus throw, shot put, javelin throw, triple jump (*p* = 0.007) and pole vault (*p* = 0.002). In 1000 m (men only), there was no significant difference between CS and LN data (*p* = 0.153).

Table 3 shows differences between CS and LN data at age 50 as an example. Here, the average performance in the CS data only amounted to 86% (men) and 78% (women) of the LN data (10 results and more in the data set).

**Table 3:** Differences between CS and LN data of average results at age 50. Speed in m/s is shown for the sprinting and running, and metres for the jumping and throwing disciplines

	CS data from athletes with only one result in data set		LN 10 and more results		LN 15 and more results		LN 20 and more results		CS in percent of LN 10	
	Men	Women	Men	Women	Men	Women	Men	Women	Men	Women
100 m	7.09	5.87	7.80	6.76	7.69	6.80	n/a	6.64	90.92	86.77
200 m	6.79	5.89	7.55	6.58	7.61	6.48	n/a	6.33	89.90	89.57
400 m	6.00	5.12	6.67	5.86	6.65	5.79	n/a	5.79	89.96	87.41
800 m	5.35	4.11	5.49	5.06	5.72	5.47	n/a	5.50	97.41	81.21
1000 m	4.78	n/a	5.50	n/a	n/a	n/a	n/a	n/a	86.91	n/a
1500 m	n/a	4.07	n/a	4.55	n/a	n/a	n/a	n/a	n/a	89.60
3000 m	4.35	3.73	5.08	n/a	n/a	n/a	n/a	n/a	85.61	n/a
5000 m	4.24	n/a	4.68	n/a	5.01	n/a	n/a	n/a	90.67	n/a
10 k	4.17	3.42	4.53	4.21	4.90	3.76	n/a	n/a	92.05	81.34
High jump	1.42	1.09	1.52	1.31	1.54	n/a	1.50	n/a	93.01	83.21
Long jump	4.45	3.04	5.21	4.66	4.99	n/a	n/a	n/a	85.44	65.13
Triple jump	9.53	n/a	10.70	n/a	11.38	n/a	n/a	n/a	89.01	n/a
Pole vault	2.29	n/a	3.23	n/a	3.53	n/a	3.53	n/a	71.08	n/a
Discus	23.96	15.01	34.32	25.42	36.92	26.15	40.29	29.04	69.81	59.06
Shot put	8.81	6.74	10.74	8.90	11.48	8.99	11.35	9.23	82.03	75.70
Javelin	28.25	13.48	37.13	21.24	34.16	24.36	n/a	n/a	76.09	63.44
**Average**									**85.99**	**78.40**

CS data had a significantly lower average age compared with LN data: the average ages of the men were 46 years (CS) and 58 years (LN 10 years and more), while the women yielded 44 years (CS) and 56 years (LN 10 years and more) (*t* test, all *p* < 0.001, Table 4).

**Table 4:** Average ages. Comparison of the data of the athletes with only one result in the data set (CS) to those with 10 and more, 15 and more and 20 and more results (LN). The *t* test showed highly significant differences (*p* < 0.001) between the CS and LN (10 and more) data in all disciplines and both sexes

	Men				Women			
	10 and more	15 and more	20 and more	Only one	10 and more	15 and more	20 and more	Only one
100 m	58.60	63.88	n/a	48.28	60.58	64.22	63.84	44.90
200 m	58.90	63.56	n/a	48.53	60.65	61.63	63.39	44.97
400 m	58.94	61.72	n/a	47.63	57.27	57.32	59.07	45.03
800 m	58.46	64.02	n/a	46.70	55.83	57.70	55.61	43.46
1000 m	56.50	n/a	n/a	44.70	n/a	n/a	n/a	n/a
1500 m	n/a	n/a	n/a	n/a	54.49	n/a	n/a	43.34
3000 m	58.43	n/a	n/a	45.16	47.90	n/a	n/a	43.08
5000 m	57.66	59.74	n/a	45.66	n/a	n/a	n/a	n/a
10 k	55.29	58.71	n/a	44.69	53.22	55.83	n/a	42.91
High jump	59.58	60.60	62.03	45.41	64.05	69.68	n/a	41.92
Long jump	59.64	70.13	n/a	47.48	56.82	68.23	n/a	43.55
Triple jump	60.54	66.27	n/a	47.29	n/a	n/a	n/a	n/a
Pole vault	54.46	58.88	59.03	43.97	n/a	n/a	n/a	n/a
Discus	59.04	60.60	62.47	47.34	55.43	56.56	56.51	45.39
Shot put	58.81	61.66	65.45	46.74	55.95	58.60	61.99	44.90
Javelin	59.29	64.88	n/a	47.45	55.32	65.07	n/a	45.45
Average	58.28	62.67	62.24	46.47	56.46	61.49	60.07	44.08

### Annual percentage rate of performance decline

Average performance declines in percent per year are shown in Table 2 to allow comparison with the literature. In athletes with 10 results and more, the average decline before the age of 70 was 0.83%/year for men and 0.91%/year for women with the steepest decline of 1.44%/year in the women’s javelin throw, followed by 1.37%/year in the women’s discus throw. After the age of 70 years, decline rates increased to 1.40%/year in the men and 1.31%/year in the women. This means that the decline after age 70 is 1.7 times as steep in the men and 1.4 times as steep in the women compared with younger ages. To compare the decline functions between CS and LN data, as well as women and men, ANCOVAS were conducted on the data normalized to age 35. These revealed no differences in the rate of performance decline in 100 m, 200 m, 800 m, 1000 m (men only), 10 km, long jump, triple jump (men only) and javelin throw. Although in the pooled data, there appeared a significant difference in the rate of decline between CS and LN data for the 400 m, 1500 m (women only), 3000 m, 5000 m (men only), high jump and discus throw, no significant differences were seen when analyzing the athletes under and over 70 years separately. In the pole vault (men only), steeper slopes in the CS data were found in the athletes under 70 years of age (*p* = 0.004), but not in the older ones (*p* = 0.727). The same is true for the shot put (athletes under 70 years: *p* < 0.001 and over 70 *p* = 0.454). Overall, these results indicate that the CS and LN data show similar rates of decline. In both CS and LN data, there were no significant differences in the rate of decline between men and women.

### Individuals with 30 and more results

**Fig. 2:**
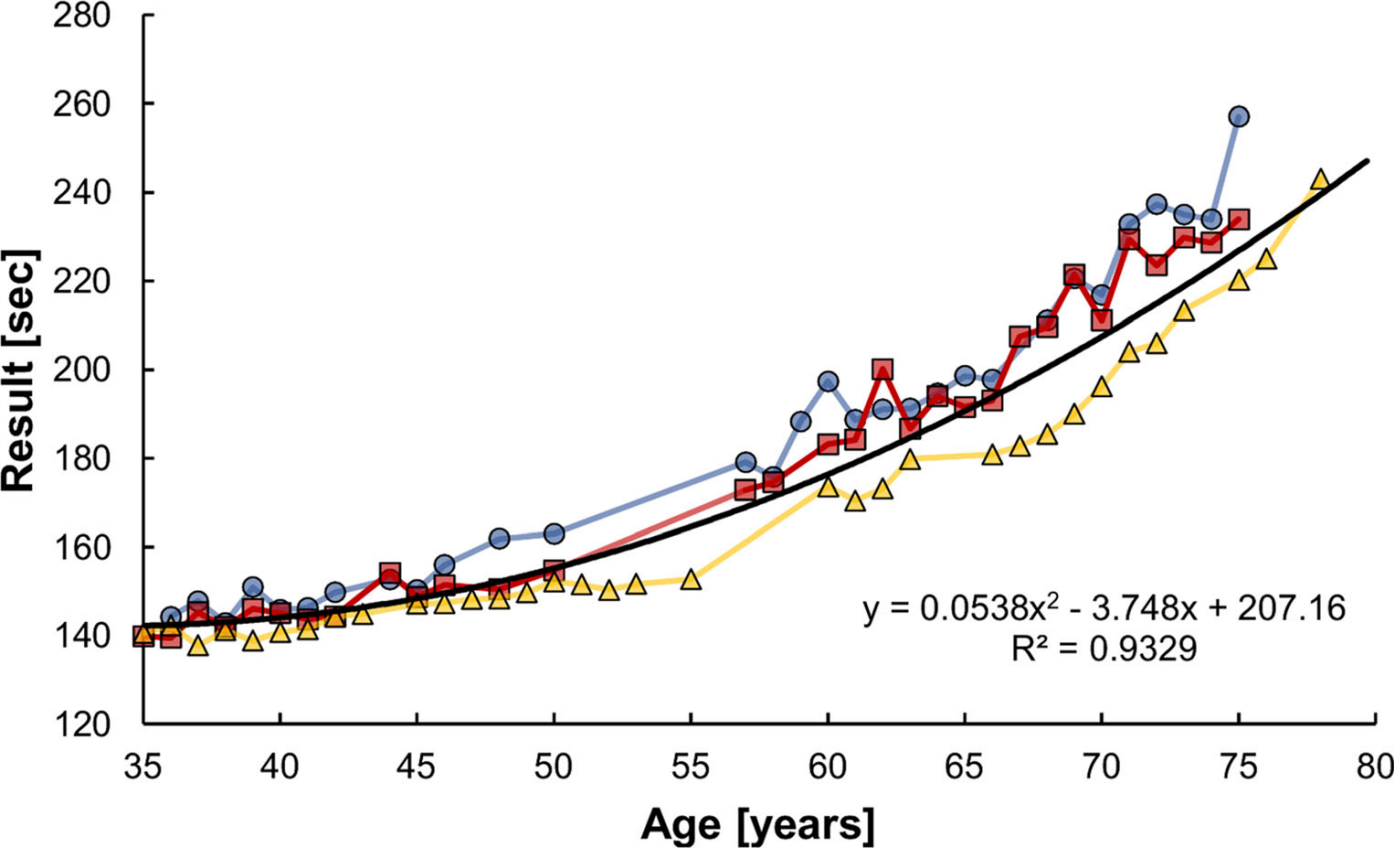
Performance declines with age in the women’s 800 m. Only the data of the three athletes with 30 results and more in the data set are shown. Two of the three athletes (blue circles, 30 results; red squares, 31 results) were born in 1943 and the third in 1940 (yellow triangles, 34 results)

Figure 2 shows the performance declines of the three female athletes with 30 results and more in the 800-m data set. The *R*^2^ of the second-order polynomial regression is particularly high at 0.93, indicating a small difference in performance among the three athletes and a similar trajectory of the age-related decline in performance.

## Discussion

A data set with 83,209 master athletics results was derived from the ‘Swedish Veteran Athletics’ annual ranking lists and analyzed to study differences in the trajectory of the age-related performance decline between CS and LN data. The main findings are that (1) the performance at any age is higher in LN than CS data, (2) the decline rates were similar in longitudinal and cross-sectional data, (3) although men had a higher performance at any age than women in all disciplines, (4) the rate of decline was similar in men and women, (5) performance declines after age 70 were on average 1.7 times as steep in the men and 1.4 times as steep in the women compared with the decline between 35 and 69 years, (6) the average age was higher in the LN than CS data set and (7) the women’s discus and javelin throw before age 60 years appears to show the steepest and shot put (both sexes) the least steep slopes in the data set.

### Performance decline rates

Rates of physical performance declines with age have been extensively studied over the past decades, but no paper has ever presented a LN data set of the size as in the present study. In a previous paper, we analyzed a large CS data set of German master athletics results, identifying the women’s javelin throw (decline rate before age 70, 1.69%/year) and the men’s pole vault (1.42%/year) as the disciplines with the steepest decline [1]. In comparison, decline rates in the present LN data were 1.44%/year in the women’s javelin throw before the age of 70 years and 1.01%/year in the LN data for the men’s pole vault. The slightly lower decline rates in the present data set could either be explained by the different normalization (previous study normalized to the average of the performance of the 30-year-old athletes, and here normalized to the 35-year-old athletes) or by differences between the German and Swedish population, or be due to having LN compared with CS data.

Steeper declines at higher age have been reported in many studies with both CS and LN data [1, 7, 11, 16, 19, 20]. In the present study, performance declines after age 70 were on average 1.7 times as steep in the men and 1.4 times as steep in the women compared with the decline between 35 and 69 years. In our previous CS data set [1], we found a similar accelerated decline. In another CS analysis, we found that the performance decline was even more than three times as steep in athletes ≥ 80 compared with athletes 30–69 years [2]. These observations indicate a progressive acceleration of the decline in performance during ageing that is better described with a polynomial rather than linear function (see Online Resources 2 and 3), something also seen by others [8, 11, 20], and in both our CS and LN data sets.

### Differences between CS and LN data

We are the first to compare the trajectories of the age-related declines in performance in LN data (athletes with 10, 15, 20 data points and in 800 m women even 30 individual results) in several disciplines with that derived from CS data in the same data set. It appeared that the performance was lower in the CS than the LN data, irrespective of discipline and age. Yet, the annual percentage rate of decline did not differ significantly between CS and LN data, or whether there were 10, 15, 20 or 30 results. To our knowledge, this difference in performance between CS and LN data has not been previously explicitly reported, even though it can be seen on the plots of Young et al. [11]. In the first study ever published on potential differences in annual rate of % performance decline in master athletes derived from CS and LN data, it was seen that the CS decline was approximately twice as steep as the LN decline over a 5-year period in a data set of Canadian Masters track rankings [7]. We did not see a significant difference in the annual percent performance decline derived from CS and LN data. The discrepancy with our results may well be the relatively small sample size and short period in the study by Stones and Kozma [7] compared with our sample size. Whatever the cause of the discrepancy, our observation that the annual percentage rate of decline in performance is similar in CS and LN data suggests that CS data can indeed give a good impression of the decline in performance in a population.

Another important finding of the present study is that the average age of the participants in the LN dataset was higher than that in the CS data set. This is in contrast to the lower age of the participants in the LN than in the CS data in the study by Young et al. [11]. They attributed the discrepancy between CS and LN data by their low samples size in the LN data, and a comparison of different data sets, while in our study the CS and LN data came from the same data set. One explanation for the higher average age in the LN than CS data is that those individuals that are successful are probably enticed to continue competitions, while one-off competitors that are not successful are less motivated to return. This is supported by the observation that repeaters had, on average, a better performance at any age, irrespective of discipline, than one-off competitors. Having said that, in our previous study in the oldest old athletes [2], we did not see a difference in performance at age 80 between those still competing at age 85 and those who had stopped competing.

### Differences between LN data and world record data

Gava et al. [22] compared the men’s world records of 16 events, while Baker et al. [23] compared the world records for 17 disciplines in both sexes. The analyses of Baker et al. [23] showed an almost linear decline in performance in the jumps without an acceleration in the performance decline at higher age, and only a slightly accelerated decline in the throws. The paper by Gava et al. [22] even shows a slight deceleration in the decline in performance in the throws after age 80 years. This is in contrast to the accelerated decline after the age of 70 we observed here, and that has also been reported by others and us previously [1, 2, 8, 11, 20]. An explanation for this difference was given by Rubin et al. [19], who showed in LN data of master swimmers that individuals who start to swim later in life may show a shallower decline in performance than the world records due to the training-induced improvement in their physiology and improvements in technique. This is mostly the case in those athletes who have not competed their entire life, but picked up competitive sports later.

In addition to these differences, the initial increase in performance around age 40 and 45, which we found in some events, was not seen in the world records. An advantage of the analysis of world records over rankings is that, while the oldest women in our data set were in their late 80s, world records are available up until 100 years and older [23]. However, they represent the result of a rather small population only, i.e. the world record holders, and do not reflect how the performance of the average athlete changes with age.

### Individuals with many LN results

Young and Starkes [8] published LN running performance data of 1500 m (7 athletes, 20.1 years sampled, 32.6 to 52.7 years of age) and 10 km (9 athletes, 18.9 years sampled, 32.5 to 51.4 years of age), that to our knowledge is currently the largest LN performance data set in running. In comparison, the present data set includes 61 athletes with 20 or more results (30 men and 31 women) and 312 athletes with 15 or more data-points, making it by far the largest LN data set with the most long-term data ever published in the context of master athletics. The decline rates and regression equations computed in the present study therefore seem to be the most accurate so far, at least for a northern European population. It might well be that average performances and decline rates differ in separate nations and continents, due to differences in lifestyle and typical body characteristics. The next step in research should therefore be a comparison of data sets from around the world.

### Outliers

In the LN part of our study, several individuals had an extremely low performance, despite competing over many years. This is for example the case in 100 m and 200 m women (Online Resource 2). We have excluded these outliers from the analysis, as their data does not seem to be representative of the typical master athlete. The times indicate that the person did not run, but walk. Apart from these extreme outliers, however, several individuals can be seen in the data set who perform a bit worse than the others initially and then have a much steeper performance decline with aging (example: 100 m men). This phenomenon might be caused by underlying health issues that exacerbate with aging, and we suggest to look into this phenomenon further to identify the causes for the faster declines. This can, however, not be accomplished by analyzing ranking data, but interviews of athletes will be required.

### Sex differences

Social and cultural factors are among the reasons why only 22% of data in the present study are from female athletes. Women are only half as likely to participate in competitive sports compared with men [24] and reach approximately 80% of the men’s performance [23]. Female participation in competitive athletics only started to be a mass phenomenon in the second half of the twentieth century after the 1948 Summer Olympic Games, where 385 female athletes competed [25]. In the present study, a particularly steep decline in the women’s discus and javelin throwing performance was found before the age of 60 years. The same was previously observed in a large CS data set from Germany [1]. The steep performance decline in women can already be observed before and independent of the first decrease in the javelin’s weight at age 50 years (35–49 years: 600 g, 50–74 years: 500 g) and appears therefore independent of the changing javelin characteristics with age. In discus throw, the same weight is used from 35 to 74 years. A cause of this particularly fast and early drop in throwing performance in women could be of endocrine nature. It is unclear, however, why such fast performance declines are then not observed in shot put or in the jumps. High jump, for example, depends on agility and technique just as much as javelin and discus throw. The main difference between these events is upper body strength. While sarcopenia and losses in muscle power are usually problems of the old age groups, our findings indicate that a drop might occur in younger women, too, and should be studied further. Apart from these findings in the throws, our results indicate that men and women age similarly. This means that the performance decline rates in percent per year are similar in men and women.

### Historical changes

Over the years, many changes will undoubtedly have led to an improved performance. These include changes in throwing devices, poles, the surface material of running tracks, shoes and sprint spikes, but also developments in techniques, such as the introduction of the Fosbury flop in the high jump. These changes could not be considered in the present analysis, as it is unclear to what degree these issues have improved results and most were probably introduced gradually. Considering that most of these changes have improved the performance, it must be concluded that the performance declines presented in this paper are probably slightly underestimated.

### Strength and weaknesses

The main strength of the present study is the huge LN data set containing athletes with as many as 10, 15, 20 and even 30 LN data-points. Weaknesses include that only one country (Sweden) was studied, which may limit the validity for other countries and continents. Another weakness is the much lower number of women (23.3%) than men in the data set that seems to be attributable to social factors. 

## Conclusions

We analyzed the largest LN master track and field data set ever published. LN master athletics data not only has a higher average performance, but also a higher average age compared with CS data. While we found that the performance at any age was on average better in the LN than the CS data set, the trajectory of the age-related decline in performance was similar in CS and LN data sets, as well as in men and women. This indicates that while CS data sets may underestimate performance, they are adequate to assess the trajectory of the age-related decline in performance in the athlete population.

## Electronic supplementary material

ESM 1 Python-scripts (scraper, parser and combiner/formatter) (DOCX 26 kb)

ESM 2 CS and LN data shown for each of the 16 track and field events and both sexes. LN data is shown for athletes with 10, 15, 20 and 30 or more results in the data set, if 3 or more athletes are available. (PDF 3035 kb)

ESM 3 Supplementary Figs. 1 and 2 showing a comparison of the pooled regression lines of 10, 15, 20 and 30 results. (PDF 955 kb)

## Data Availability

The database of ‘Swedish Veteran Athletics’ (http://www.friidrott.info/veteran/index.php) is publicly available.

## Footnotes

1 https://world-masters-athletics.com/wp-content/uploads/2018/03/appendix-WMA-22.03.2018.pdf
