# Supplementary material for: Longitudinal trends in master track and field performance throughout the aging process: 83,209 results from Sweden in 16 athletics disciplines
Source: GeroScience. 2020 Oct 13;42(6):1609–20. doi: 10.1007/s11357-020-00275-0 (PMC7732911; doi:10.1007/s11357-020-00275-0)
Supplement: Supplementary file 1 — Python-scripts (scraper, parser and combiner/formatter) (DOCX 26 kb) [file 11357_2020_275_MOESM1_ESM.docx]

**Online Resource 1**

**GeroScience**

Longitudinal trends in master track and field performance throughout the aging process: 83,209 results from Sweden in 16 athletics disciplines

Ganse B*, Kleerekoper A, Knobe M, Hildebrand F, Degens H

* Manchester Metropolitan University, b.ganse@mmu.ac.uk

**This file contains the code of the scraper, parser and combiner/formatter in Python.**

1. **Scraper:**

from bs4 import BeautifulSoup

import urllib3

import sys

import time

def getcode(dictionary, desired_klass):

for code, klass in dictionary.items():

if klass == desired_klass:

return code

missing_years = [1908,1906,1905,1904]

klass_map_K = {13:35,14:40,15:45,16:50,17:55,18:60,19:65,20:70,21:75,22:80,23:85,24:90,531:95}

klass_map_M = {27:35,28:40,29:45,30:50,31:55,32:60,33:65,34:70,35:75,36:80,37:85,38:90,530:95}

#for gender in ['K','M']:

gender = 'M'

#for year in range(1900,2020,1):

for year in range(1989,2020,1):

if year not in missing_years:

for klass in range(35,100,5):

if gender =='K':

code = getcode(klass_map_K, klass)

else:

code = getcode(klass_map_M, klass)

url = 'http://www.friidrott.info/veteran/showresult.php?toplist=basta&year='+str(year)+'&miljo=Utomhus&klass='+str(code)+'&kon='+str(gender)

print(url)

http = urllib3.PoolManager()

r = http.request('GET', url)

filename = gender+"_"+str(klass)+"_"+str(year)+".html"

f = open("pages/"+filename,'wb')

f.write(r.data)

f.close()

#time.sleep(2.5)

1. **Parser:**

from bs4 import BeautifulSoup

from os import listdir

import csv

import sys

import pandas as pd

headers = ["Event","Agegroup","Resultat","Vind","Namn","ID","Född","Klubb","Klass","Datum","Ort","Arena","Kommentar"]

for file in listdir('pages'):

print("processing file: "+file)

with open('pages/'+file, encoding="utf8") as html:

soup = BeautifulSoup(html, 'html5lib')

if soup.find("table") is not None:

table = soup.find("table")

## remove subtables

for tbl in table('table'):

tbl.decompose()

output_rows = []

currentEvent = ""

if table.find_all('tr') is not None:

for table_row in table.find_all('tr'):

columns = table_row.find_all('td')

output_row = []

output_row.append(file[:-5].split('_')[1])

for column in columns:

if column.text != '\xa0':

if column.find('h4') != None:

currentEvent = column.text

else:

output_row.append(column.text)

if len(output_row) == 4:

id = column.find('a', href=True)['href'].replace('showuser.php?uid=','')

output_row.append(id)

if (len(output_row) > 0):

output_row.insert(0, currentEvent)

if len(output_row) == len(headers):

output_rows.append(output_row)

try:

df = pd.DataFrame(output_rows, columns=headers)

except:

[print(len(x)) for x in output_rows]

[print(x) for x in output_rows]

sys.exit()

try:

#print('agegroup: '+str(file[:-5].split('_')[1]))

#print('year: '+str(int(file[:-5].split('_')[2])))

century = '19'

if int(file[:-5].split('_')[1])+1900 - int(file[:-5].split('_')[2]) > 0:

century = '18'

#print('century: '+str(century))

#df['dob'] = pd.to_datetime(century+df['Född'].str[1:]+"/01/01", format='%Y/%m/%d')

df['dob'] = pd.to_datetime(century+df['Född'].str.replace('-','')+"/01/01", format='%Y/%m/%d')

except Exception as e:

print("Failed to calculate dob. Exiting")

print(e)

df.to_csv("dump.csv")

sys.exit()

try:

df['Datum'] = pd.to_datetime(df['Datum'], format='%Y/%m/%d')

except:

df['Datum'] = pd.to_datetime(df['Datum'].str[:4])

df['age'] = (df['Datum'] - df['dob']).astype('timedelta64[Y]')

df.to_excel('dfs_second/'+file[:-5]+'.xlsx')

1. **Combiner/formatter:**

import pandas as pd

import numpy as np

from os import listdir

from pandas import ExcelWriter

import re

womens_events = ['Kula','Diskus','Spjut','Slägga','Höjd','Längd','Stav','Tresteg','100m','200m','400m','800m','1500m','3000m','10000m']

mens_events = ['Kula','Diskus','Spjut','Slägga','Höjd','Längd','Stav','Tresteg','100m','200m','400m','800m','1000m','3000m','5000m','10000m']

def bundleEvents(event_name):

if "kula" in str.lower(event_name):

return "Kula"

if "diskus" in str.lower(event_name):

return "Diskus"

if "spjut" in str.lower(event_name):

return "Spjut"

if "slägga" in str.lower(event_name):

return "slägga"

else:

return event_name

def toseconds(time):

parts = time.split('.')

minutes = parts[0]

seconds = int(parts[1]) + int(minutes)*60

if len(parts) == 3:

newtime = str(seconds) + '.' + parts[2]

else:

newtime = str(seconds)

return newtime

## M = men

## K = women

gender = 'K'

df = pd.DataFrame()

for file in listdir('dfs_second'):

if gender in file:

print("Processing file: "+str(file))

newdf = pd.read_excel('dfs_second/'+file, index_col=0)

df = df.append(newdf)

## Remove unwanted events

df = df[~df['Event'].str.contains('ua|vh|gamla', regex=True, flags = re.IGNORECASE)]

df['Event'] = df['Event'].apply(bundleEvents)

if gender == 'M':

df = df[df['Event'].isin(mens_events)]

else:

df = df[df['Event'].isin(womens_events)]

events = df['Event'].unique()

with ExcelWriter('BergitaData_'+gender+'.xlsx', engine='xlsxwriter') as writer:

for event in events:

eventSubset = df[['Agegroup','Event','ID','Resultat','Född','age','Datum','dob']][df['Event']==event]

# correct result to right format

if event[-1] == 'm':

print(event, ' dealing with a race')

if int(event[:-1]) < 800:

## times in seconds - only need to remove any "+" signs or "s"

eventSubset['Resultat'] = eventSubset['Resultat'].astype('str').apply(lambda x: x.replace('+','').replace('s', '').replace('m',''))

else:

## times in minutes

eventSubset['Resultat'] = eventSubset['Resultat'].astype('str').apply(lambda x: x.replace('+','').replace('m',''))

eventSubset['Resultat'] = eventSubset['Resultat'].apply(toseconds)

else:

eventSubset['Resultat'] = eventSubset['Resultat'].astype('str').apply(lambda x: x.replace('+','').replace('m', ''))

## drop rows where the result cannot be put into a time or distance/height

print('Before dropping:', eventSubset.shape)

eventSubset['Resultat'] = pd.to_numeric(eventSubset['Resultat'], errors='coerce')

print('After dropping: ', eventSubset.shape)

## for now drop duplicates

#eventSubset = eventSubset.drop_duplicates(subset=['ID','age'], keep='last')

## keep only best result for given Event, Name, Datum and dob

if event[-1] == 'm':

## doing a race so want smallest time

eventSubset = eventSubset.sort_values(by='Resultat', ascending=True).drop_duplicates(subset=['ID','age'], keep='first')

else:

## throw or jump so want longest

eventSubset = eventSubset.sort_values(by='Resultat', ascending=False).drop_duplicates(subset=['ID','age'], keep='first')

## remove rows that are erroneous either because negative age or because age doesn't fit with agegroup

## for now, require ages to be within 5 years (inclusive) of agegroup, so agegroup 45 includes anyone aged between 40 and 50

eventSubset = eventSubset[eventSubset['age'].astype(int) > 0]

eventSubset = eventSubset[np.abs(eventSubset['age'].astype(int)-eventSubset['Agegroup'].astype(int)) <= 5]

print(eventSubset[['ID','Född','dob','Datum','age']][eventSubset['age'] <0])

pivoted = eventSubset.pivot(index='ID', columns='age',values='Resultat')

out = pivoted.merge(eventSubset[['ID','Född','dob']], how='inner', on='ID').drop_duplicates()

try:

out.to_excel(writer, sheet_name=event, index=False)

except:

print("Failed to write event: "+str(event))

print("It contained "+str(len(out))+" rows")

pass

writer.save()
