# Supplementary material for: Longitudinal trends in master track and field performance throughout the aging process: 83,209 results from Sweden in 16 athletics disciplines
Source: GeroScience. 2020 Oct 13;42(6):1609–20. doi: 10.1007/s11357-020-00275-0 (PMC7732911; doi:10.1007/s11357-020-00275-0)

Online Resource 2

GeroScience

Longitudinal trends in master track and field performance throughout the aging process: 83,209 results from Sweden in 16 athletics disciplines.

Ganse B, Kleerekoper A, Knobe M, Hildebrand F, Degens H

Corresponding author: b.ganse@mmu.ac.uk

Index:

1 – 3: 100m men
4 – 7: 100m women
8 – 10: 200m men
11 – 14: 200m women
15 – 18: 400m men
19 – 21: 400m women
22 – 24: 800m men
25 – 29: 800m women
30 – 31: 1000m men
32 – 33: 1500m women
34 – 35: 3000m men
36 – 37: 3000m women
38 – 40: 5000m men
41 – 43: 10k men
44 – 46: 10k women
47 – 50: High jump men
51 – 53: High jump women
54 – 56: Long jump men
57 – 59: Long jump women
60 – 62: Triple jump men
63 – 66: Pole vault men
67 – 70: Discus throw men
71 – 74: Discus throw women
75 – 78: Shot put men
79 – 82: Shot put women
83 – 85: Javelin throw men
86 – 88: Javelin throw women

1

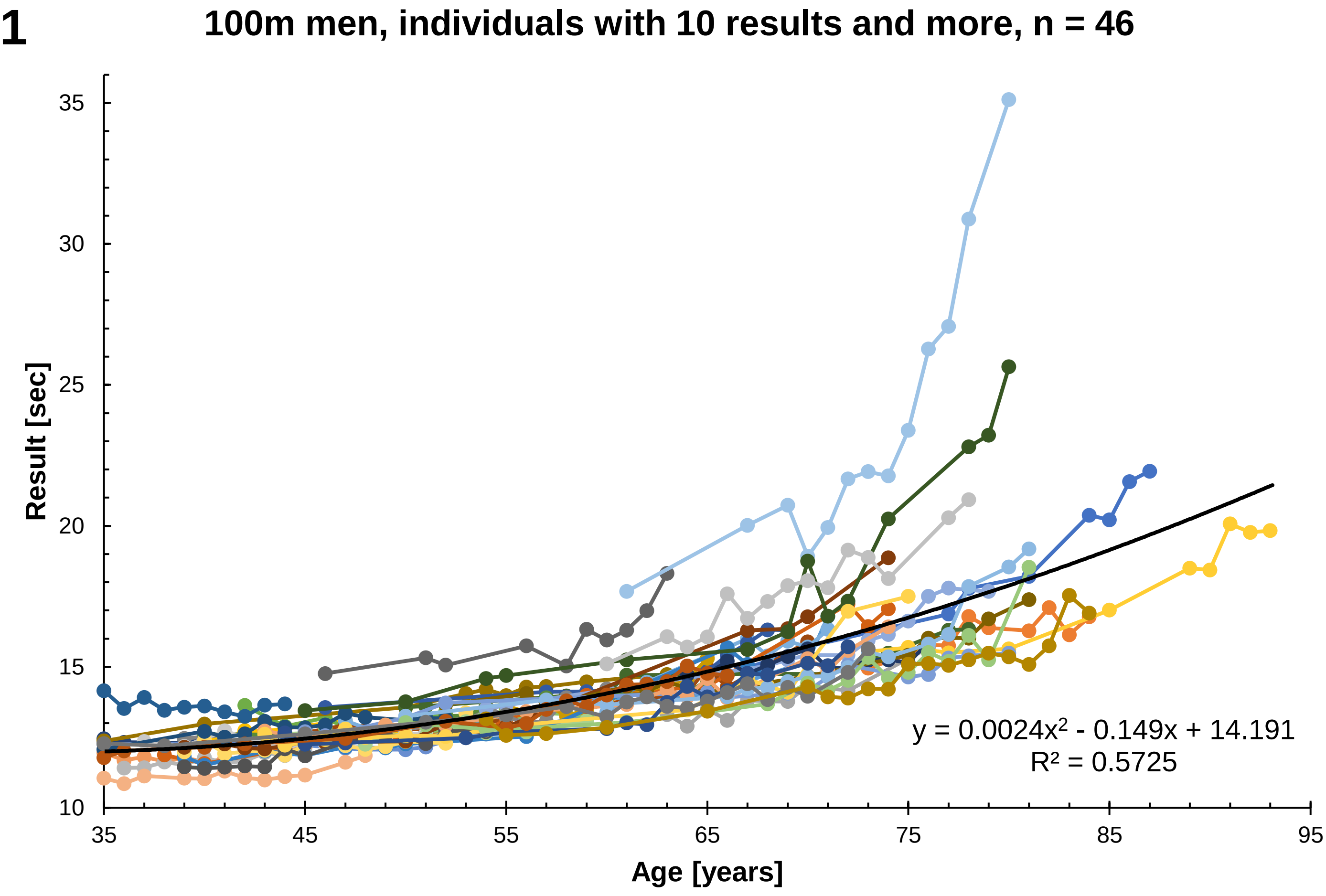
100m men, individuals with 10 results and more, n = 46
Result [sec]
35
30
25
20
15
10
35
45
55
65
75
85
95
Age [years]
y = 0.0024x² - 0.149x + 14.191
R² = 0.5725

2

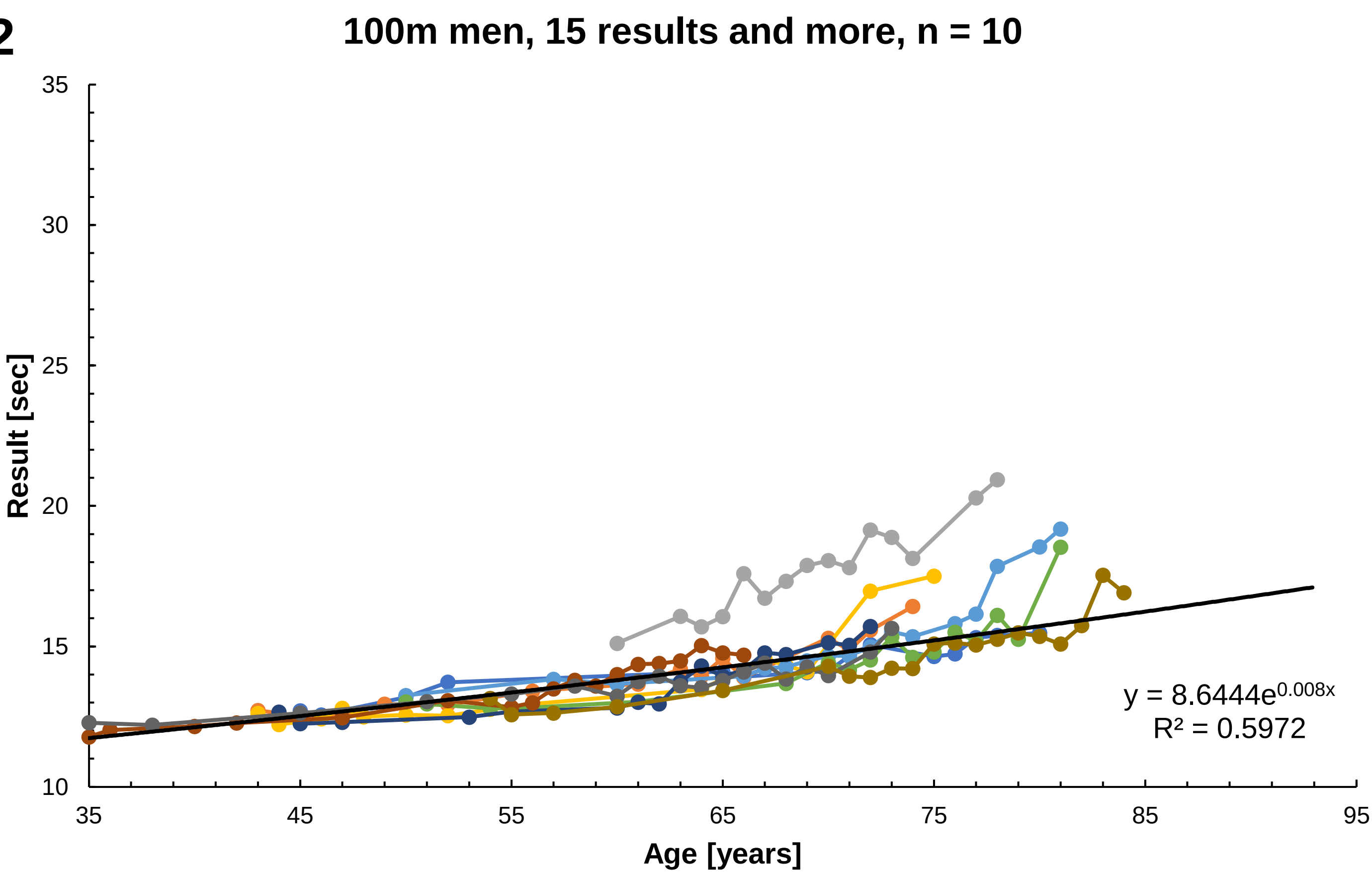
100m men, 15 results and more, n = 10
Result [sec]
35
30
25
20
15
10
35
45
55
65
75
85
95
Age [years]
y = 8.6444e^0.008x
R² = 0.5972

3

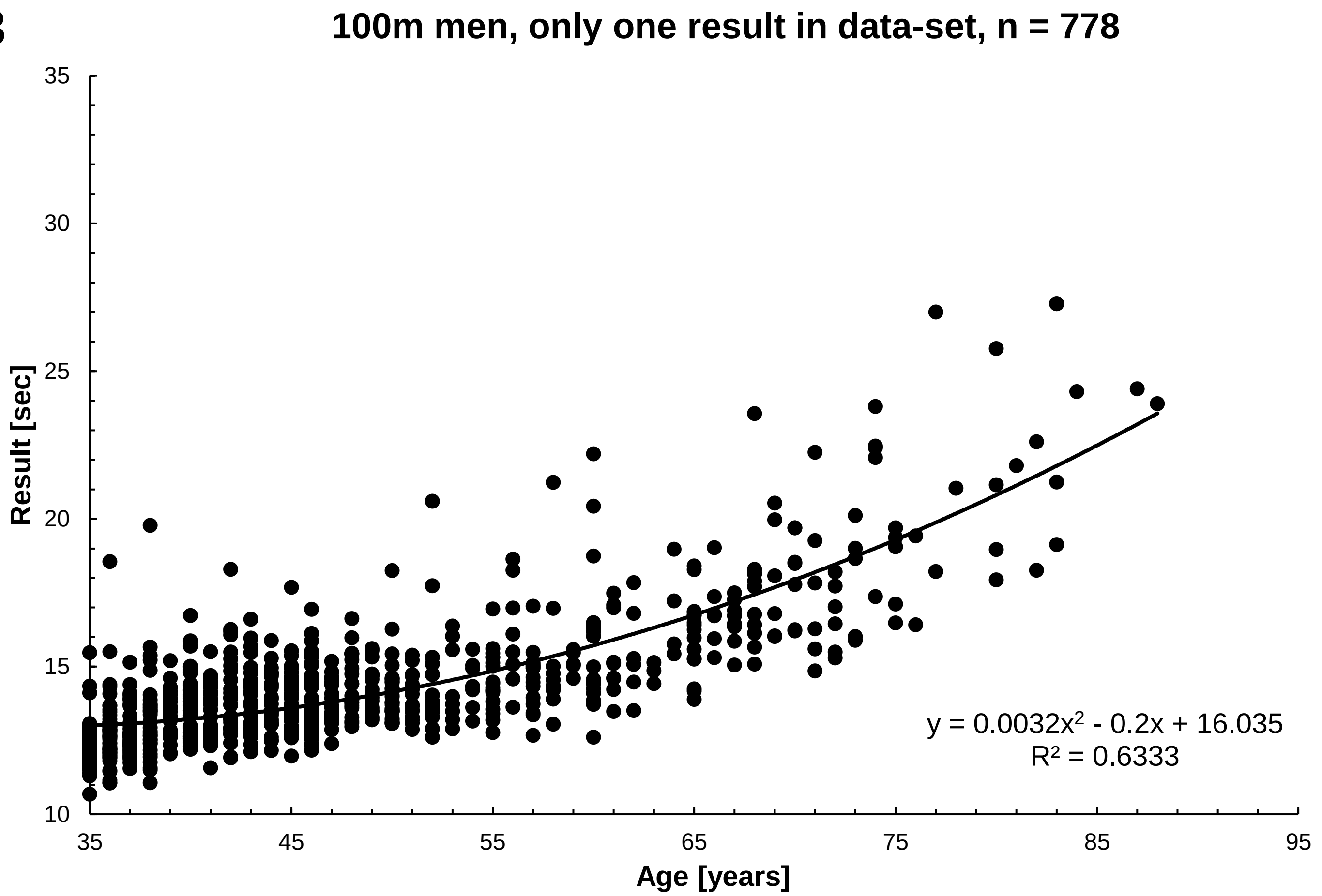
100m men, only one result in data-set, n = 778
Result [sec]
Age [years]
35
30
25
20
15
10
35
45
55
65
75
85
95
y = 0.0032x² - 0.2x + 16.035
R² = 0.6333

4

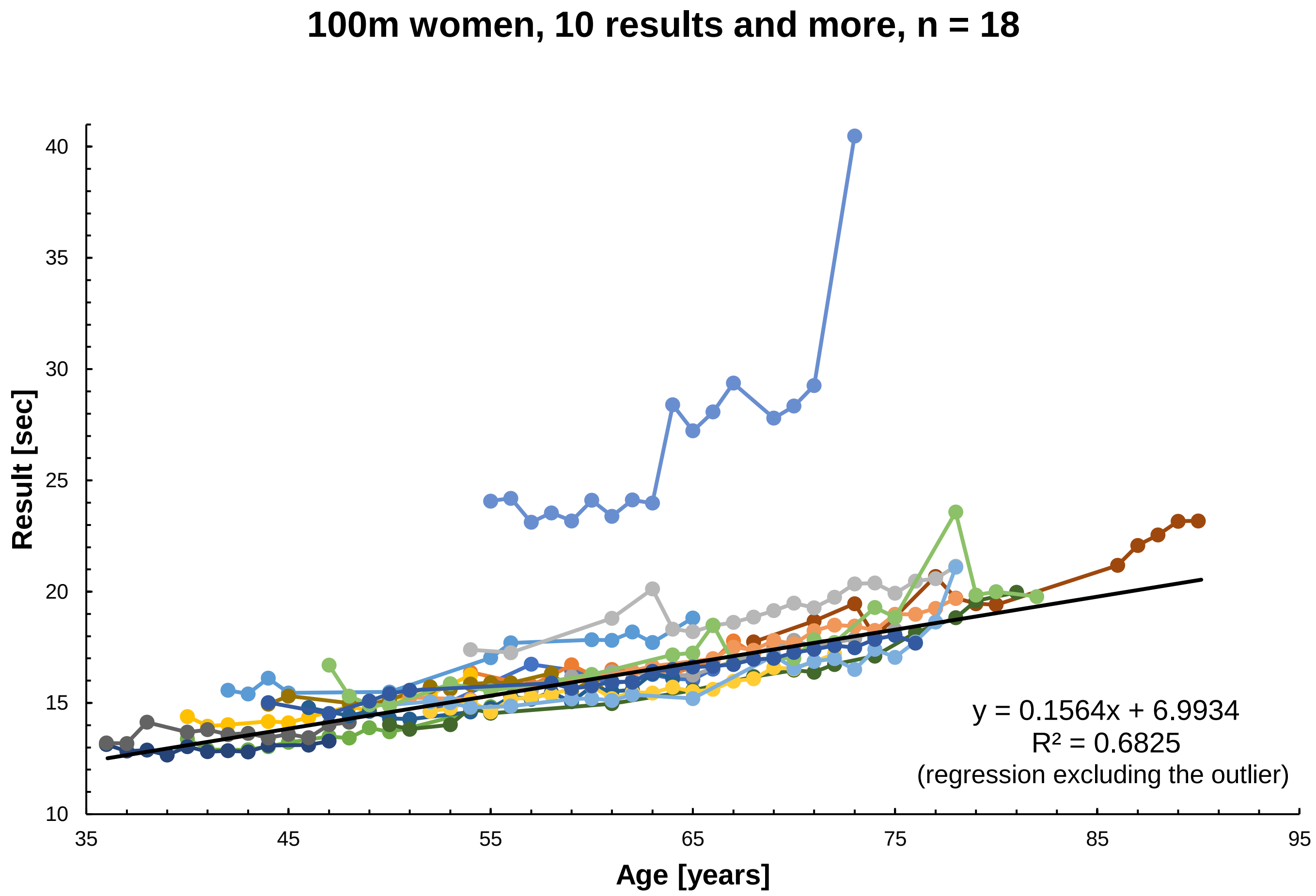
100m women, 10 results and more, n = 18
Result [sec]
Age [years]
40
35
30
25
20
15
10
35
45
55
65
75
85
95
y = 0.1564x + 6.9934
R² = 0.6825
(regression excluding the outlier)

5

100m women, 15 results and more, n = 9

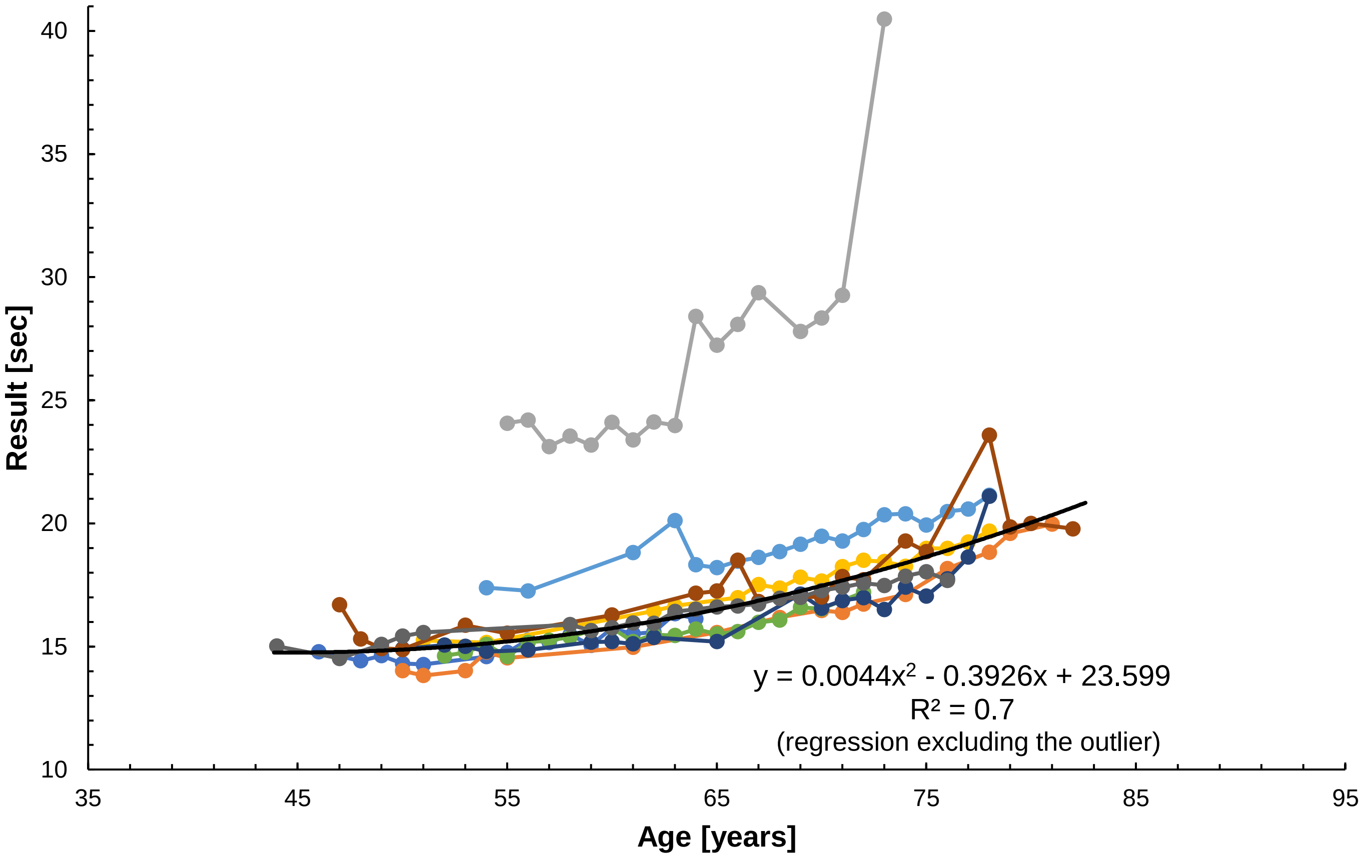

6

100m women, 20 results and more, n = 4

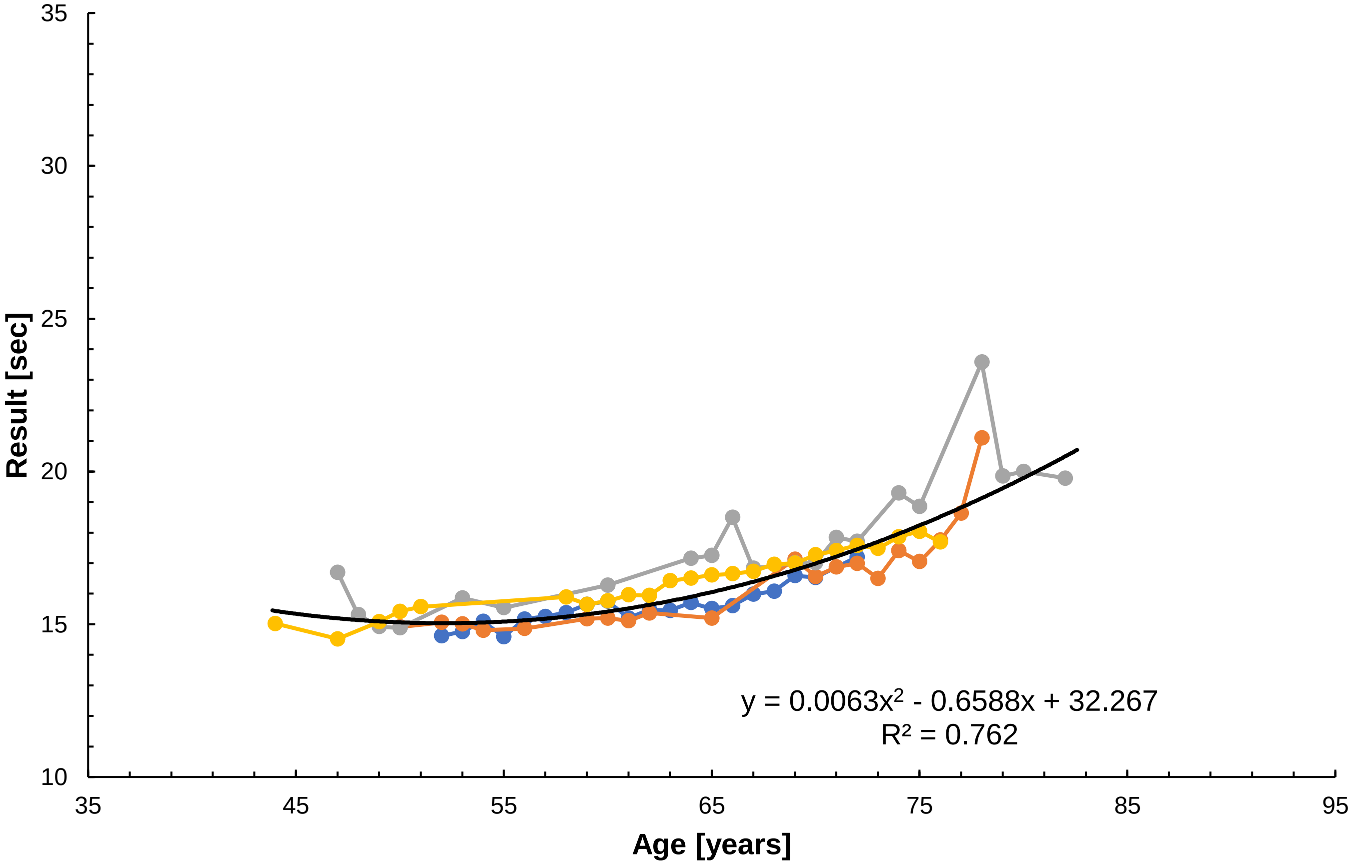

7

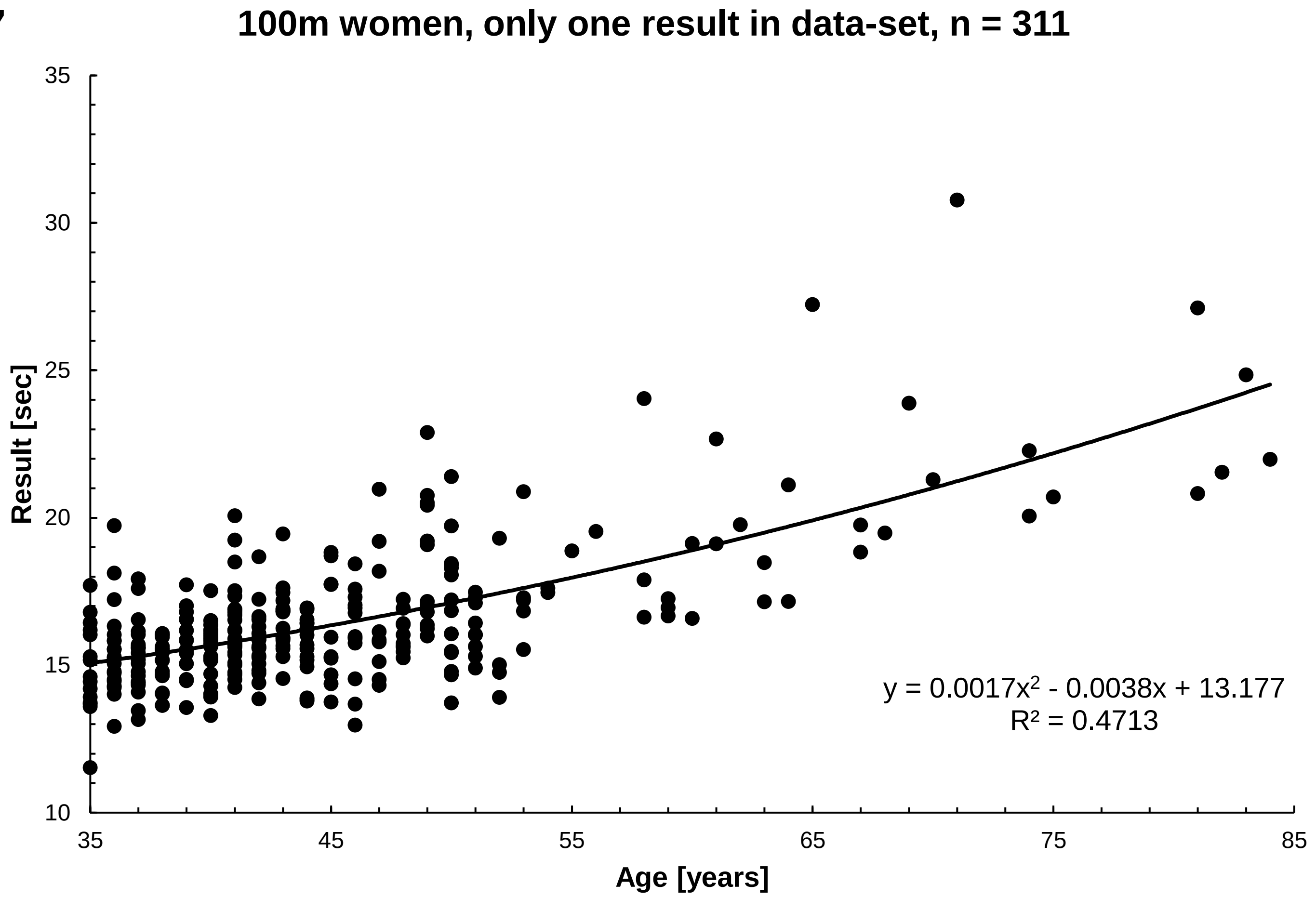
100m women, only one result in data-set, n = 311
Result [sec]
35
30
25
20
15
10
35
45
55
65
75
85
Age [years]
y = 0.0017x² - 0.0038x + 13.177
R² = 0.4713

8

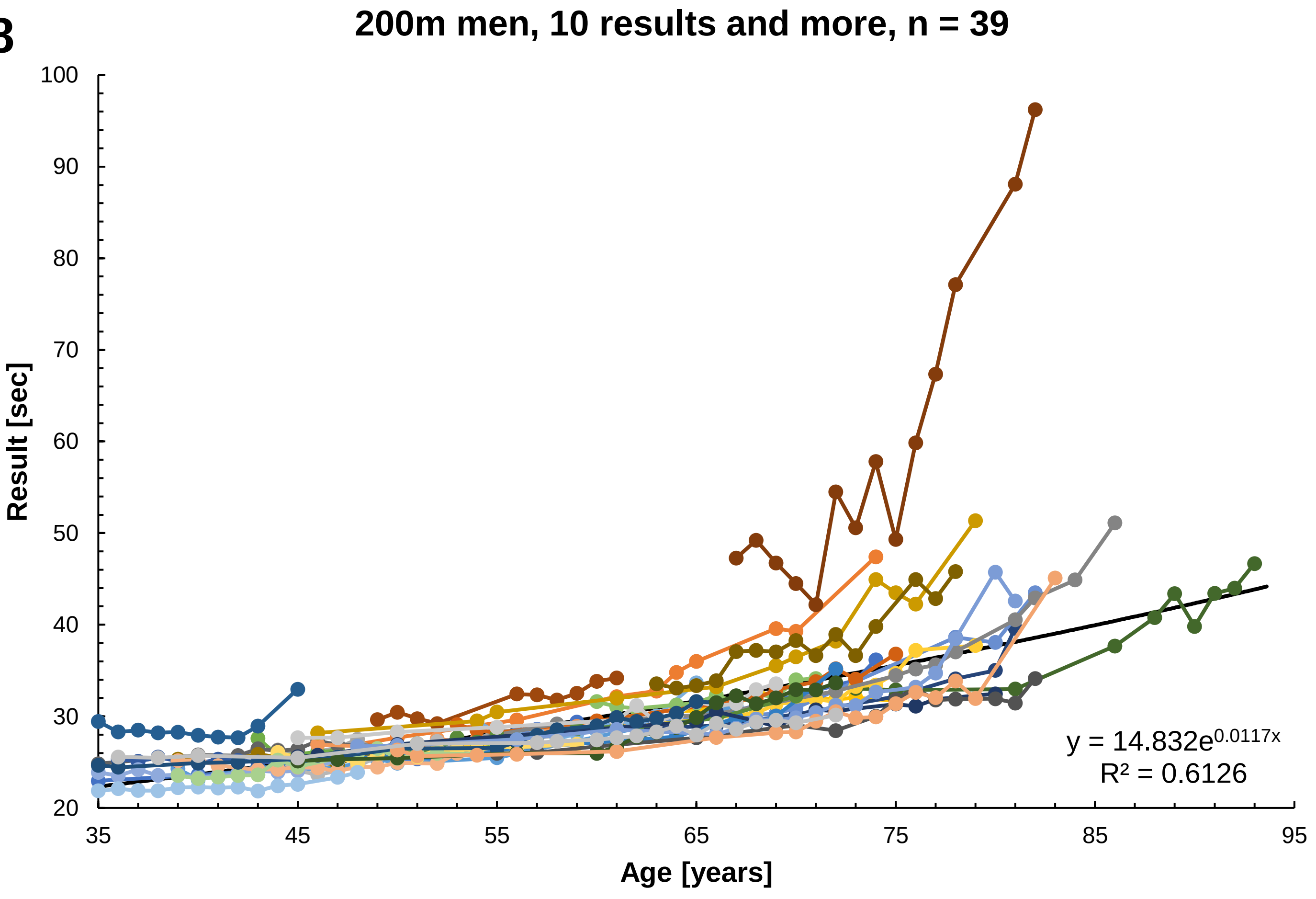
200m men, 10 results and more, n = 39
Result [sec]
100
90
80
70
60
50
40
30
20
35
45
55
65
75
85
95
Age [years]
y = 14.832e^0.0117x
R² = 0.6126

9

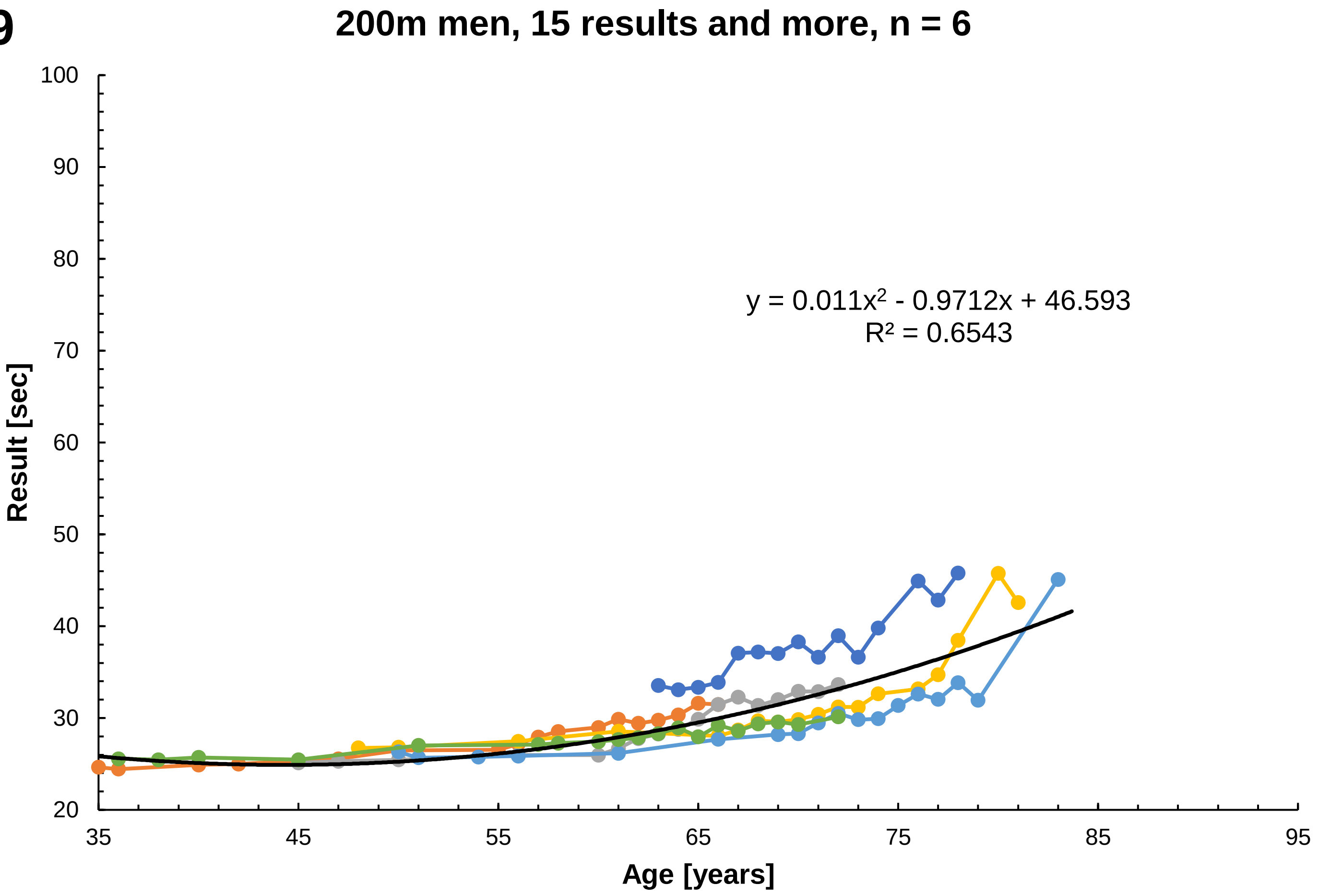

200m men, 15 results and more, n = 6
Result [sec]
Age [years]
y = 0.011x² - 0.9712x + 46.593
R² = 0.6543

10

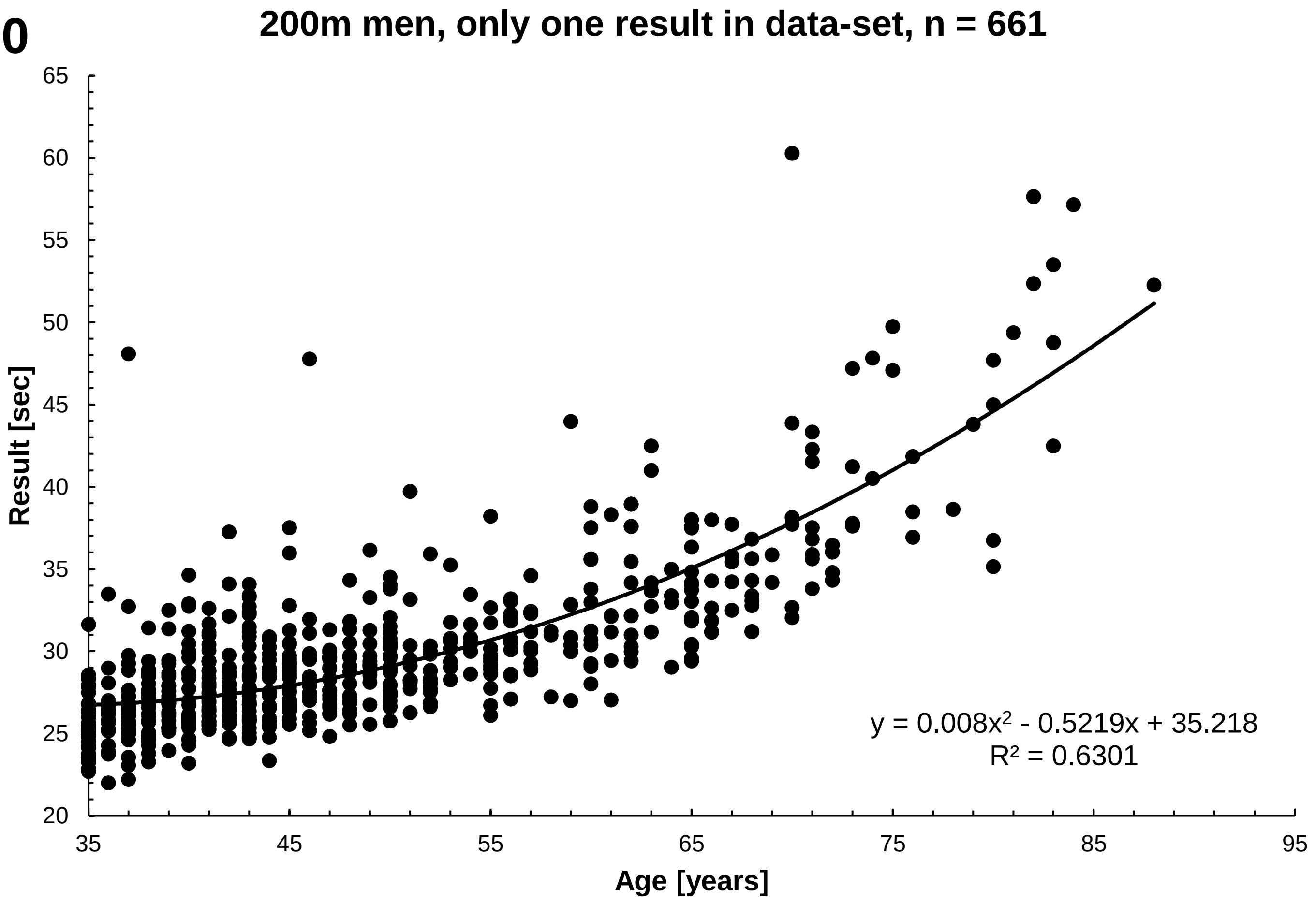

200m men, only one result in data-set, n = 661
Result [sec]
Age [years]
y = 0.008x² - 0.5219x + 35.218
R² = 0.6301

11

200m women, 10 results and more, n = 15

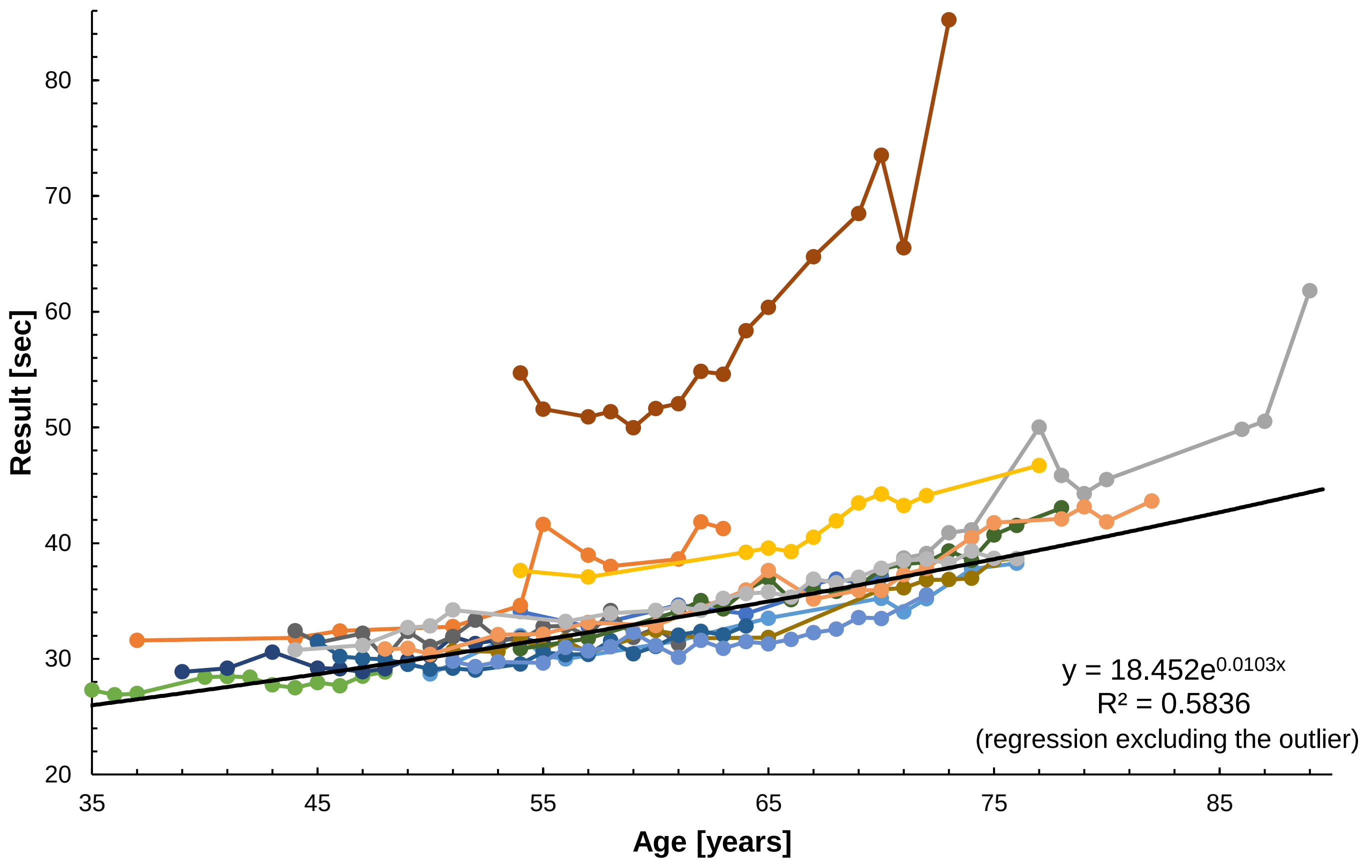

12

200m women, 15 results and more, n = 8

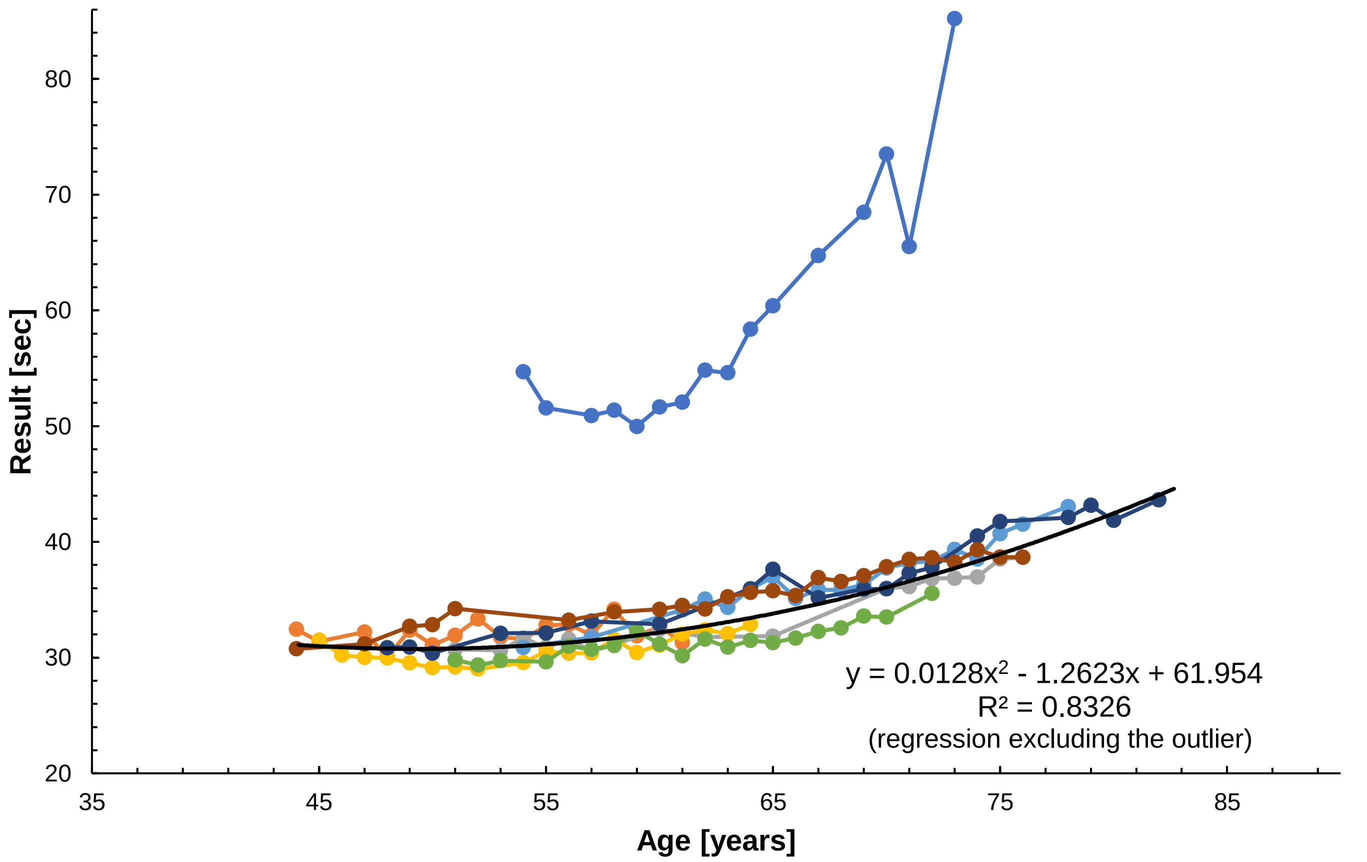

13

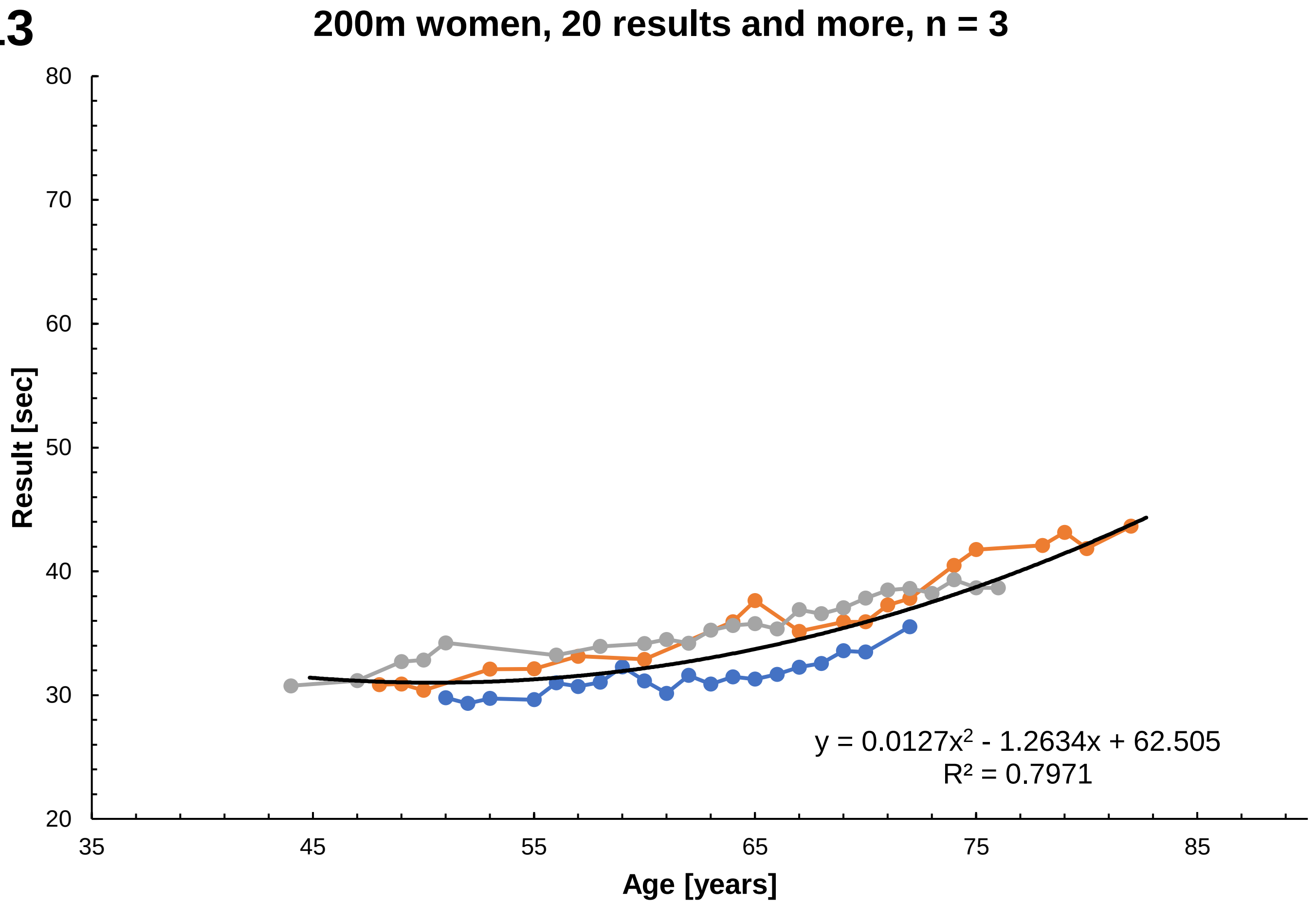

200m women, 20 results and more, n = 3
Result [sec]
Age [years]
y = 0.0127x² - 1.2634x + 62.505
R² = 0.7971

14

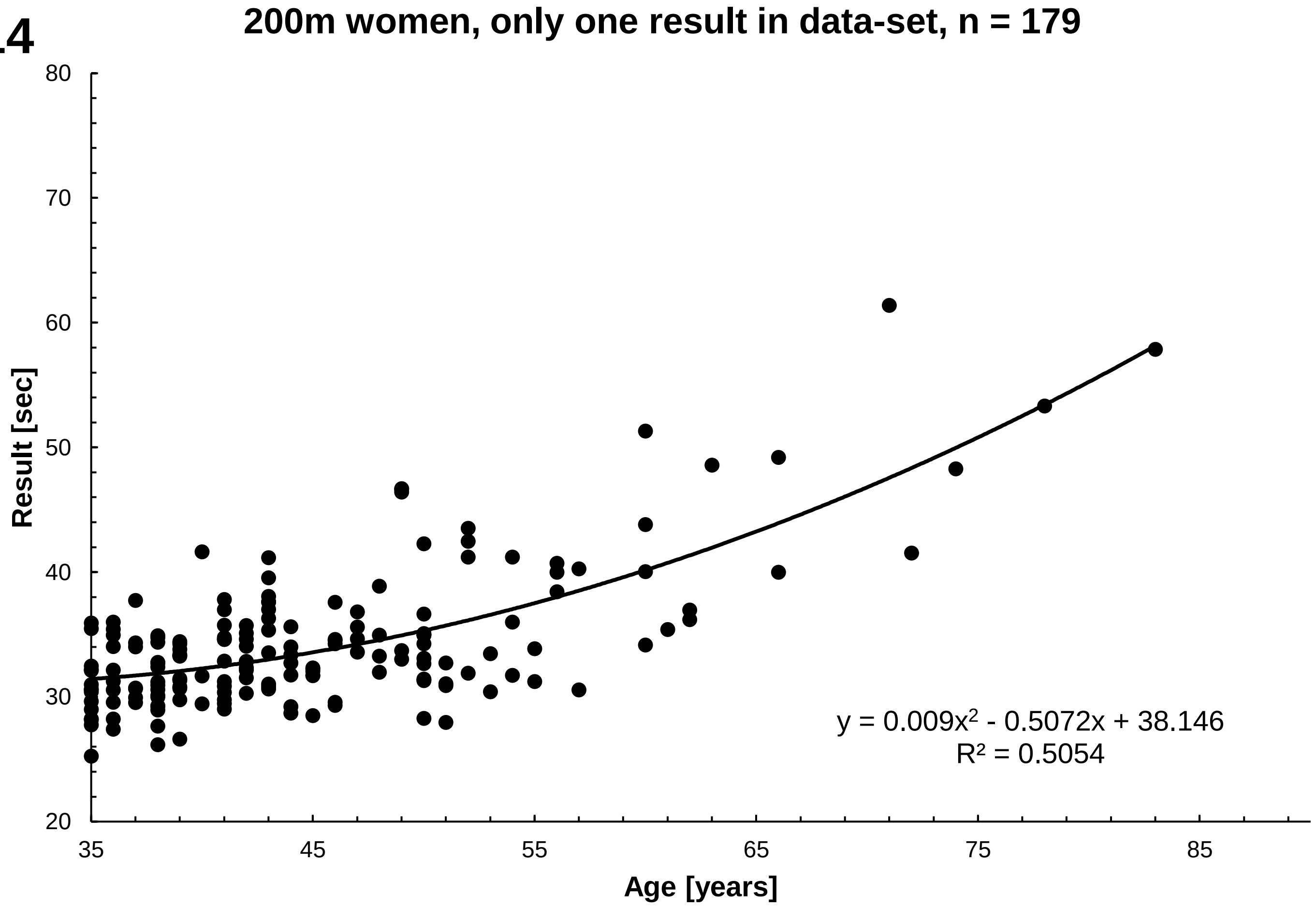

200m women, only one result in data-set, n = 179
Result [sec]
Age [years]
y = 0.009x² - 0.5072x + 38.146
R² = 0.5054

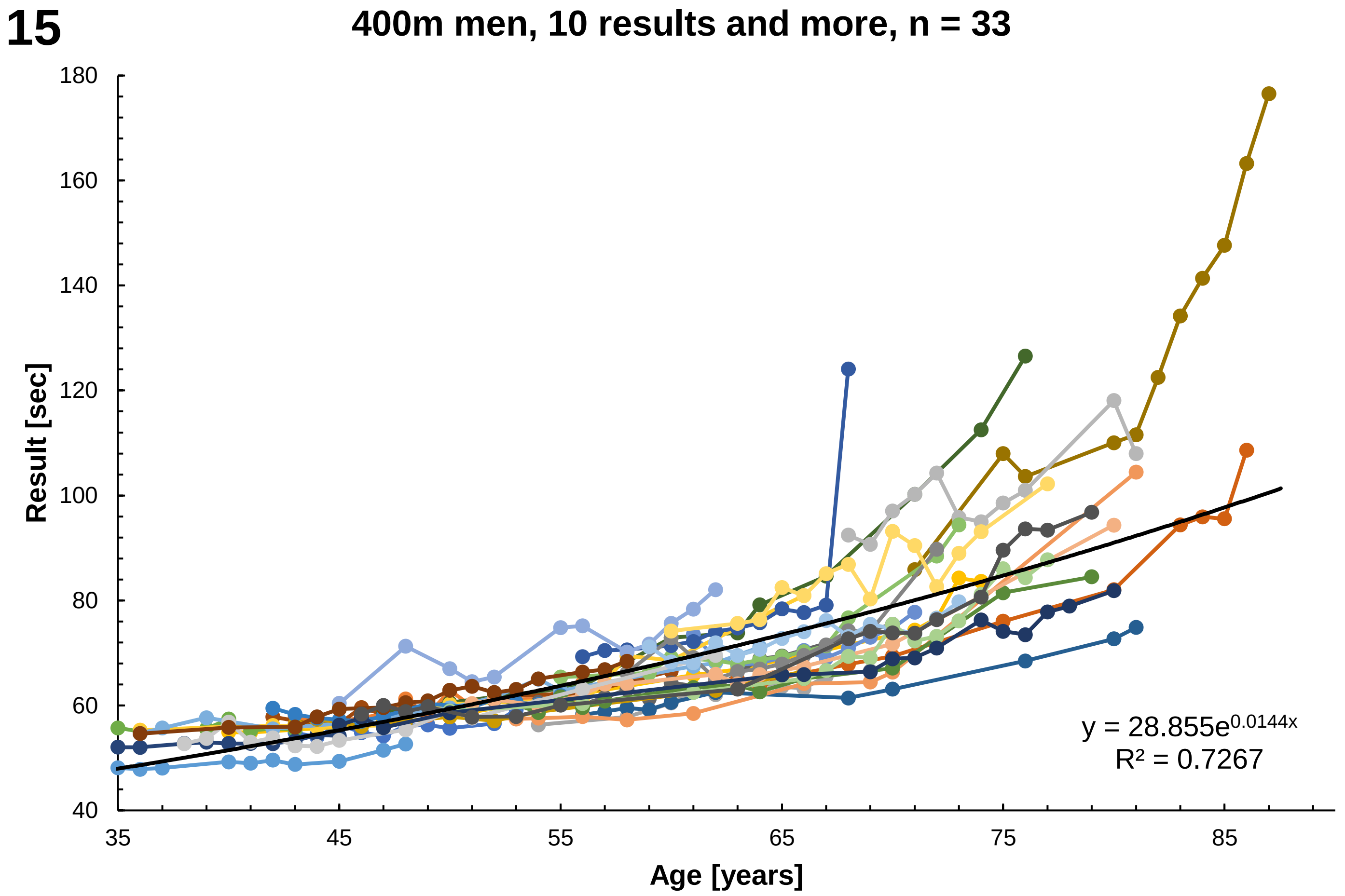
15
400m men, 10 results and more, n = 33
Result [sec]
Age [years]
180
160
140
120
100
80
60
40
35
45
55
65
75
85
y = 28.855e^0.0144x
R² = 0.7267

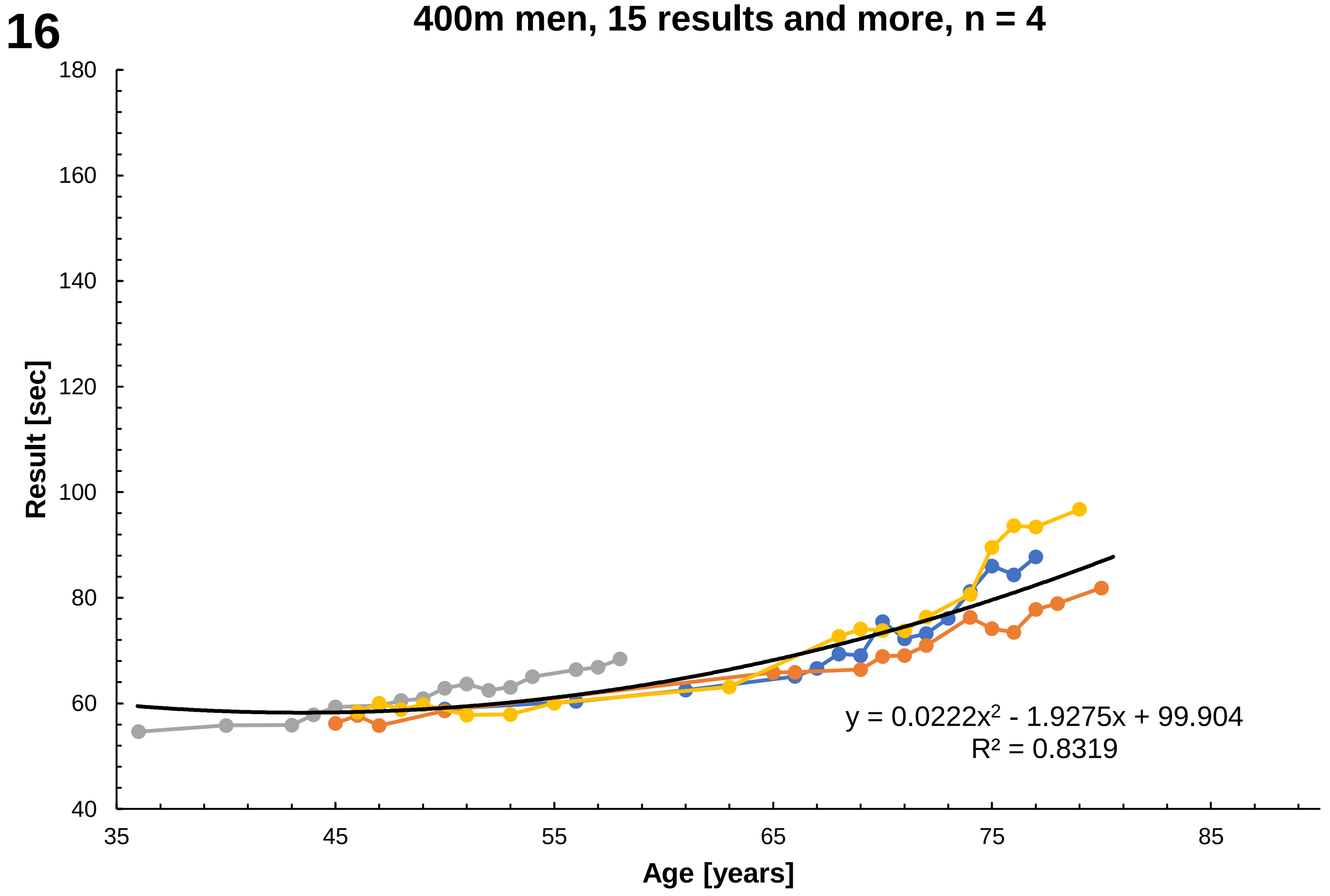
16
400m men, 15 results and more, n = 4
Result [sec]
Age [years]
180
160
140
120
100
80
60
40
35
45
55
65
75
85
y = 0.0222x² - 1.9275x + 99.904
R² = 0.8319

17

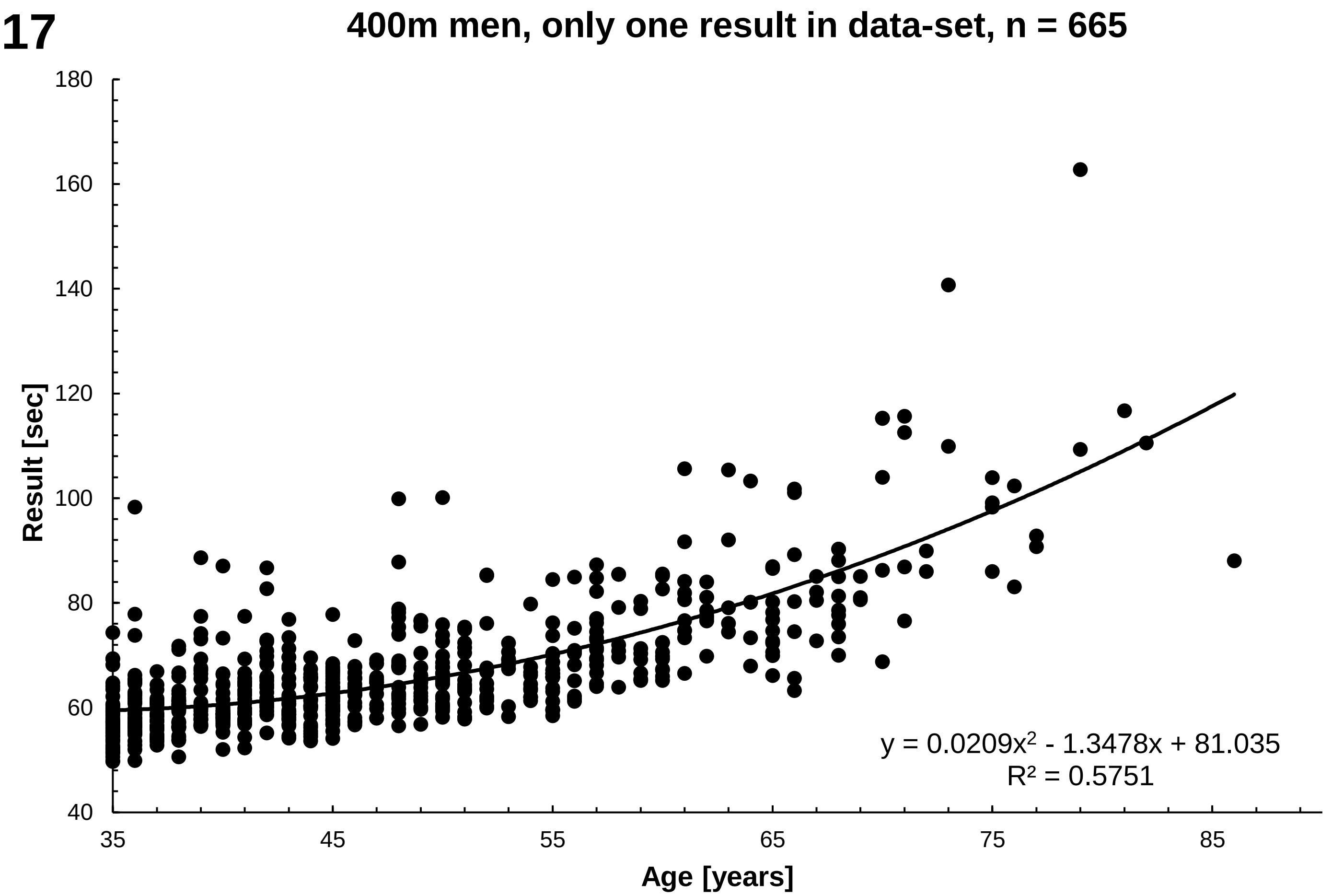
400m men, only one result in data-set, n = 665
Result [sec]
180
160
140
120
100
80
60
40
35
45
55
65
75
85
Age [years]
y = 0.0209x² - 1.3478x + 81.035
R² = 0.5751

18

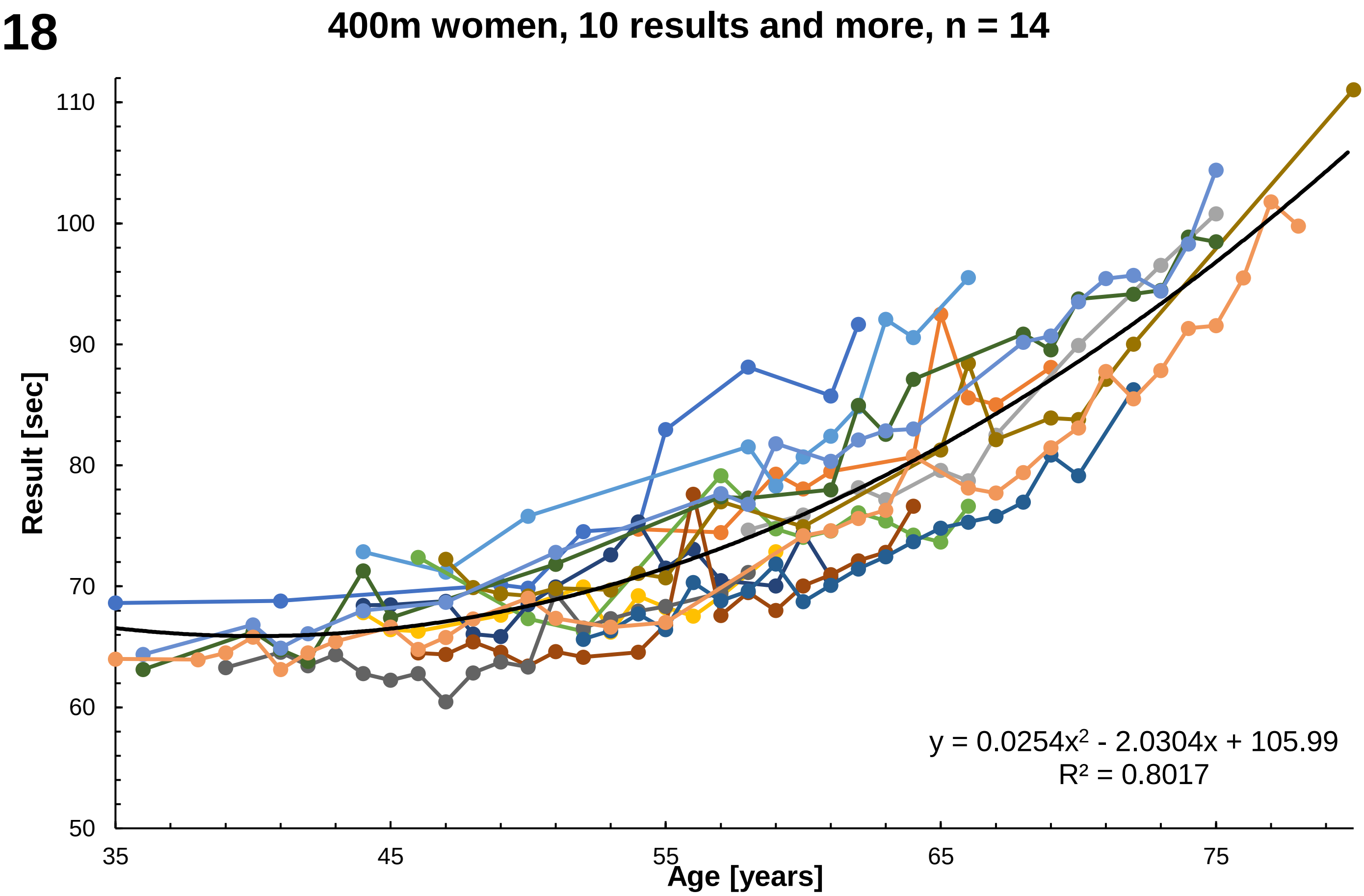
400m women, 10 results and more, n = 14
Result [sec]
110
100
90
80
70
60
50
35
45
55
65
75
Age [years]
y = 0.0254x² - 2.0304x + 105.99
R² = 0.8017

19

400m women, 15 results and more, n = 8

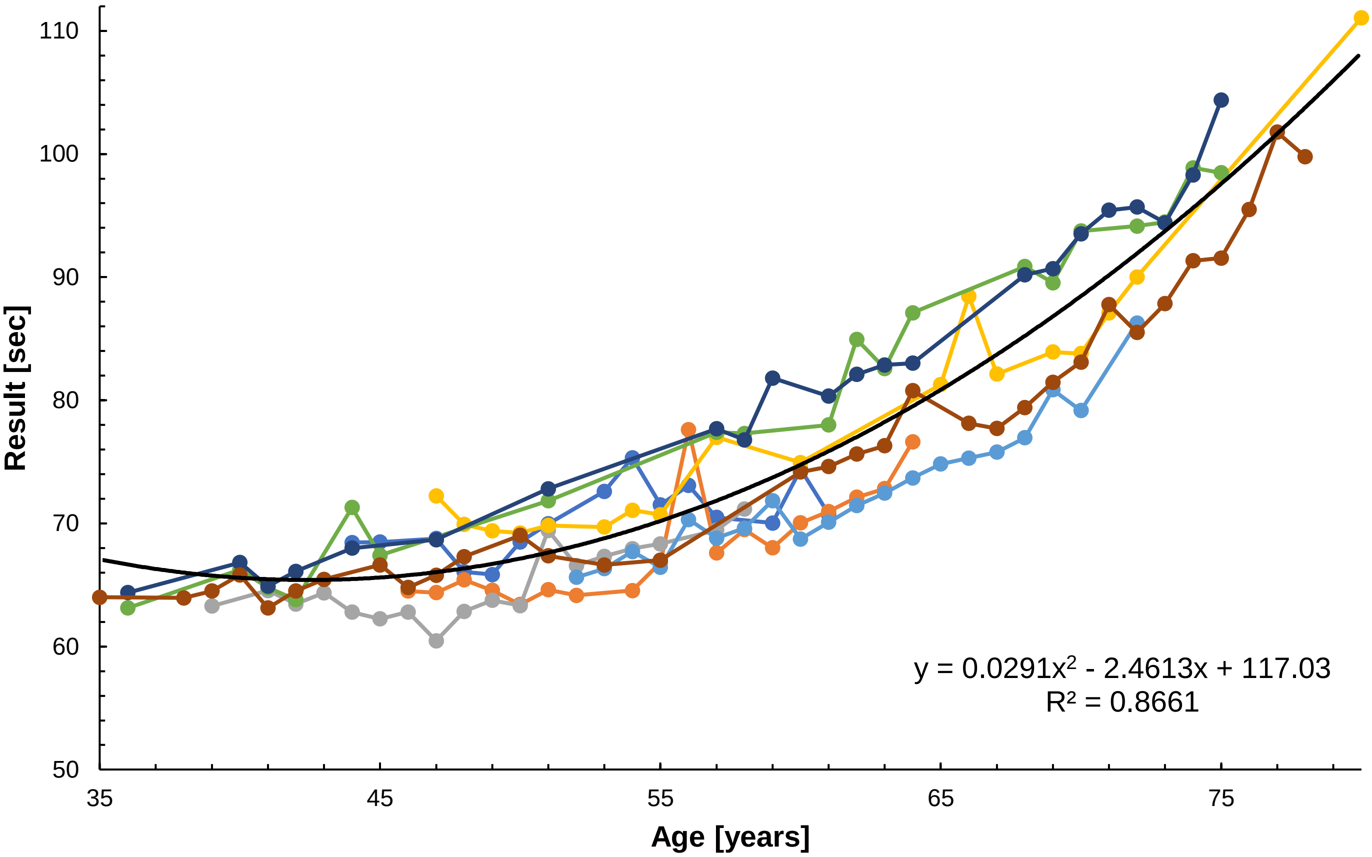

20

400m women, 20 results and more, n = 4

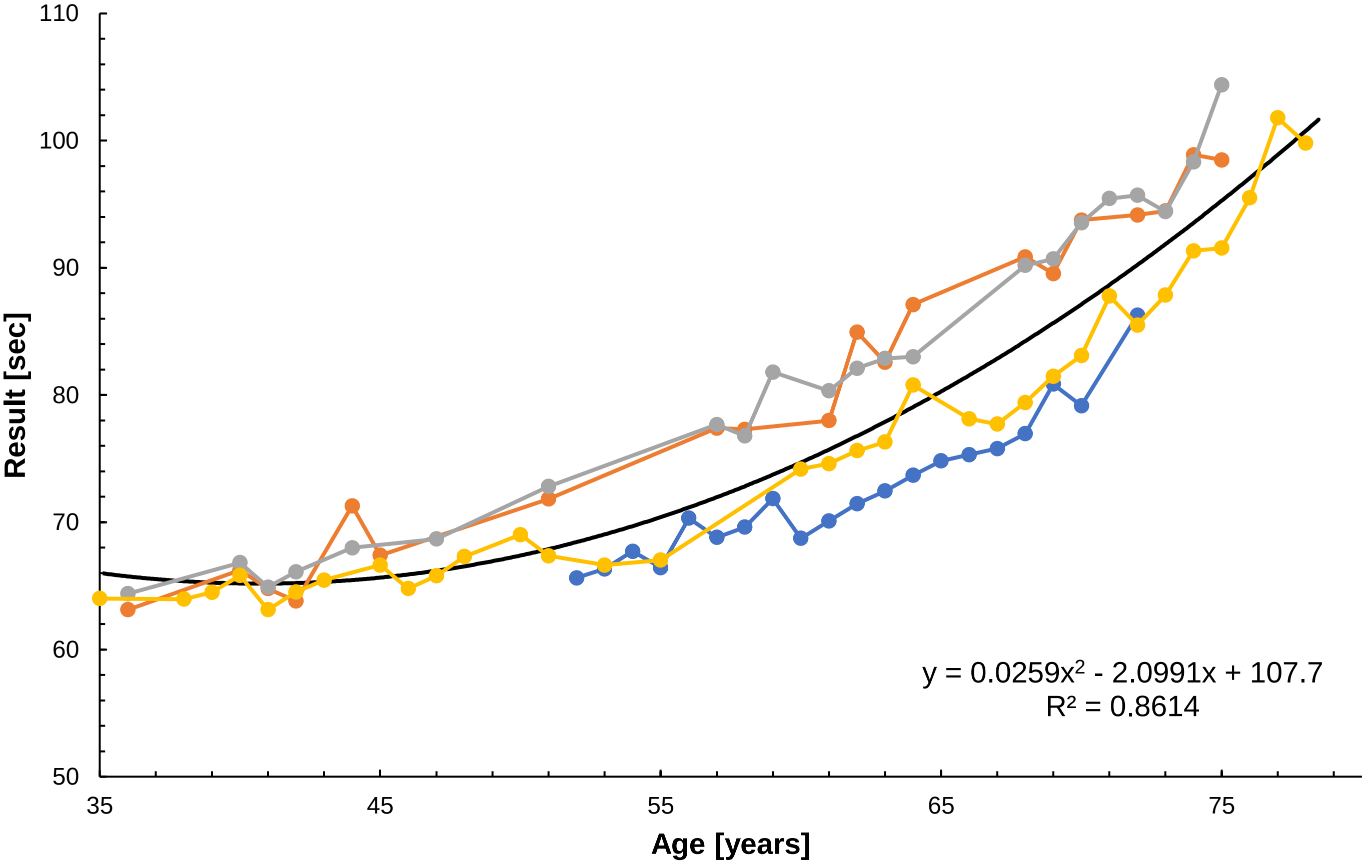

21

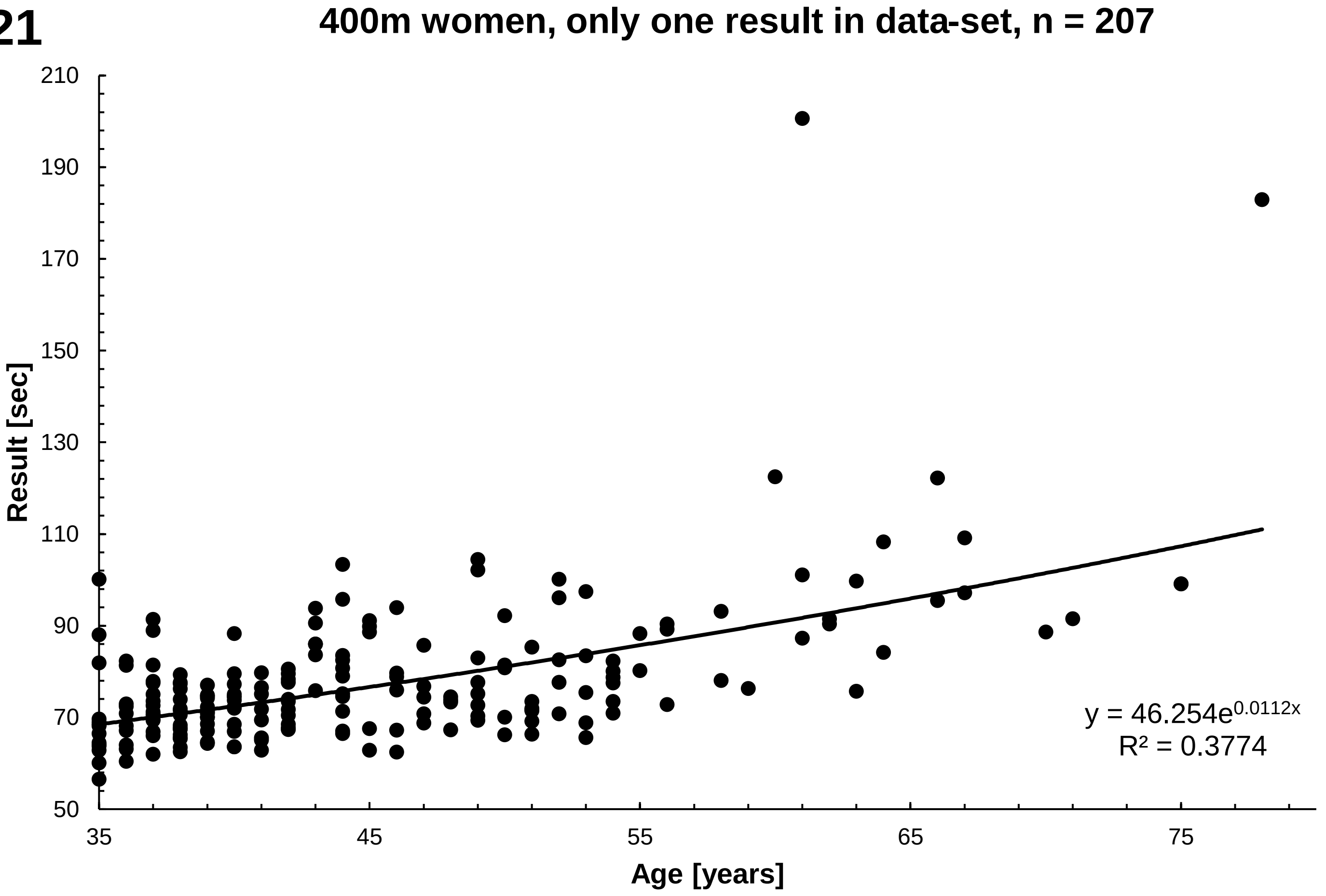
400m women, only one result in data-set, n = 207
Result [sec]
210
190
170
150
130
110
90
70
50
35
45
55
65
75
Age [years]
y = 46.254e$^{0.0112x}$
R² = 0.3774

22

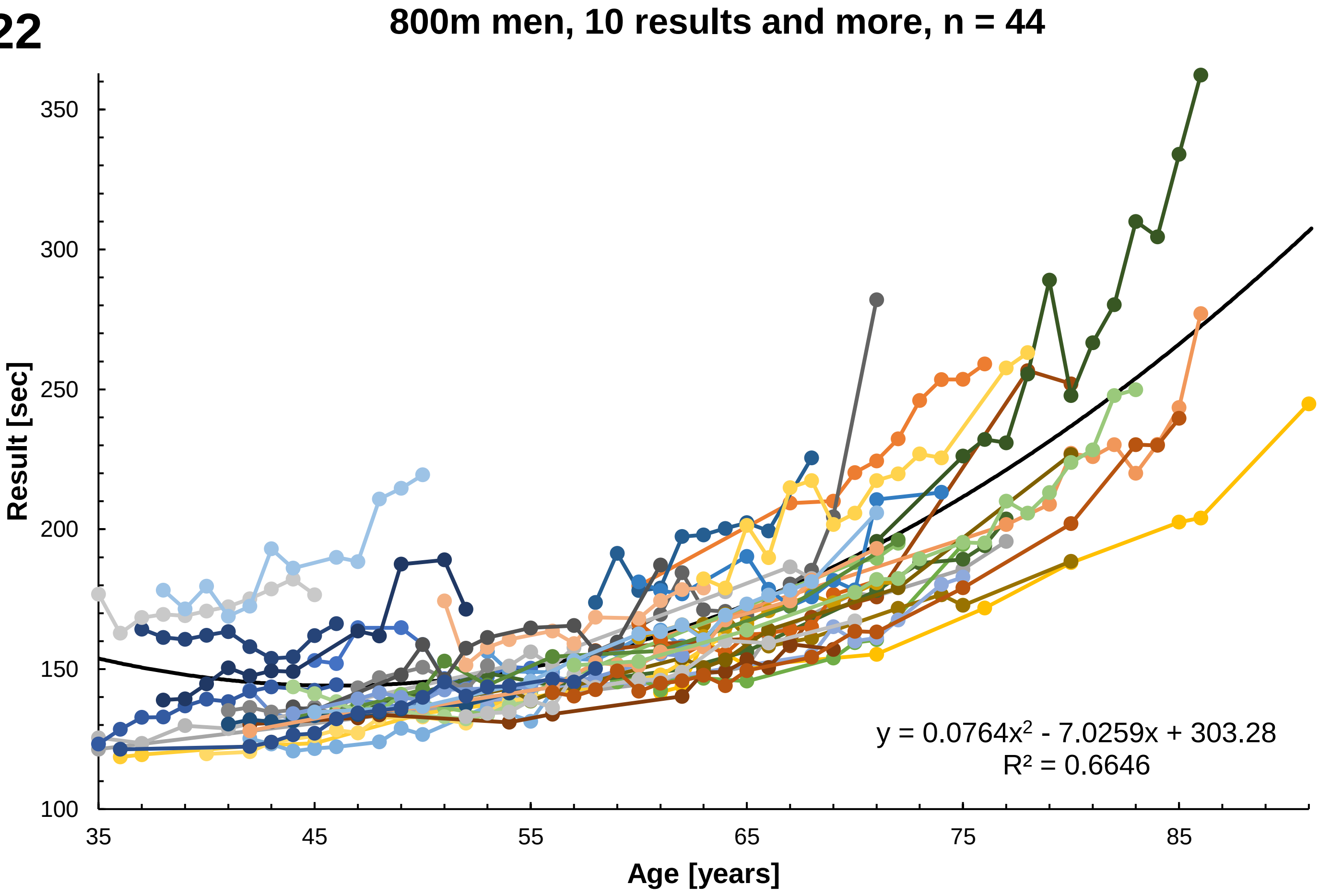
800m men, 10 results and more, n = 44
Result [sec]
350
300
250
200
150
100
35
45
55
65
75
85
Age [years]
y = 0.0764x² - 7.0259x + 303.28
R² = 0.6646

23

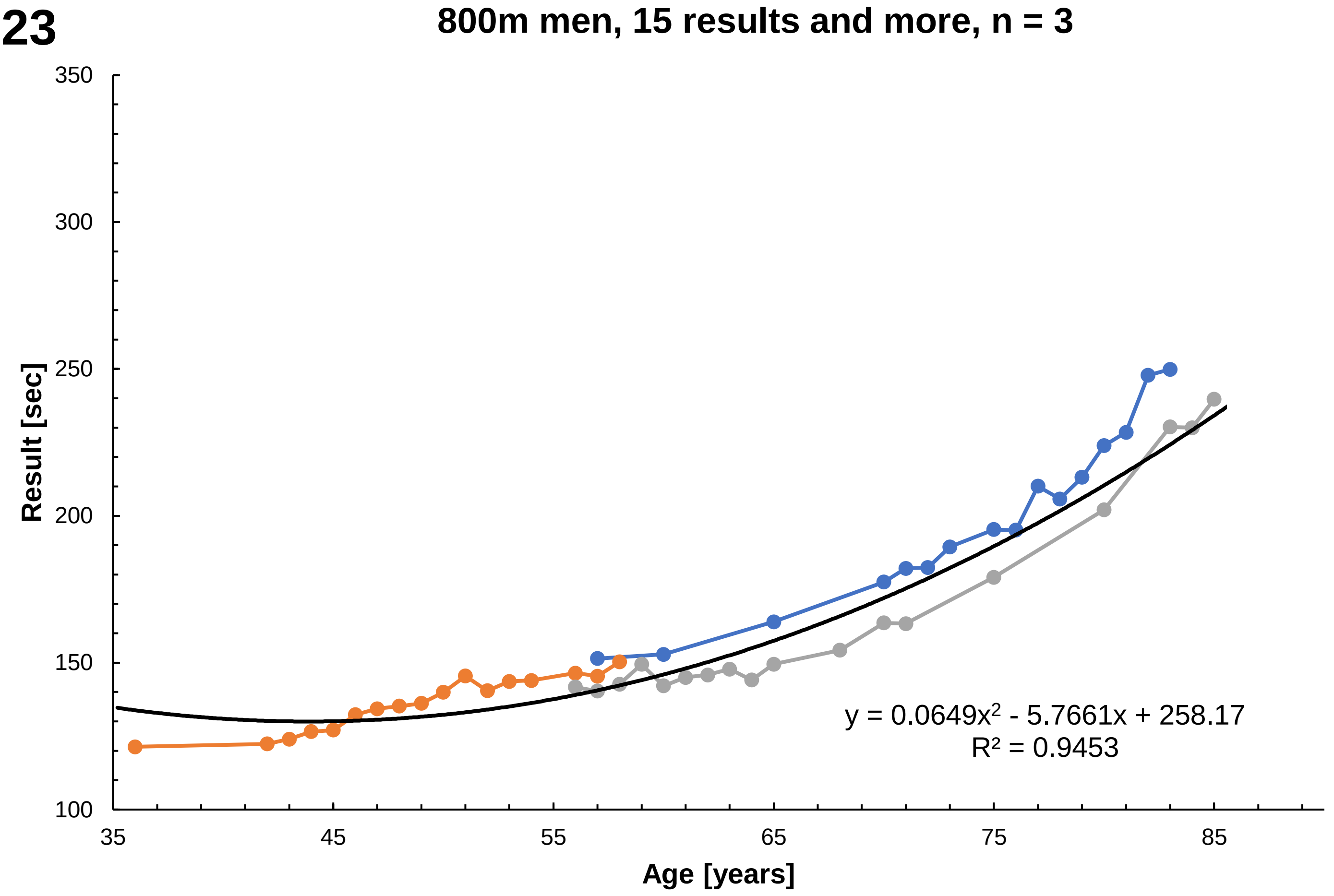
800m men, 15 results and more, n = 3
Result [sec]
Age [years]
350
300
250
200
150
100
35
45
55
65
75
85
y = 0.0649x² - 5.7661x + 258.17
R² = 0.9453

24

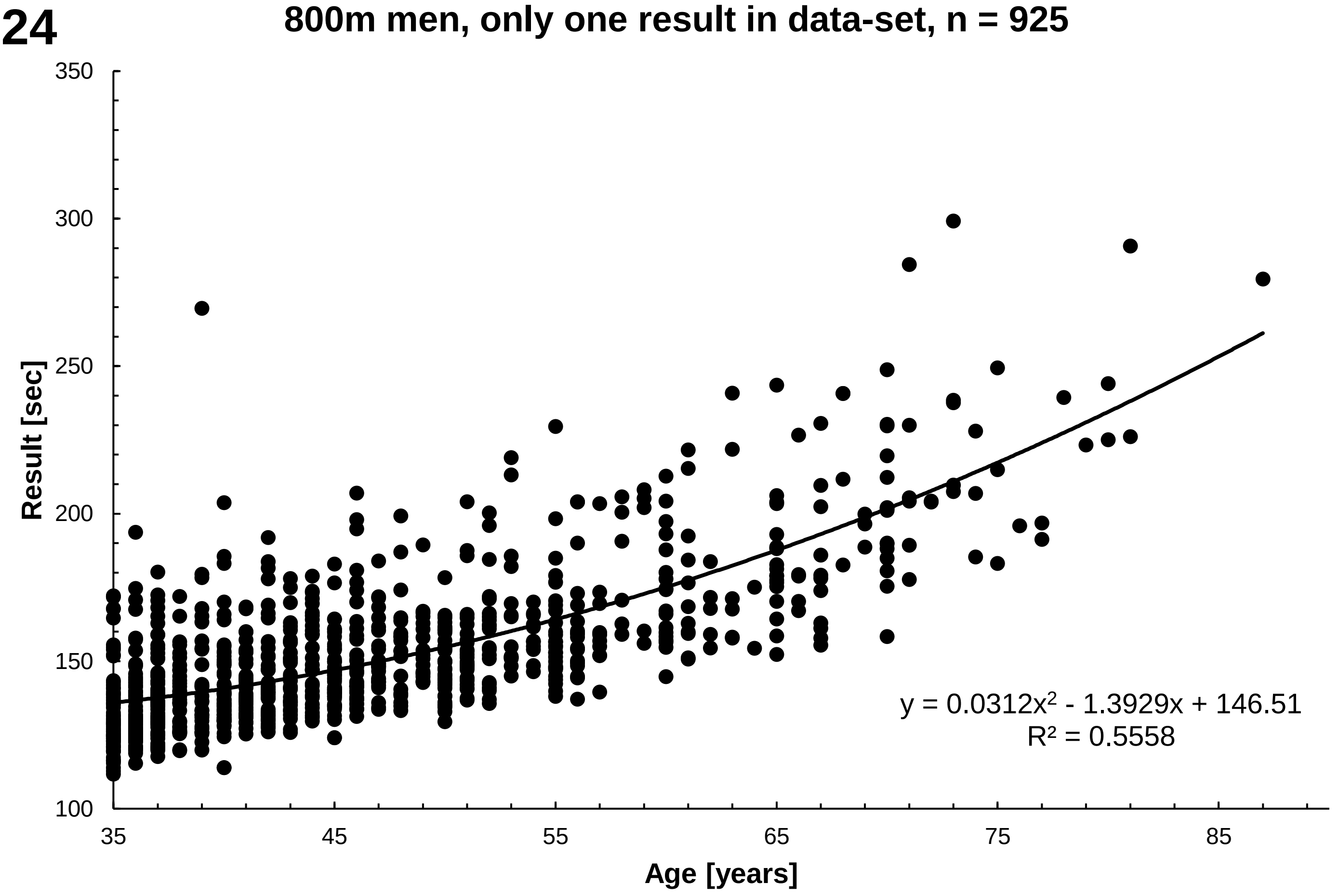
800m men, only one result in data-set, n = 925
Result [sec]
Age [years]
350
300
250
200
150
100
35
45
55
65
75
85
y = 0.0312x² - 1.3929x + 146.51
R² = 0.5558

25

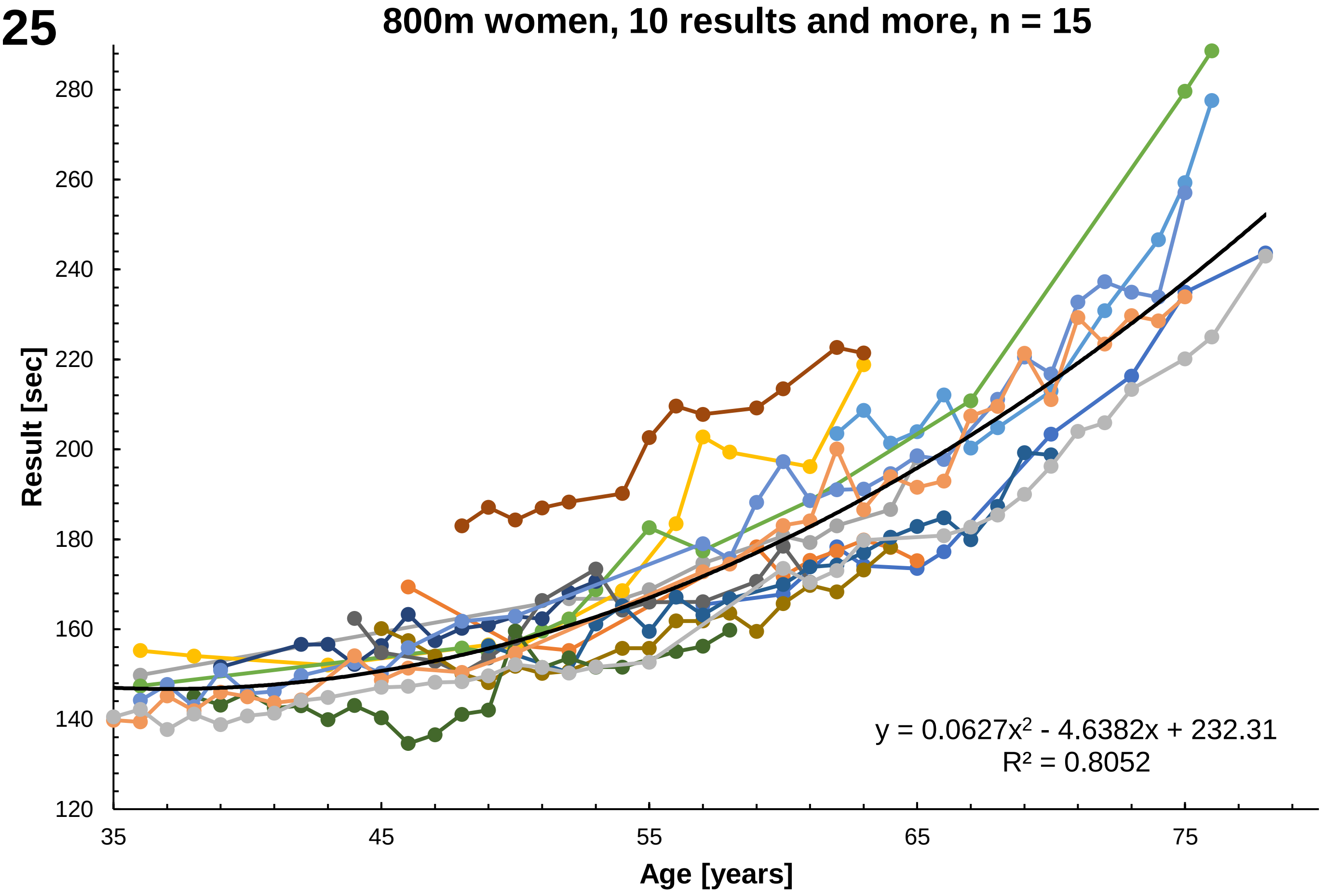
800m women, 10 results and more, n = 15
Result [sec]
280
260
240
220
200
180
160
140
120
35
45
55
65
75
Age [years]
y = 0.0627x² - 4.6382x + 232.31
R² = 0.8052

26

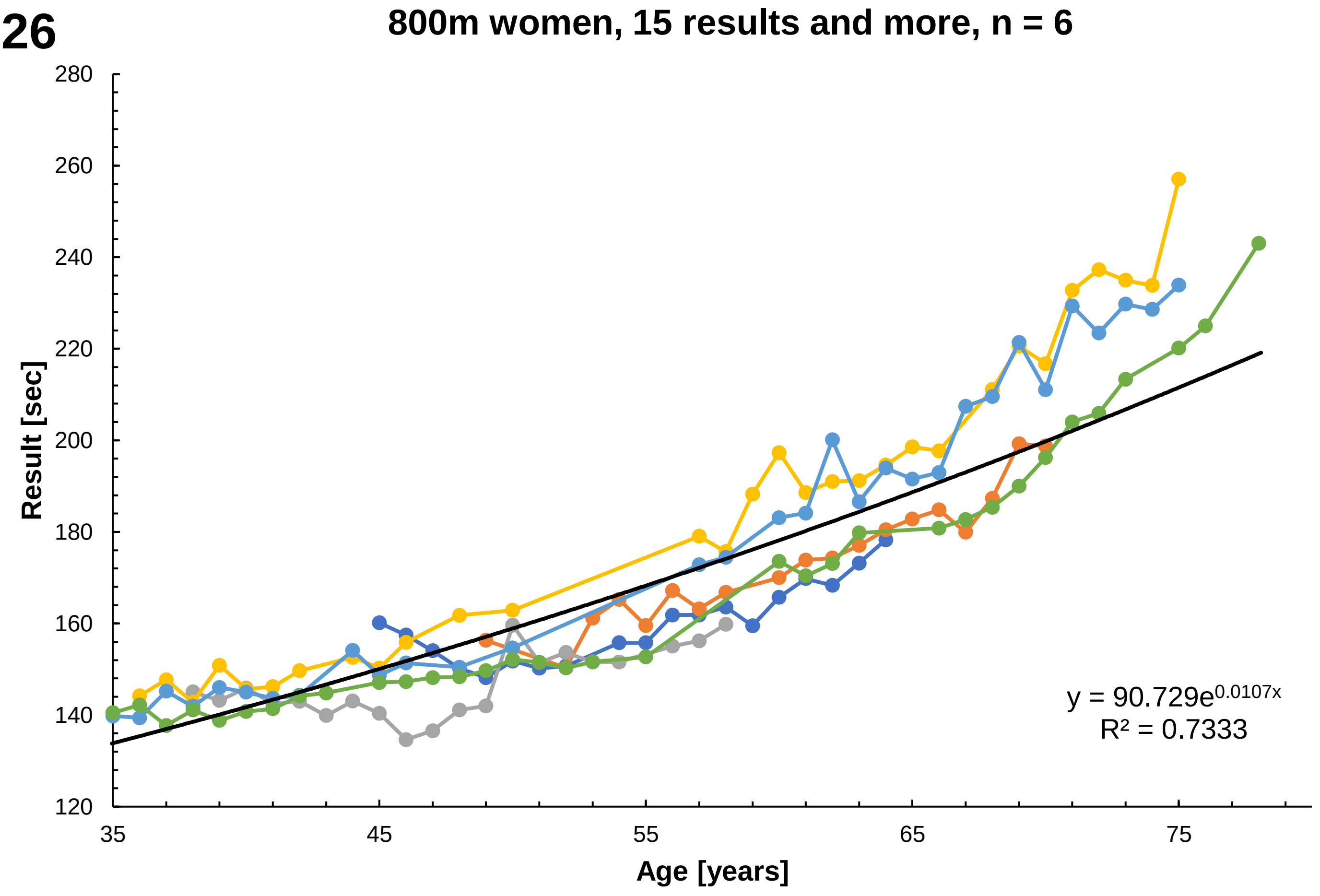
800m women, 15 results and more, n = 6
Result [sec]
280
260
240
220
200
180
160
140
120
35
45
55
65
75
Age [years]
y = 90.729e^0.0107x
R² = 0.7333

27

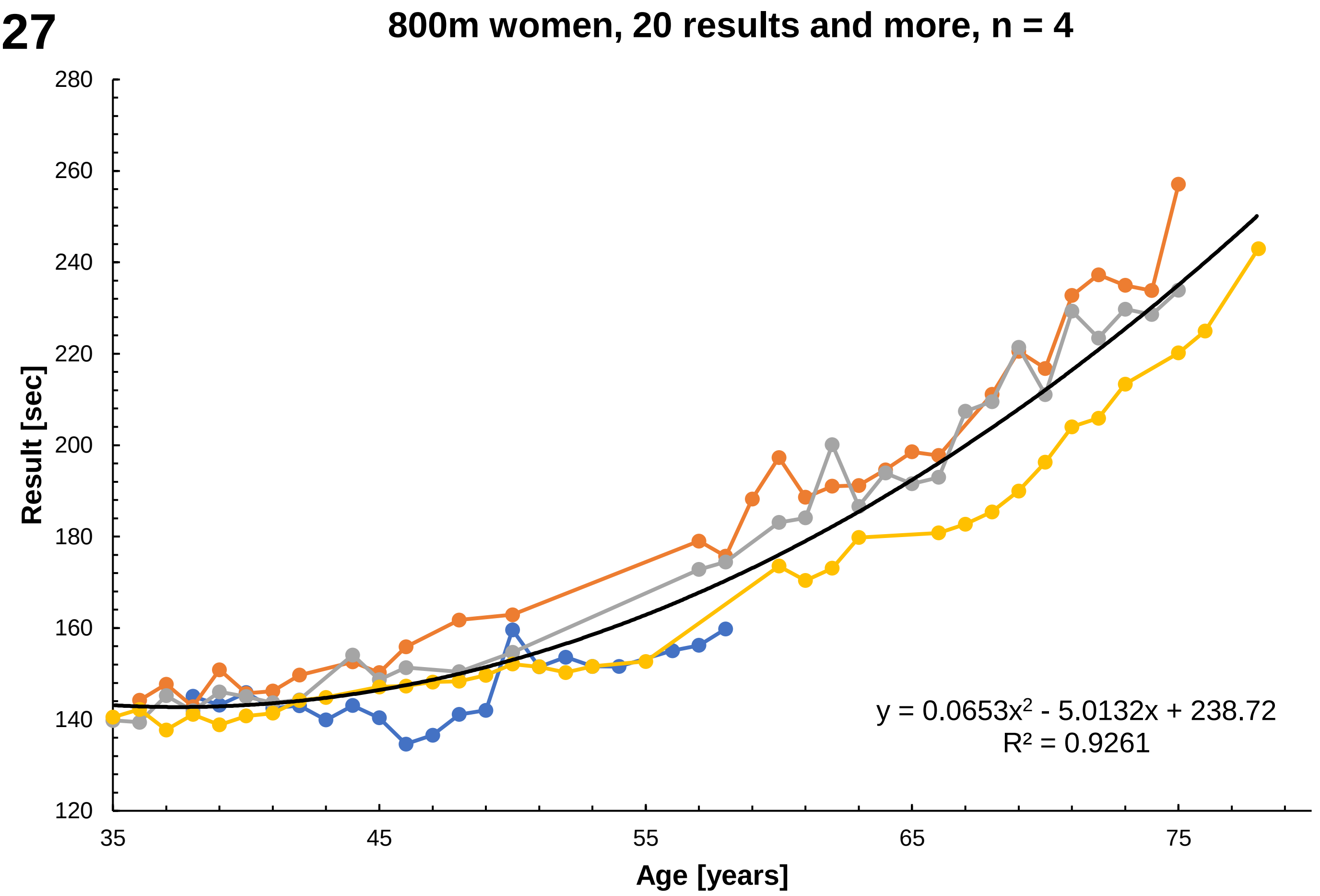
800m women, 20 results and more, n = 4
Result [sec]
280
260
240
220
200
180
160
140
120
35
45
55
65
75
Age [years]
y = 0.0653x² - 5.0132x + 238.72
R² = 0.9261

28

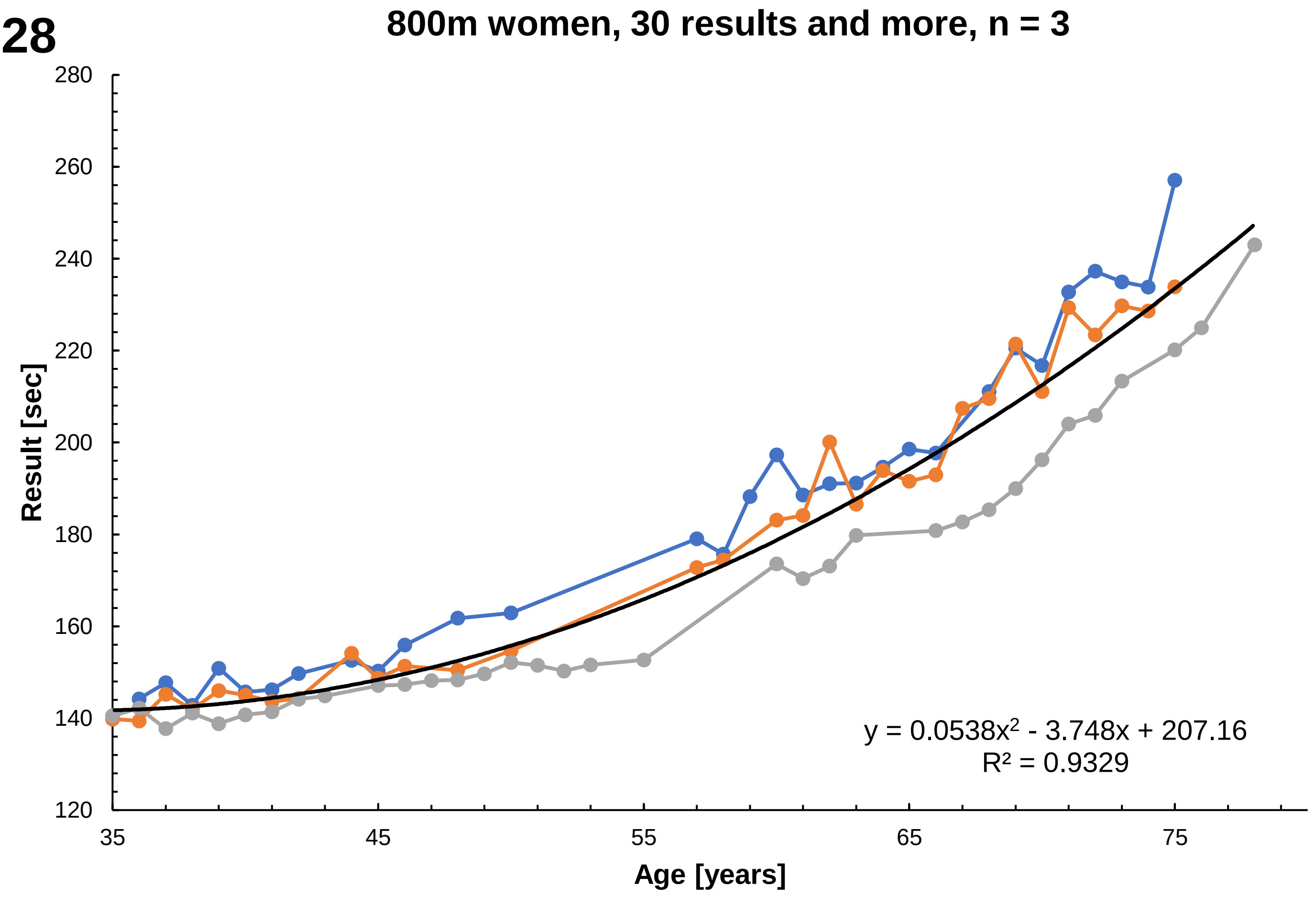
800m women, 30 results and more, n = 3
Result [sec]
280
260
240
220
200
180
160
140
120
35
45
55
65
75
Age [years]
y = 0.0538x² - 3.748x + 207.16
R² = 0.9329

29

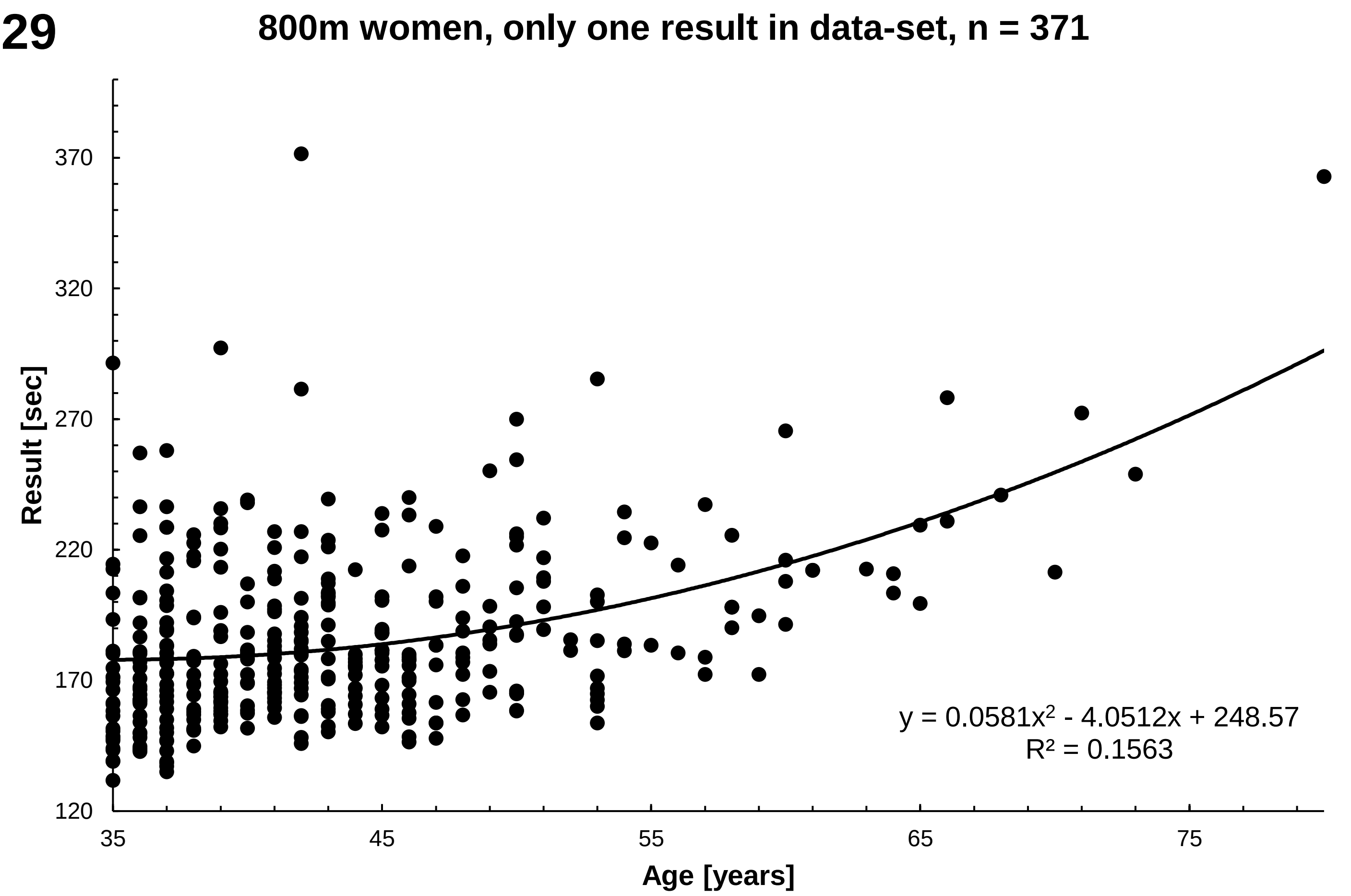
800m women, only one result in data-set, n = 371
Result [sec]
370
320
270
220
170
120
35
45
55
65
75
Age [years]
y = 0.0581x² - 4.0512x + 248.57
R² = 0.1563

30

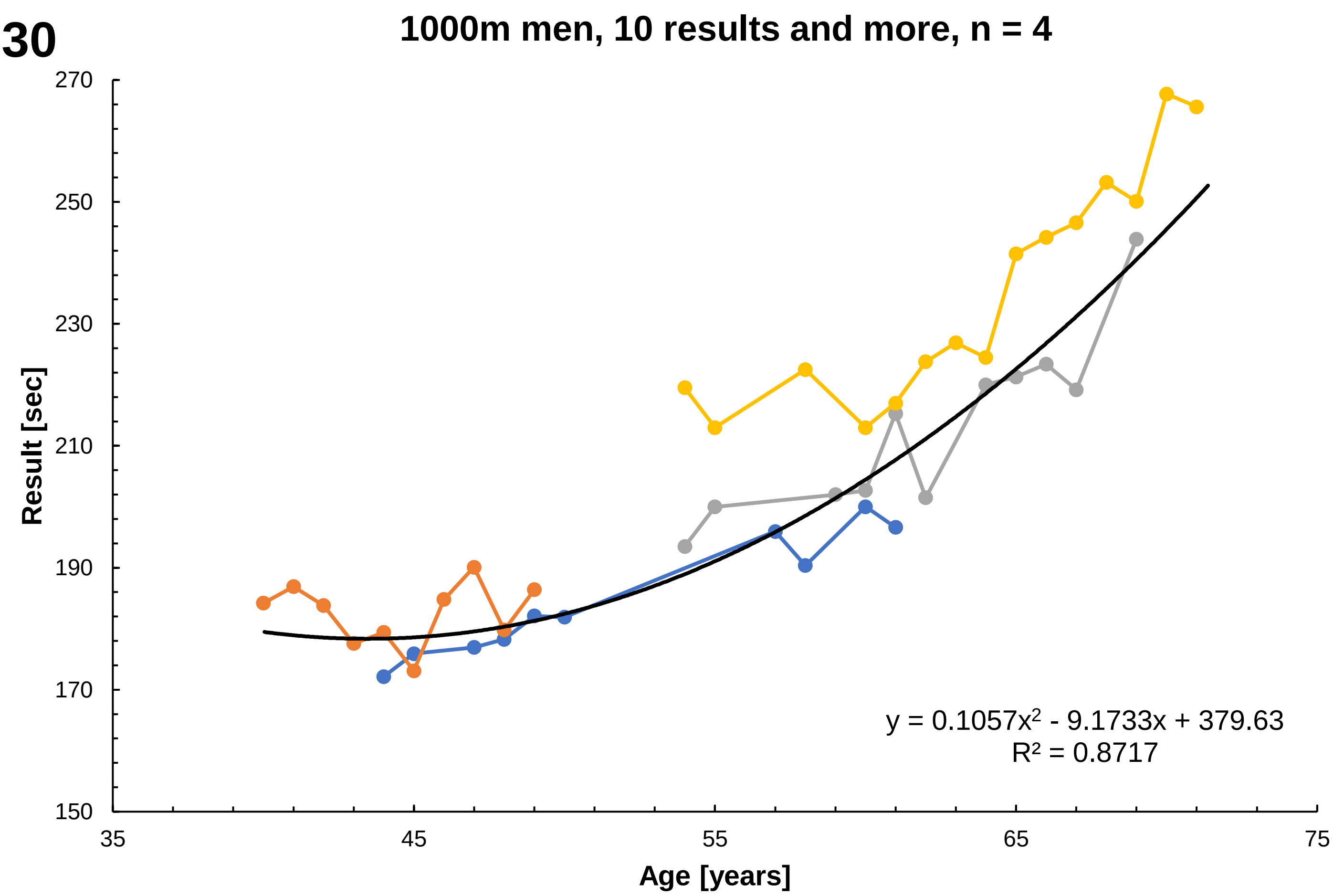
1000m men, 10 results and more, n = 4
Result [sec]
270
250
230
210
190
170
150
35
45
55
65
75
Age [years]
y = 0.1057x² - 9.1733x + 379.63
R² = 0.8717

31

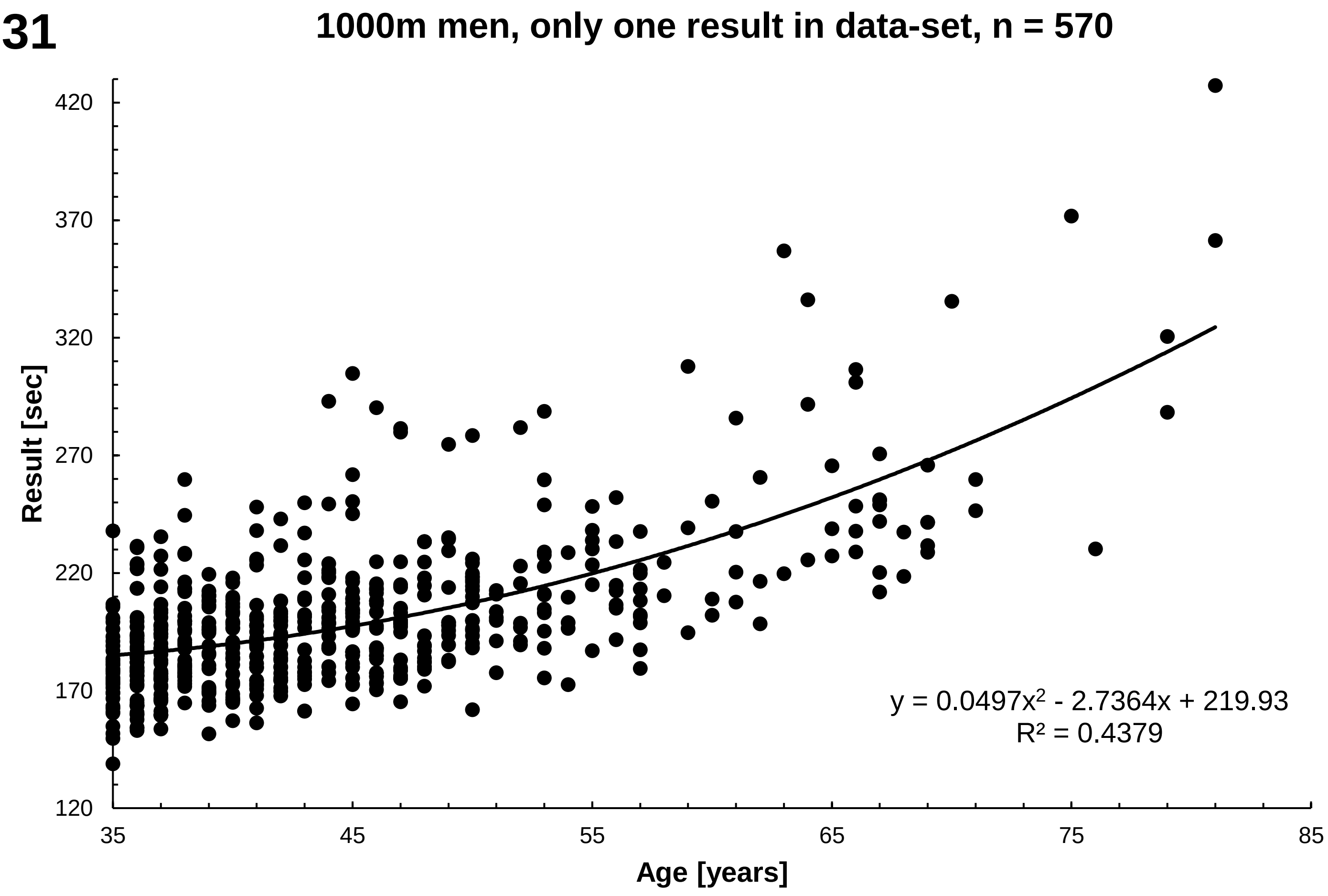
1000m men, only one result in data-set, n = 570
Result [sec]
Age [years]
420
370
320
270
220
170
120
35
45
55
65
75
85
y = 0.0497x² - 2.7364x + 219.93
R² = 0.4379

32

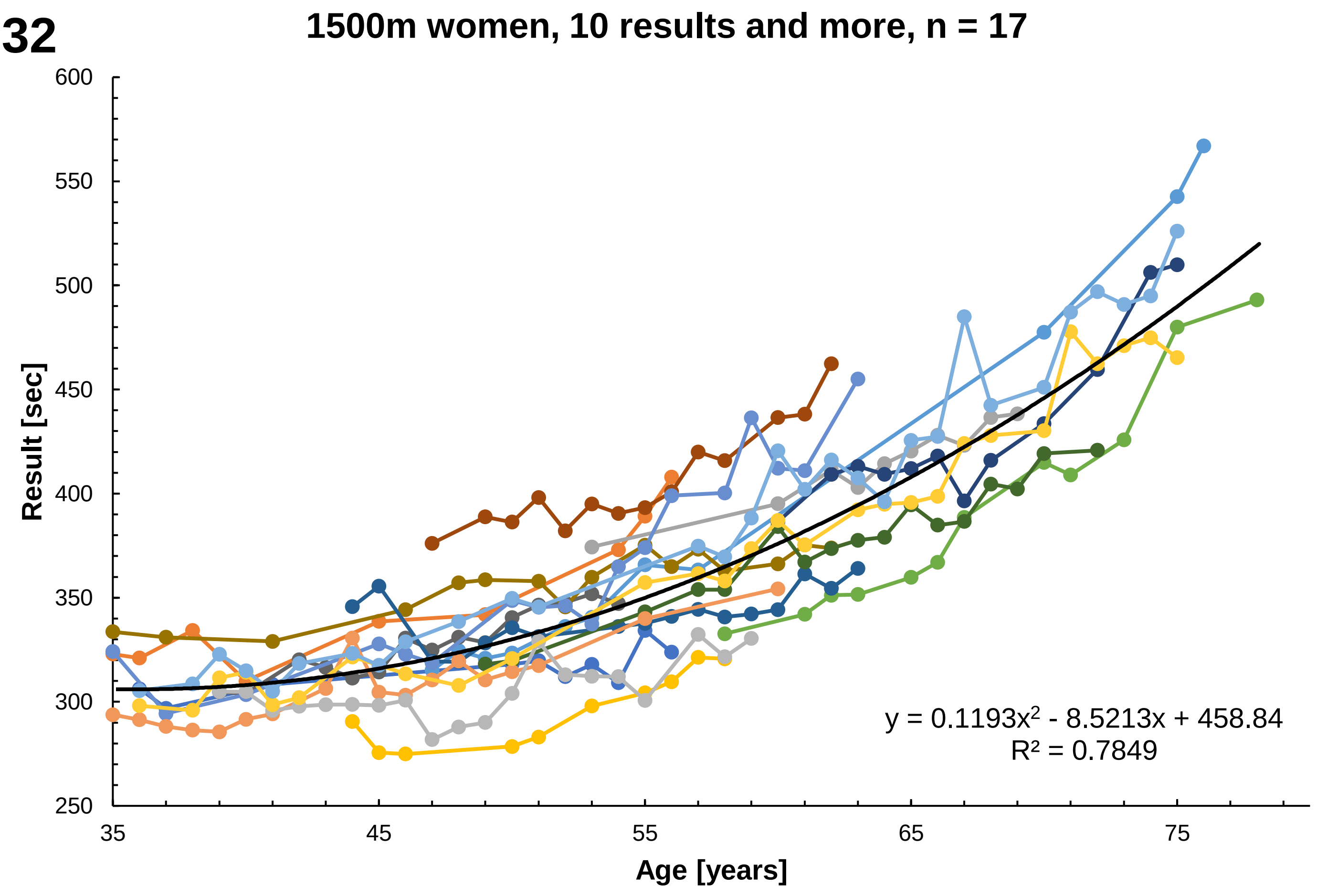
1500m women, 10 results and more, n = 17
Result [sec]
Age [years]
600
550
500
450
400
350
300
250
35
45
55
65
75
y = 0.1193x² - 8.5213x + 458.84
R² = 0.7849

33 **1500m women, only one result in data-set, n = 417**

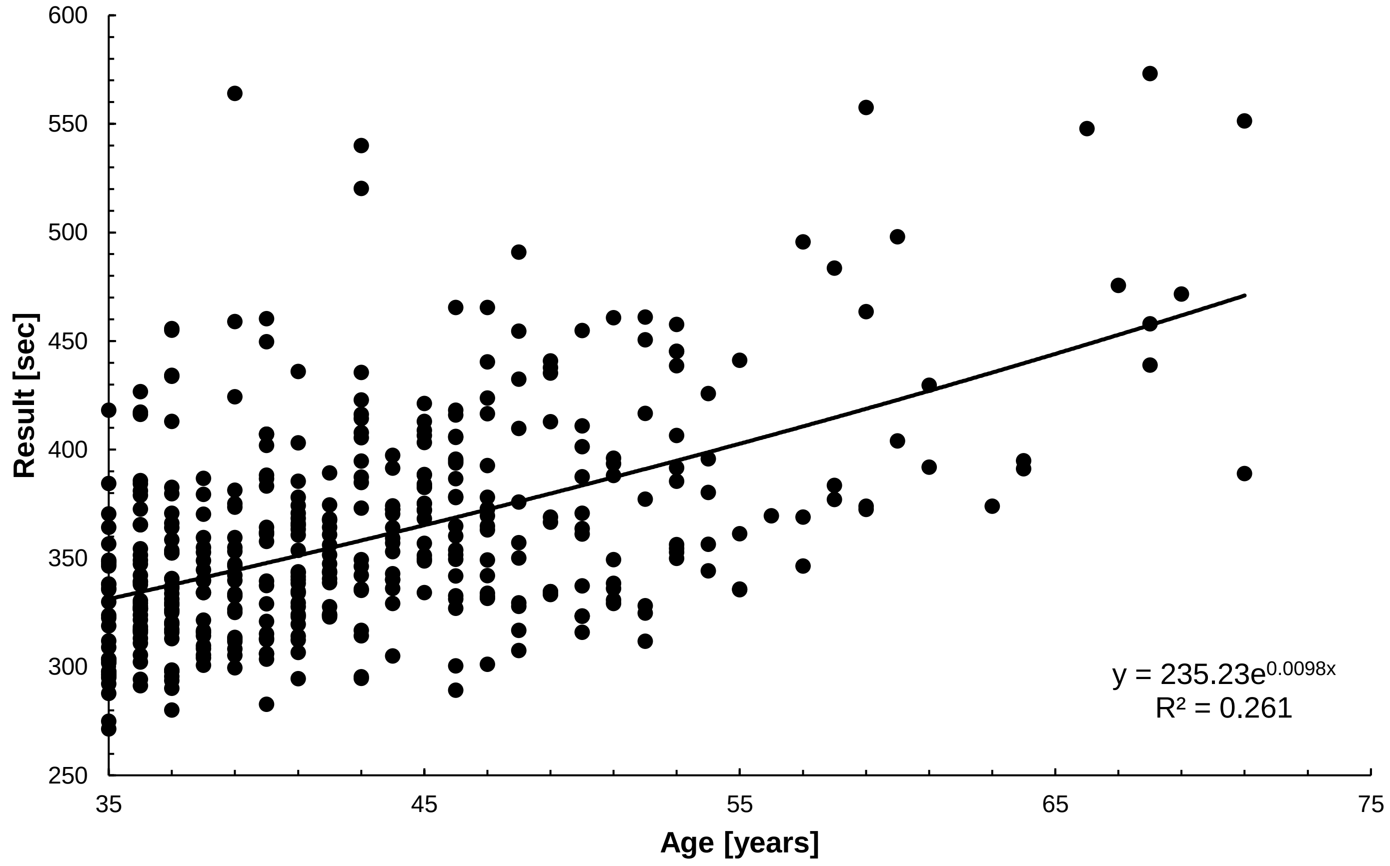

34 **3000m men, 10 results and more, n = 9**

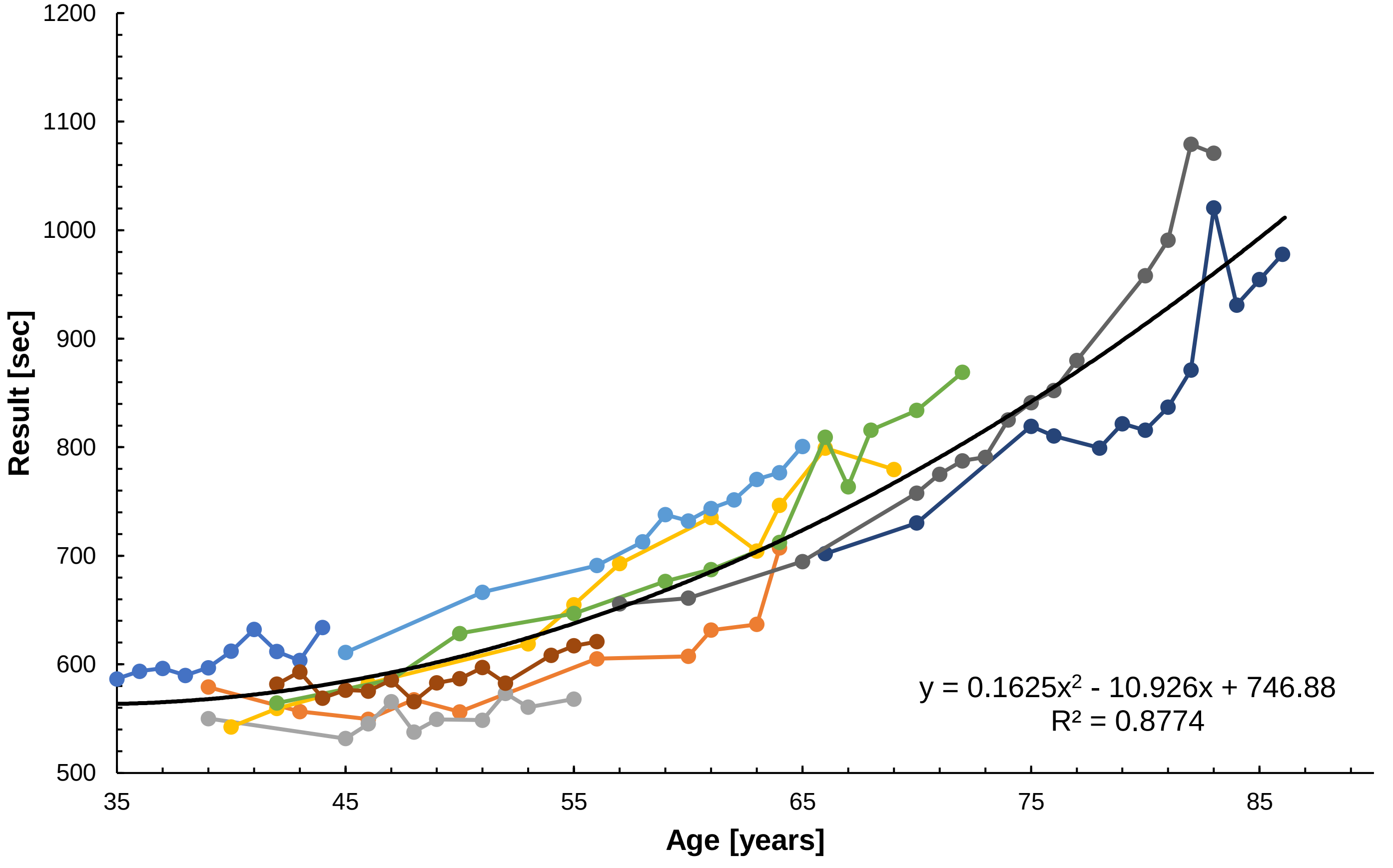

35

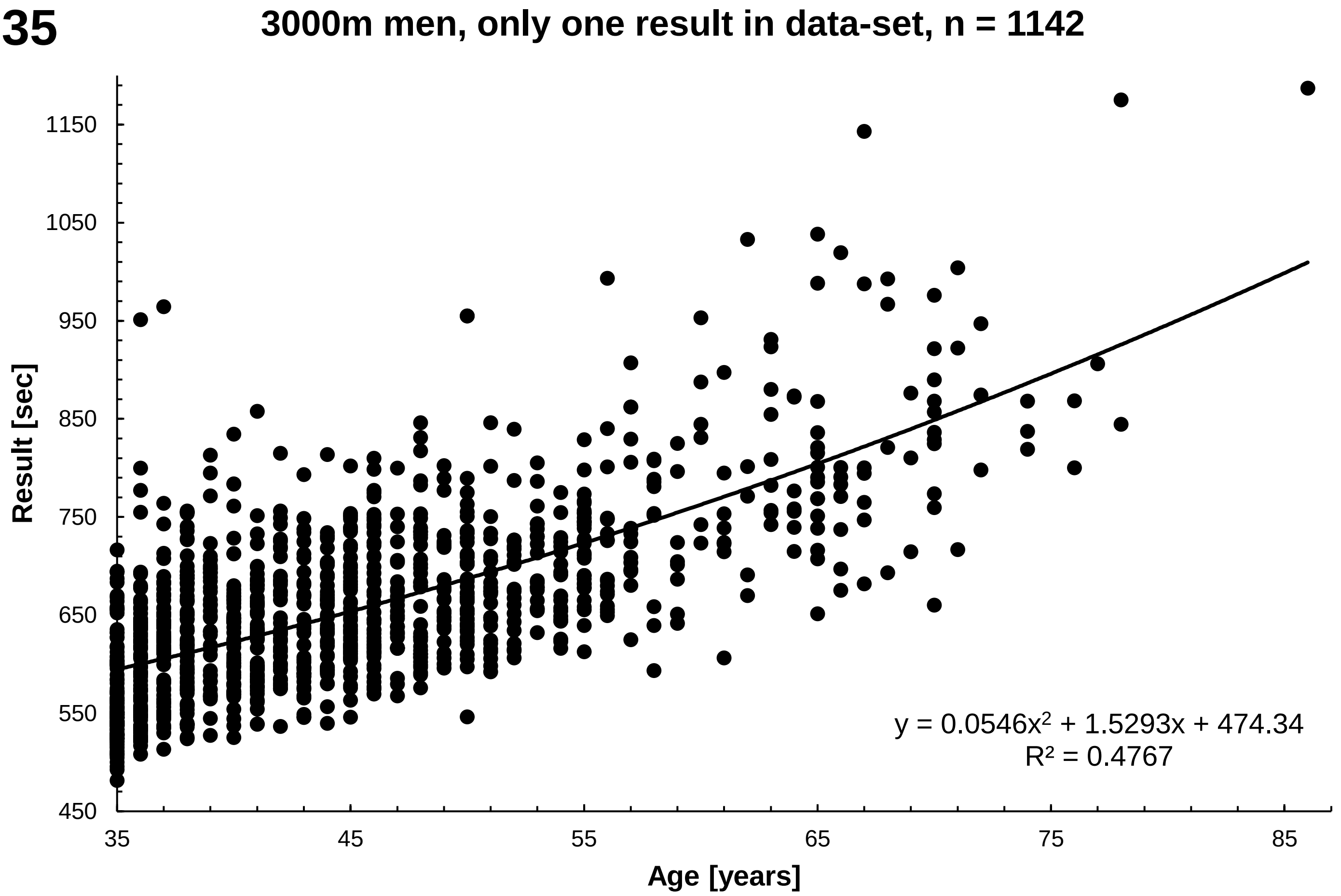
3000m men, only one result in data-set, n = 1142
Result [sec]
Age [years]
y = 0.0546x2 + 1.5293x + 474.34
R² = 0.4767
1150
1050
950
850
750
650
550
450
35
45
55
65
75
85

36

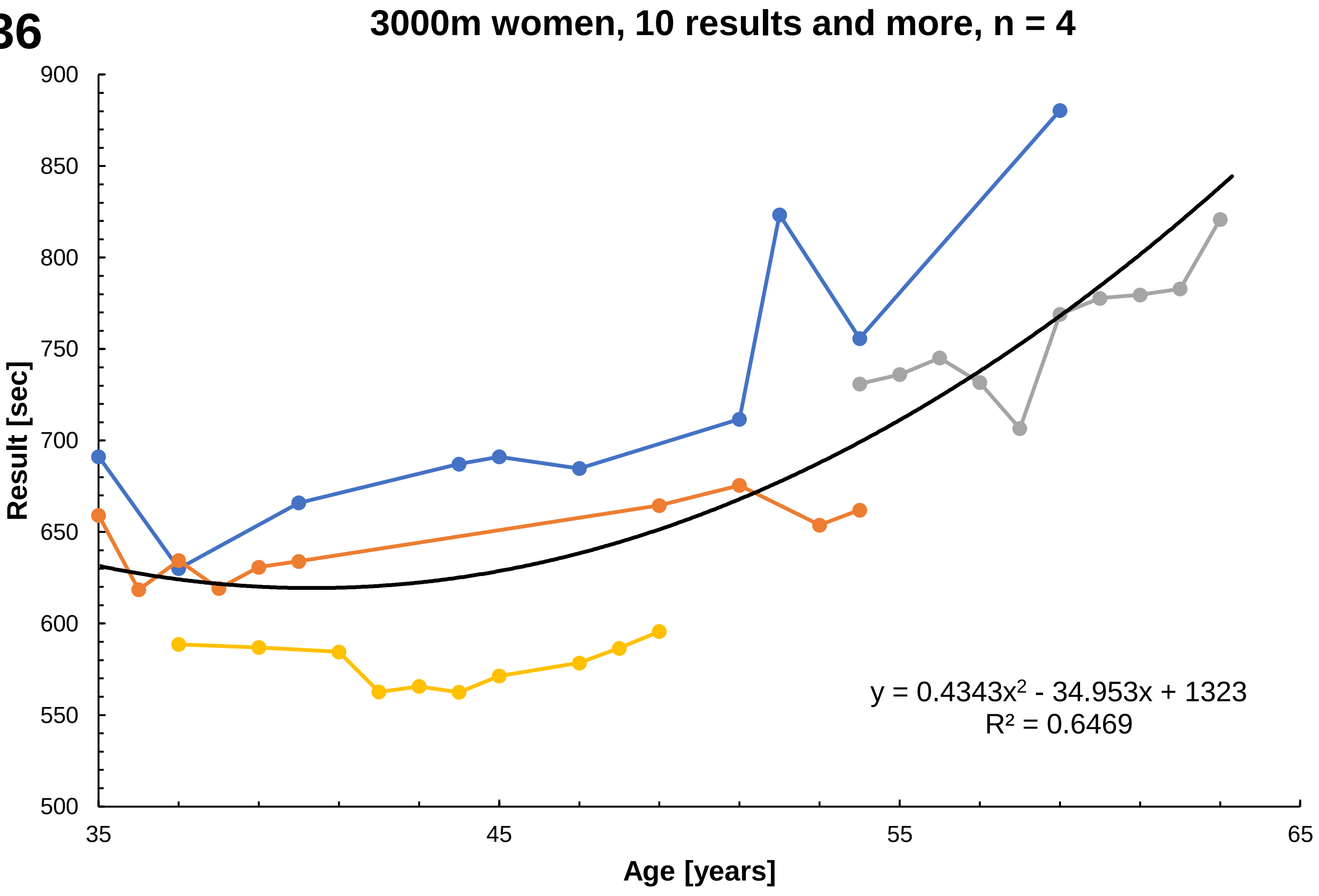
3000m women, 10 results and more, n = 4
Result [sec]
Age [years]
y = 0.4343x2 - 34.953x + 1323
R² = 0.6469
900
850
800
750
700
650
600
550
500
35
45
55
65

37

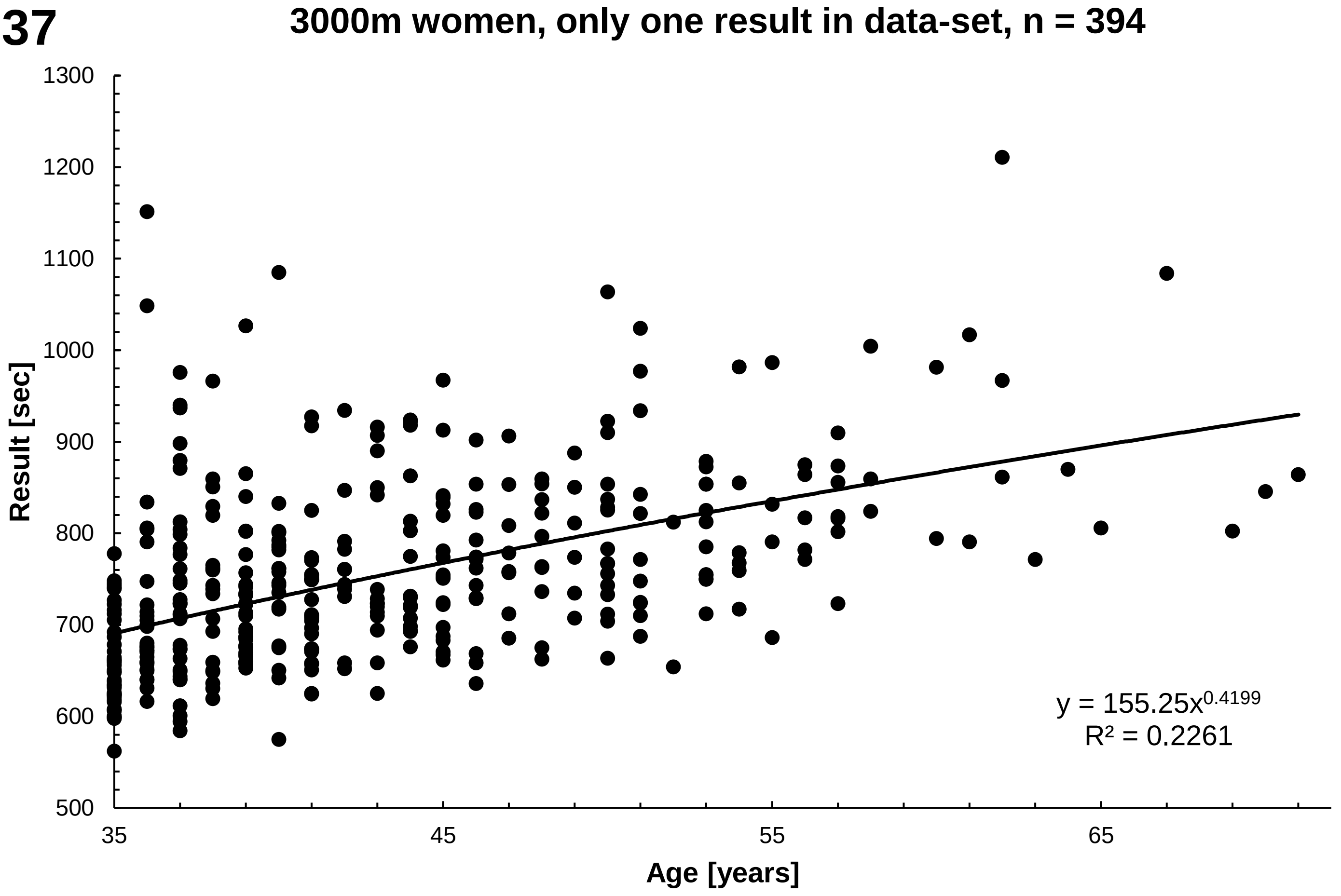
3000m women, only one result in data-set, n = 394
Result [sec]
1300
1200
1100
1000
900
800
700
600
500
35
45
55
65
Age [years]
y = 155.25x^0.4199
R² = 0.2261

38

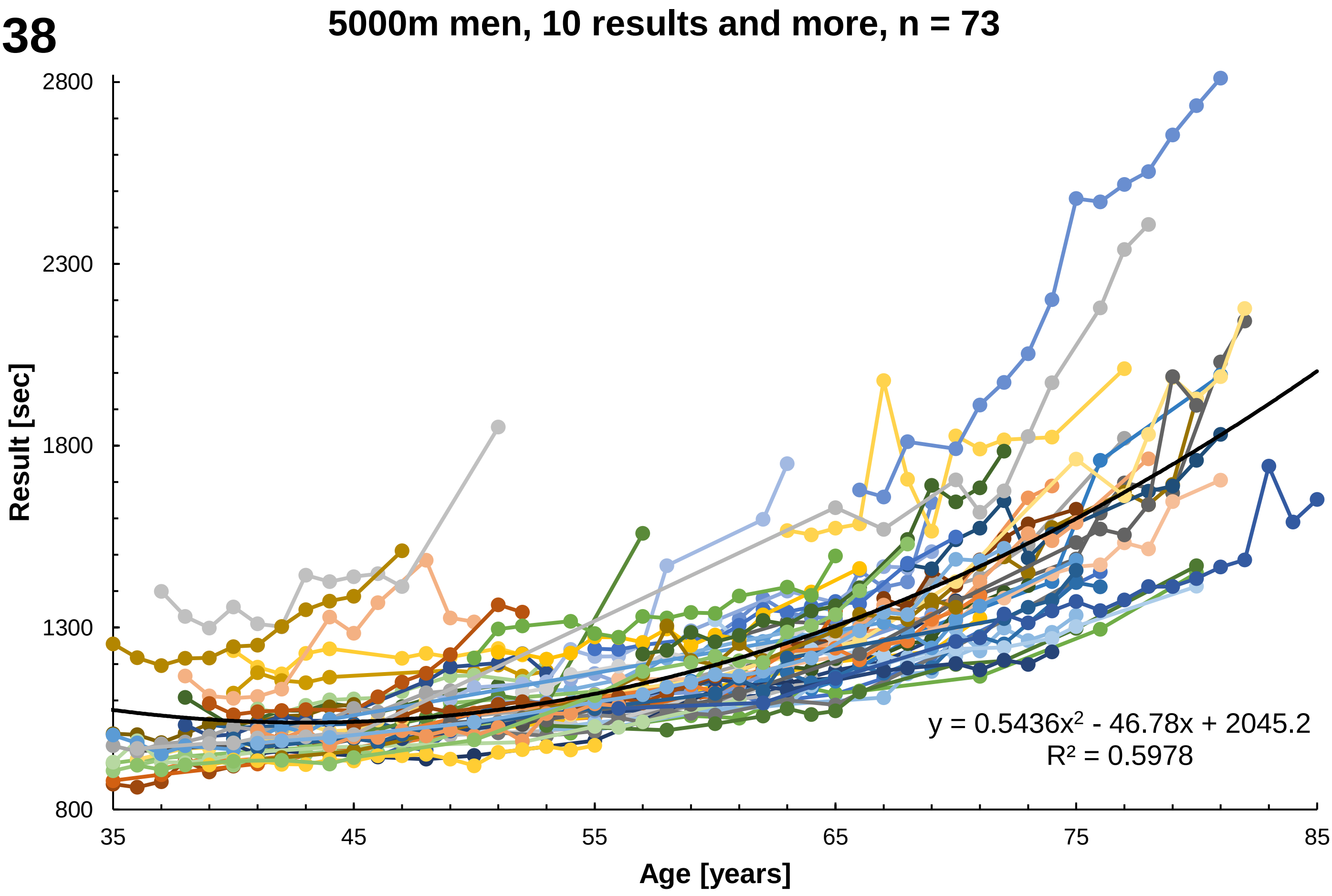
5000m men, 10 results and more, n = 73
Result [sec]
2800
2300
1800
1300
800
35
45
55
65
75
85
Age [years]
y = 0.5436x² - 46.78x + 2045.2
R² = 0.5978

39

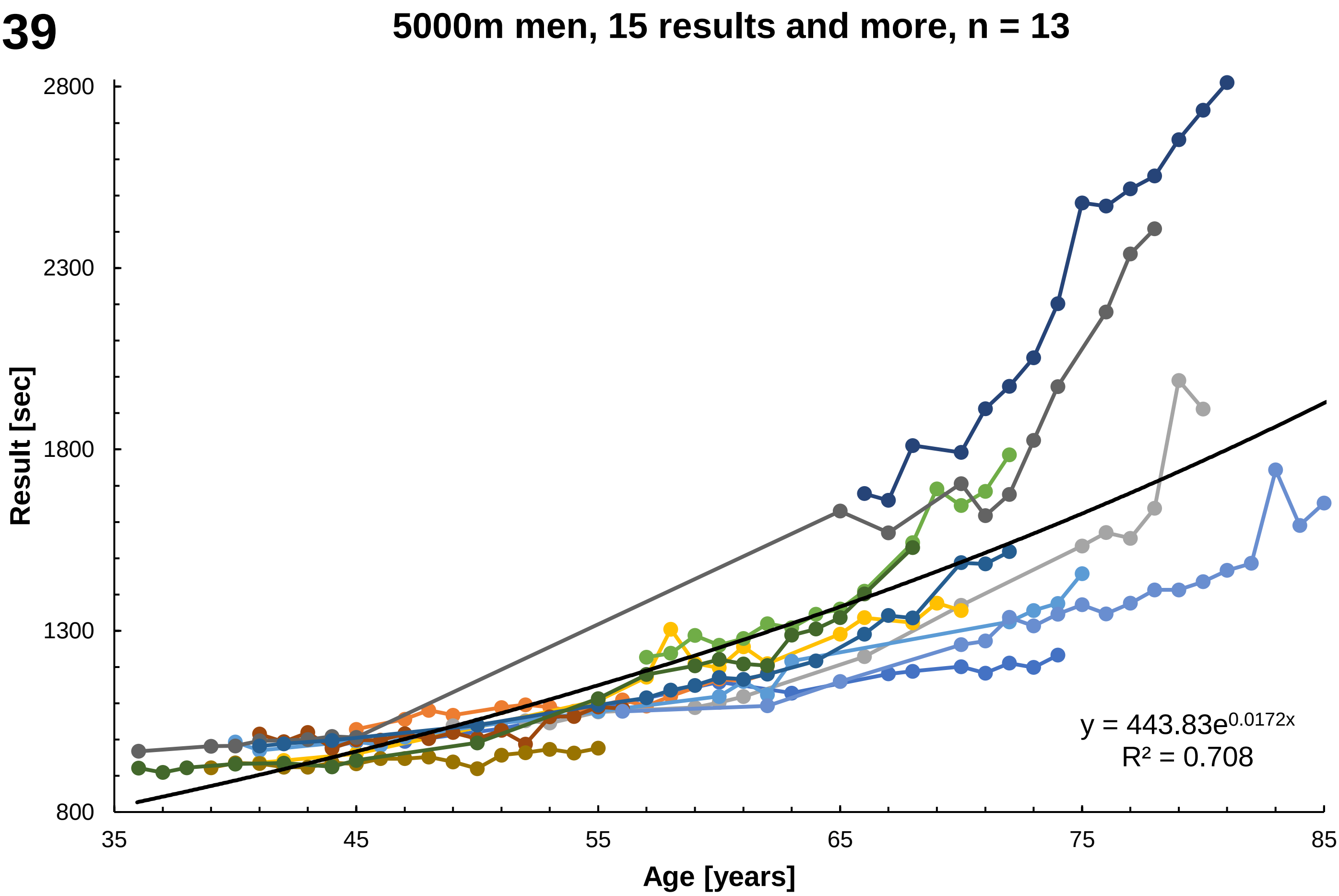
5000m men, 15 results and more, n = 13
Result [sec]
2800
2300
1800
1300
800
35
45
55
65
75
85
Age [years]
y = 443.83e^0.0172x
R² = 0.708

40

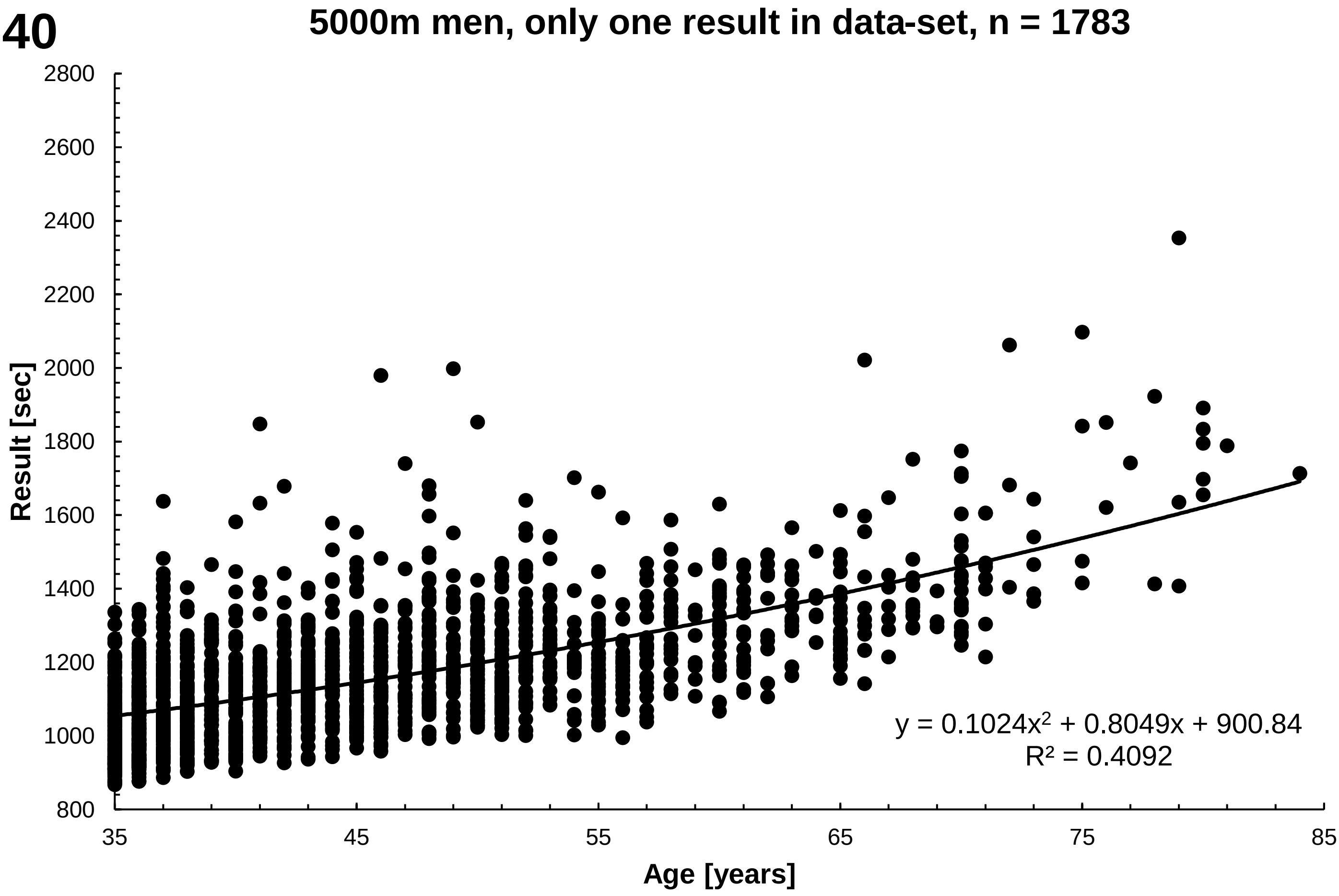
5000m men, only one result in data-set, n = 1783
Result [sec]
2800
2600
2400
2200
2000
1800
1600
1400
1200
1000
800
35
45
55
65
75
85
Age [years]
y = 0.1024x² + 0.8049x + 900.84
R² = 0.4092

41

10k men, 10 results and more, n = 61

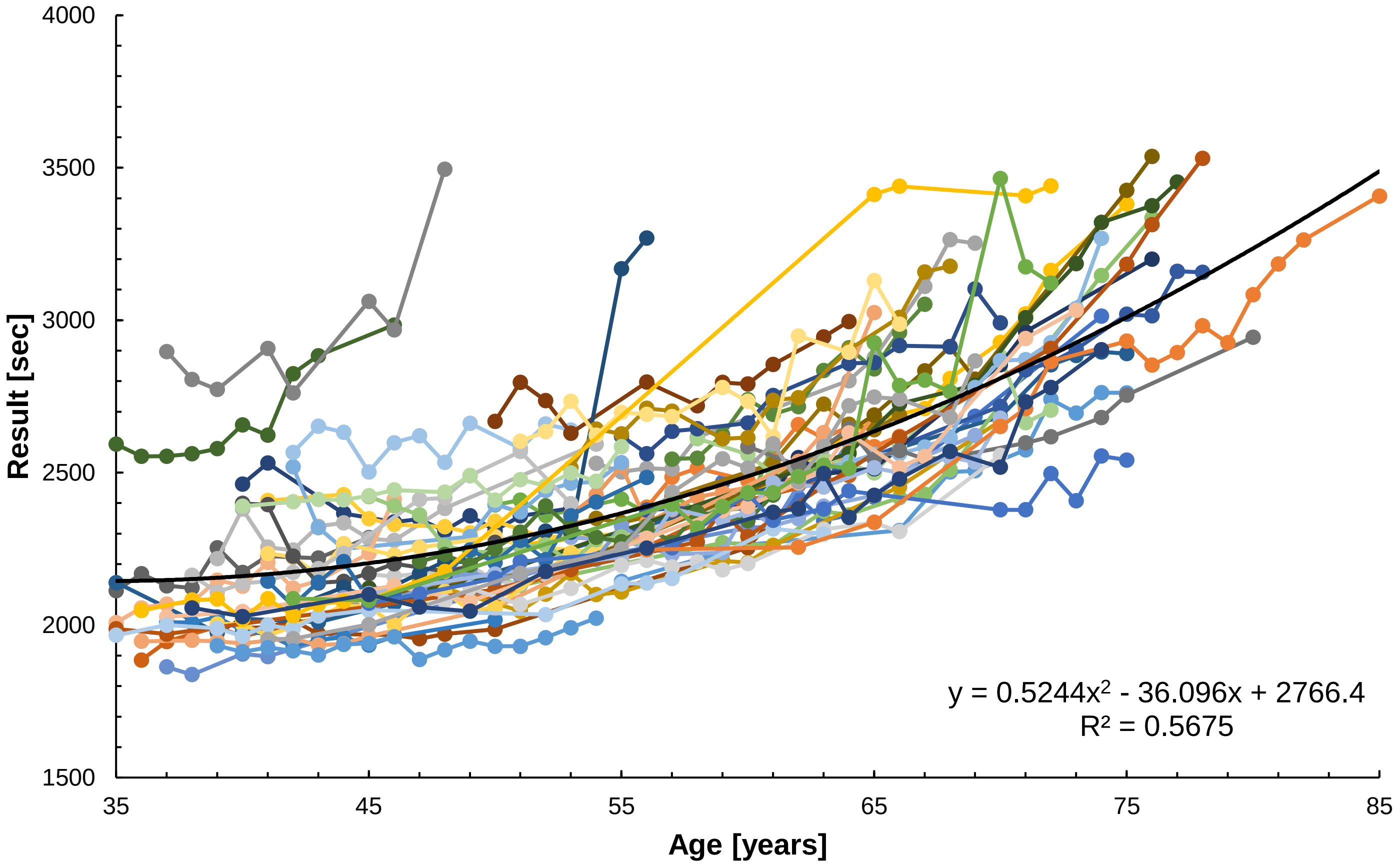

42

10k men, 15 results and more, n = 7

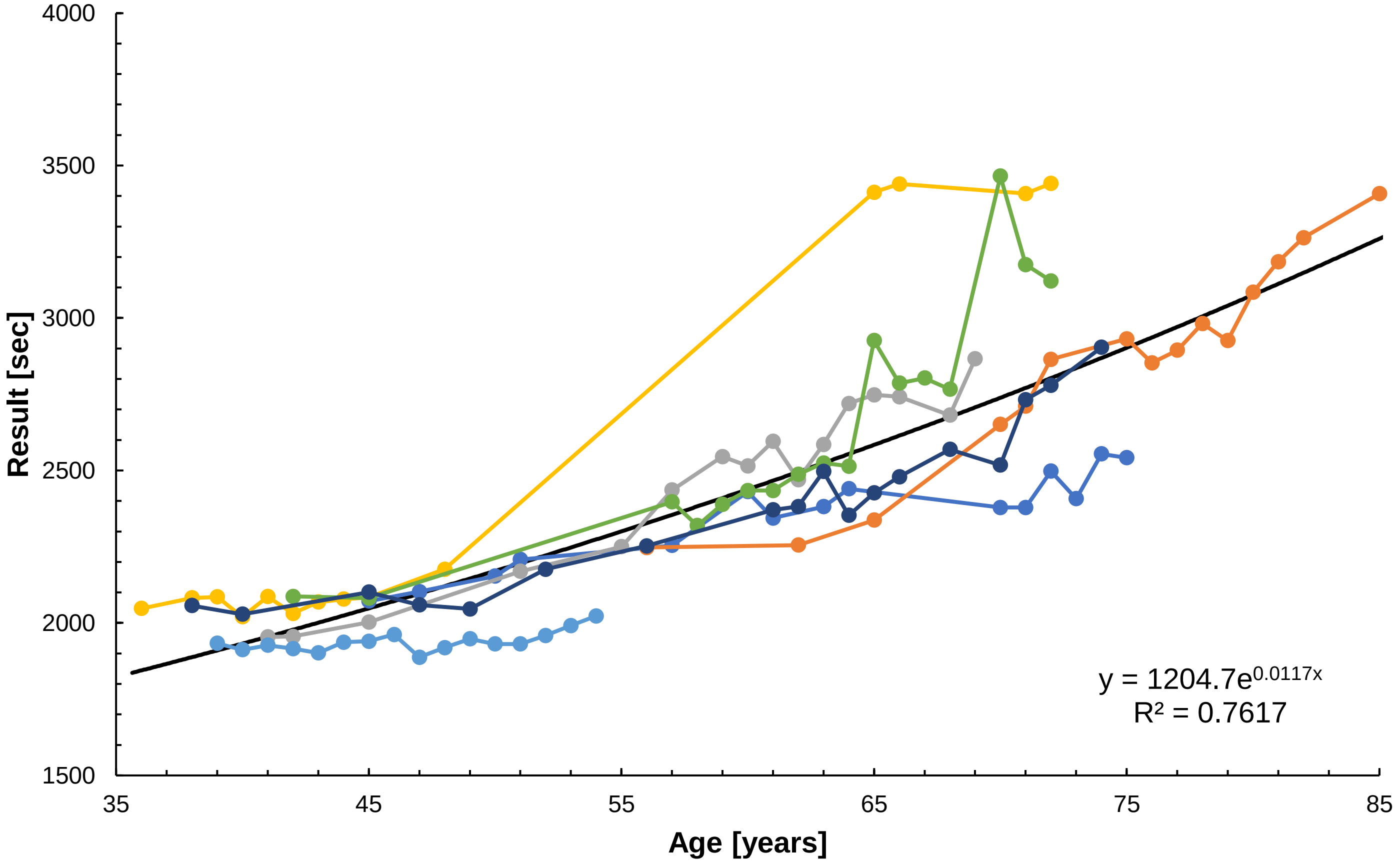

43

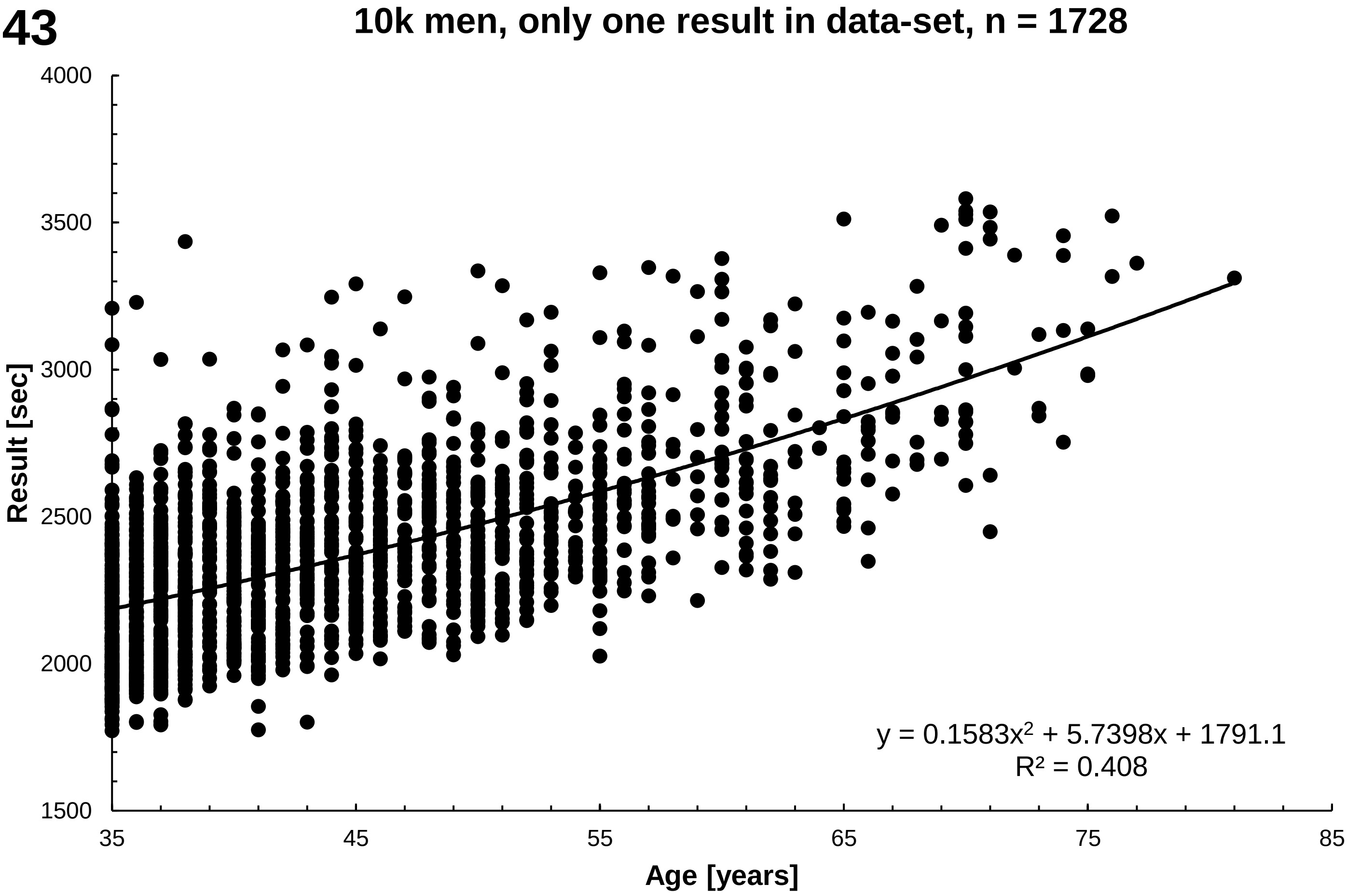
10k men, only one result in data-set, n = 1728
Result [sec]
4000
3500
3000
2500
2000
1500
35
45
55
65
75
85
Age [years]
y = 0.1583x² + 5.7398x + 1791.1
R² = 0.408

44

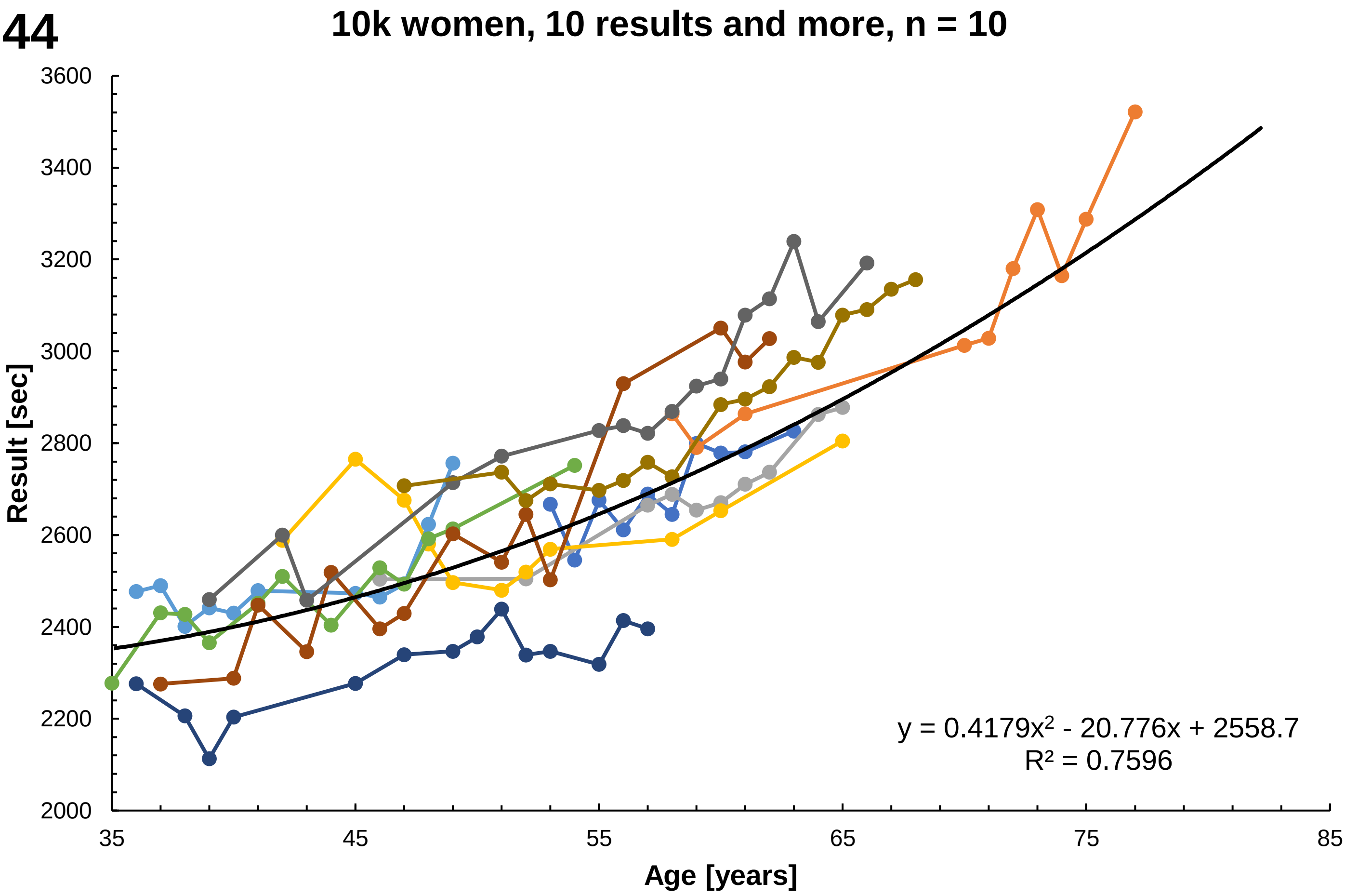
10k women, 10 results and more, n = 10
Result [sec]
3600
3400
3200
3000
2800
2600
2400
2200
2000
35
45
55
65
75
85
Age [years]
y = 0.4179x² - 20.776x + 2558.7
R² = 0.7596

45

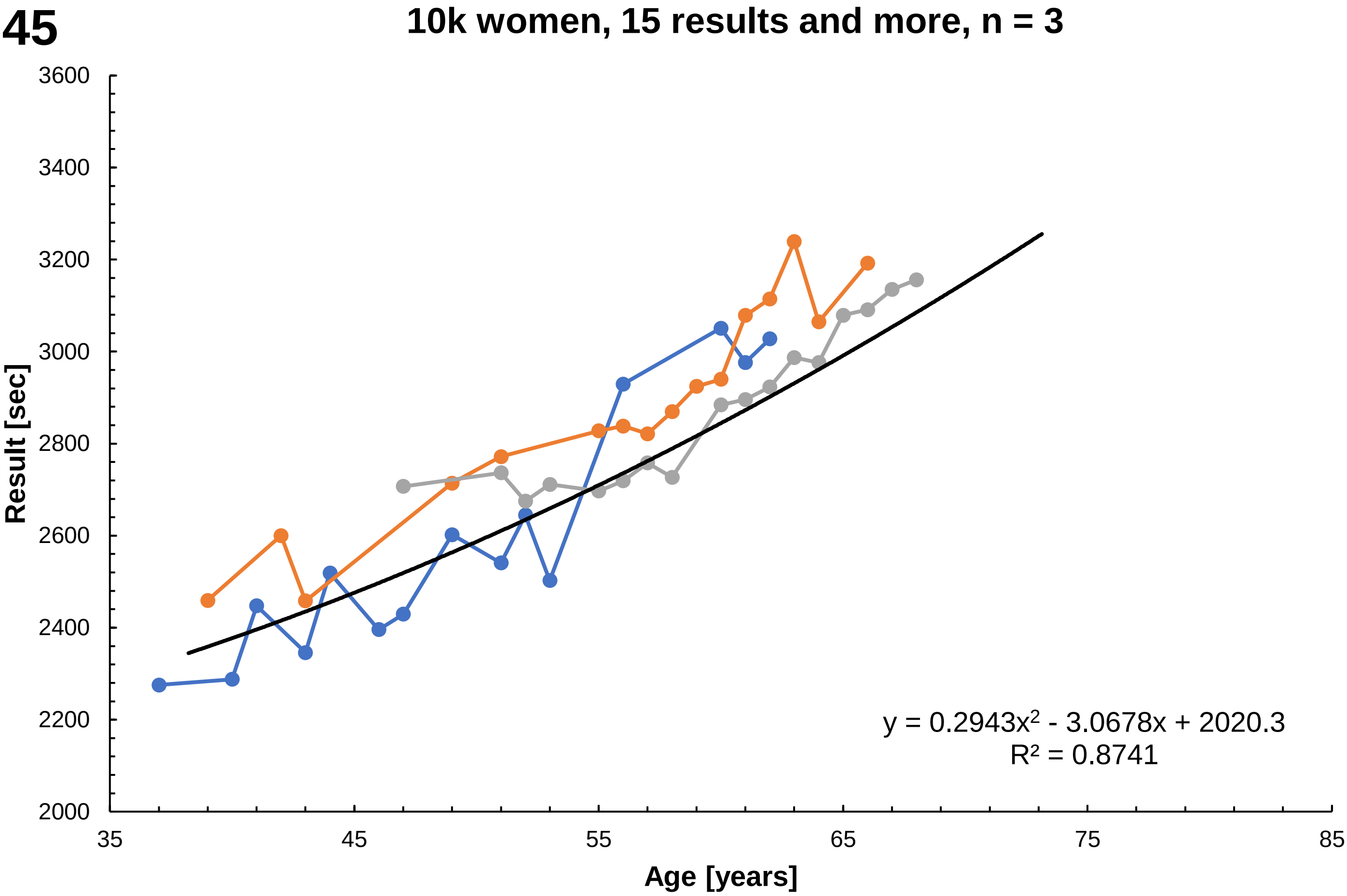
10k women, 15 results and more, n = 3
3600
3400
3200
3000
2800
2600
2400
2200
2000
Result [sec]
35
45
55
65
75
85
Age [years]
y = 0.2943x² - 3.0678x + 2020.3
R² = 0.8741

46

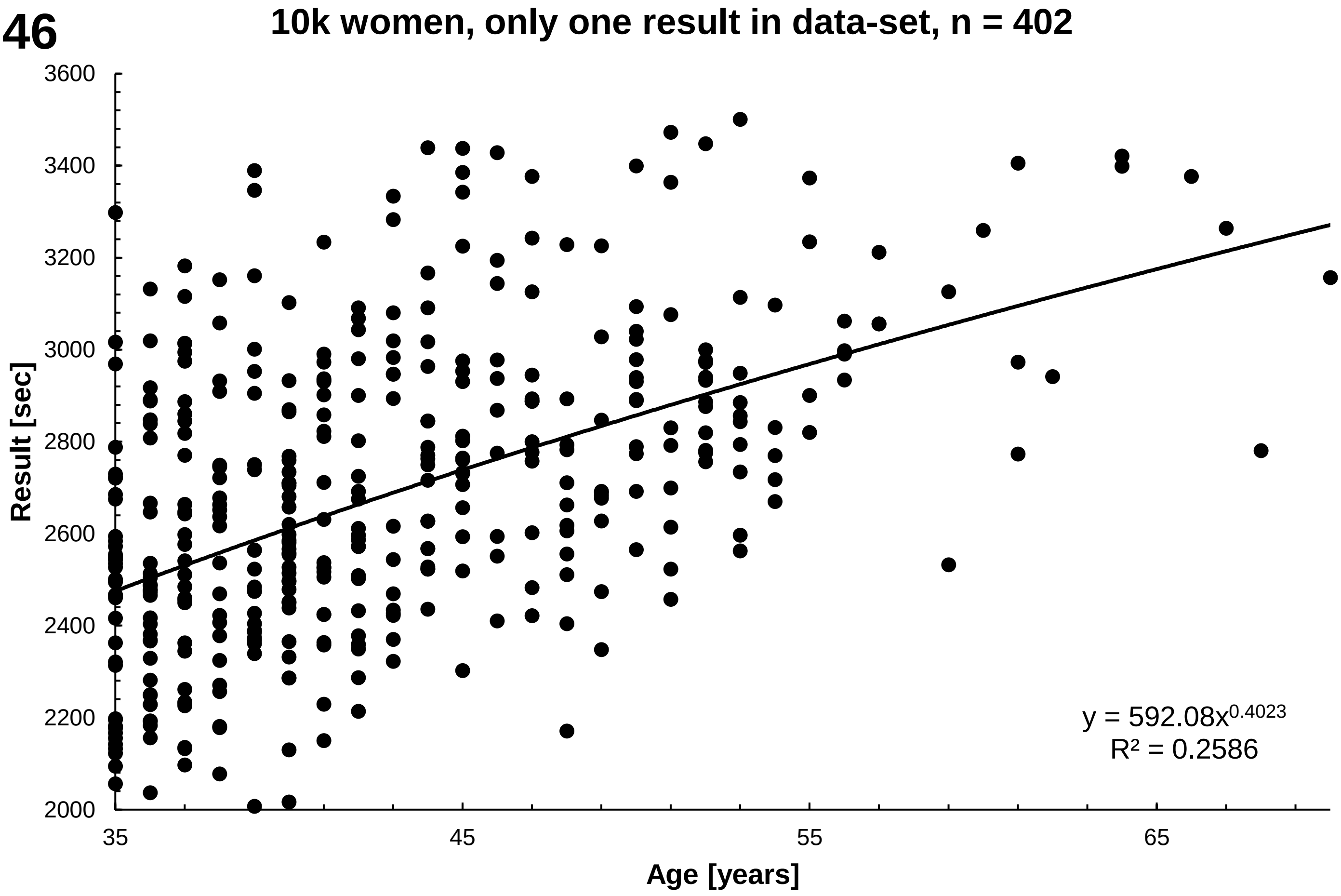
10k women, only one result in data-set, n = 402
3600
3400
3200
3000
2800
2600
2400
2200
2000
Result [sec]
35
45
55
65
Age [years]
y = 592.08x^0.4023
R² = 0.2586

47

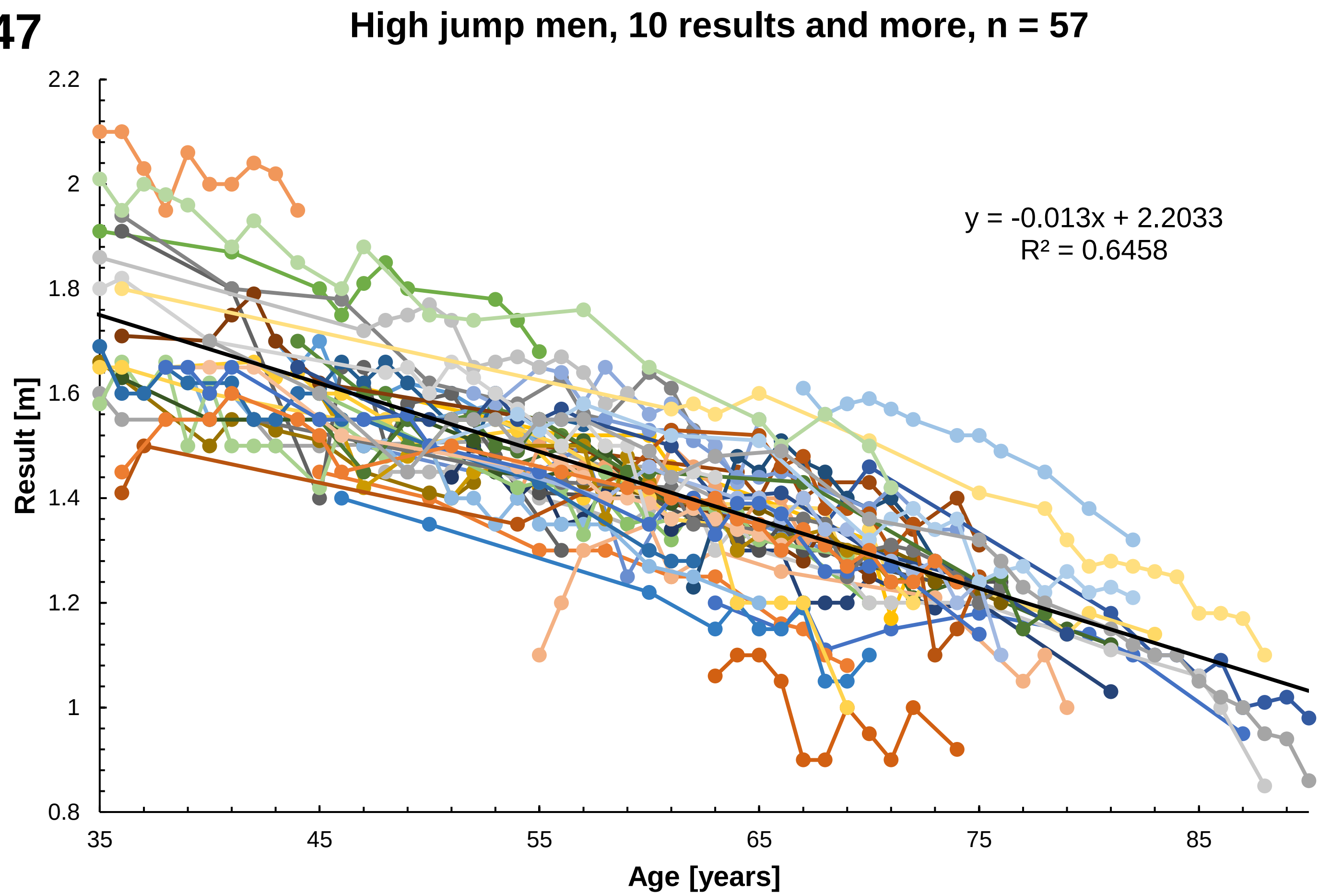
High jump men, 10 results and more, n = 57
y = -0.013x + 2.2033
R² = 0.6458
Result [m]
Age [years]

48

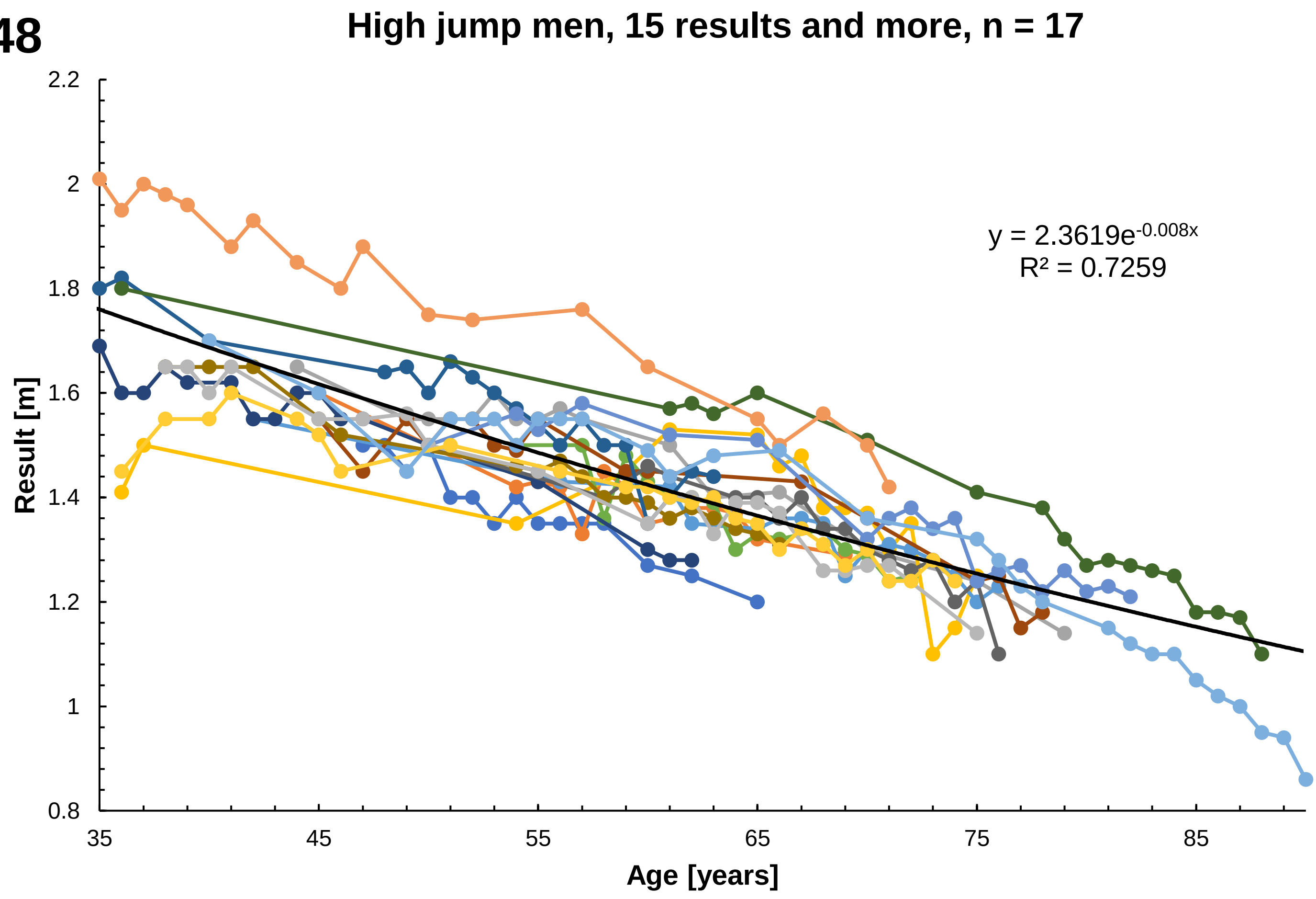
High jump men, 15 results and more, n = 17
y = 2.3619e^-0.008x
R² = 0.7259
Result [m]
Age [years]

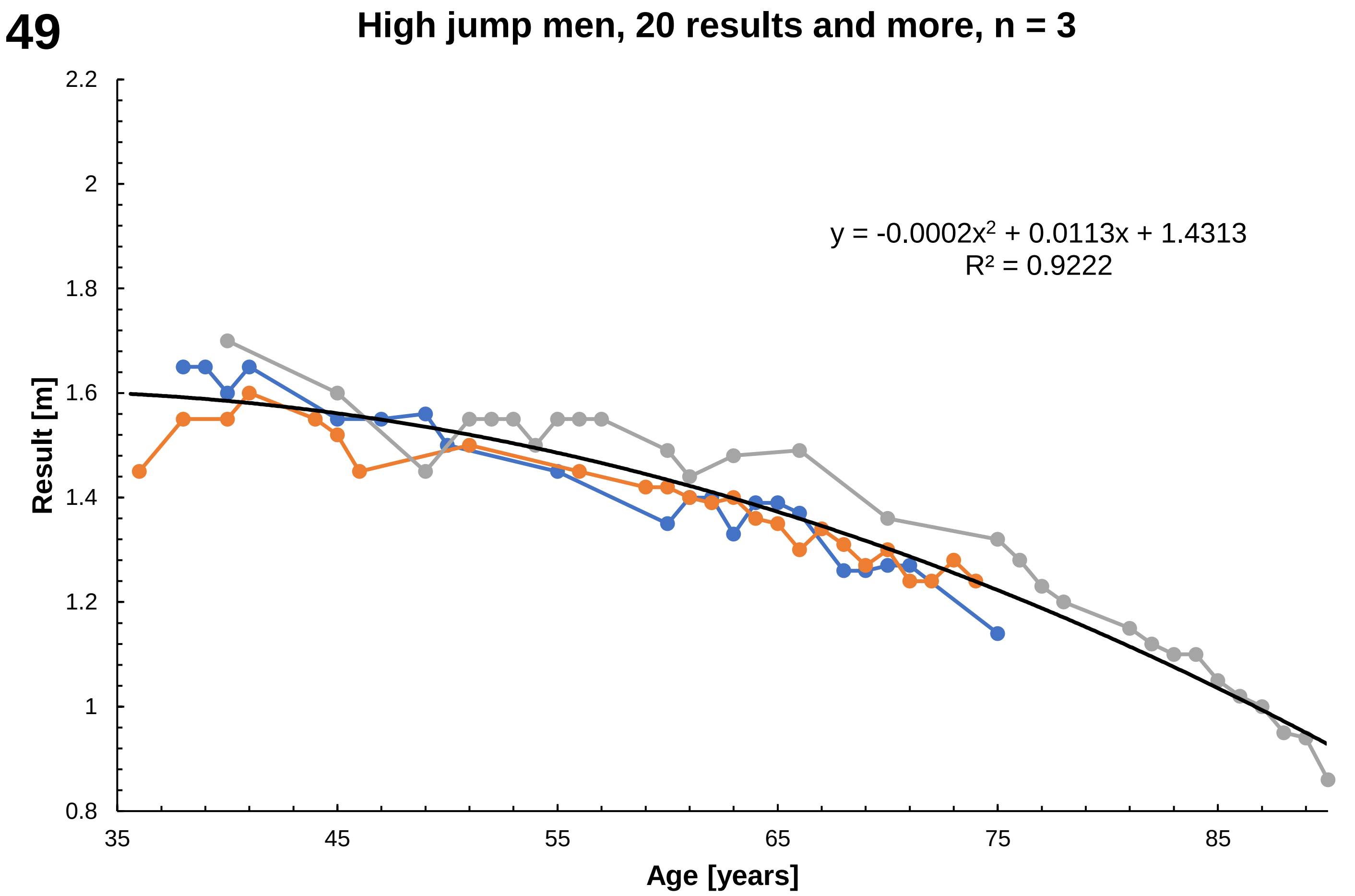

49
High jump men, 20 results and more, n = 3
y = -0.0002x2 + 0.0113x + 1.4313
R² = 0.9222
Result [m]
Age [years]
2.2
2
1.8
1.6
1.4
1.2
1
0.8
35
45
55
65
75
85

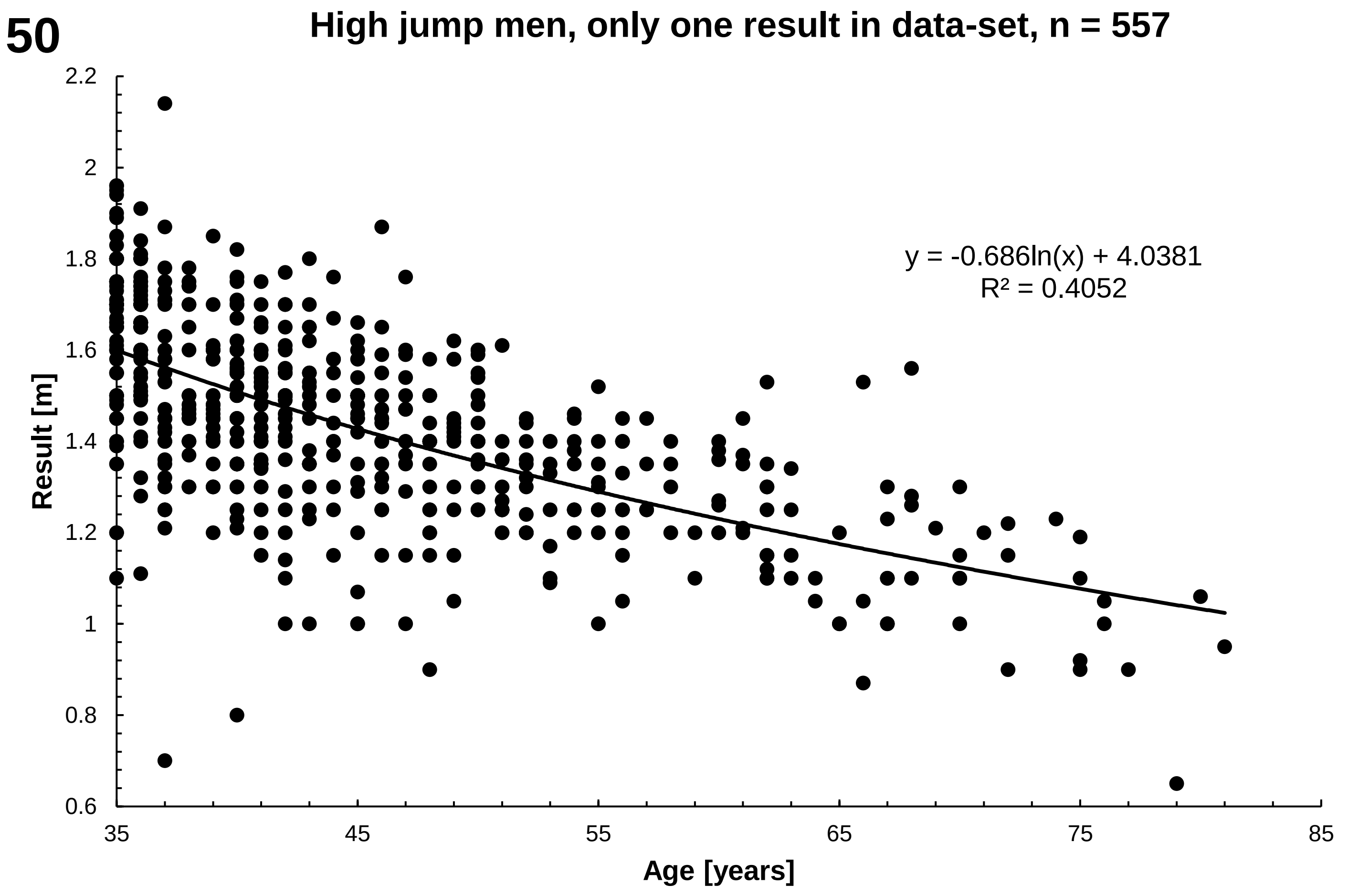

50
High jump men, only one result in data-set, n = 557
y = -0.686ln(x) + 4.0381
R² = 0.4052
Result [m]
Age [years]
2.2
2
1.8
1.6
1.4
1.2
1
0.8
0.6
35
45
55
65
75
85

51

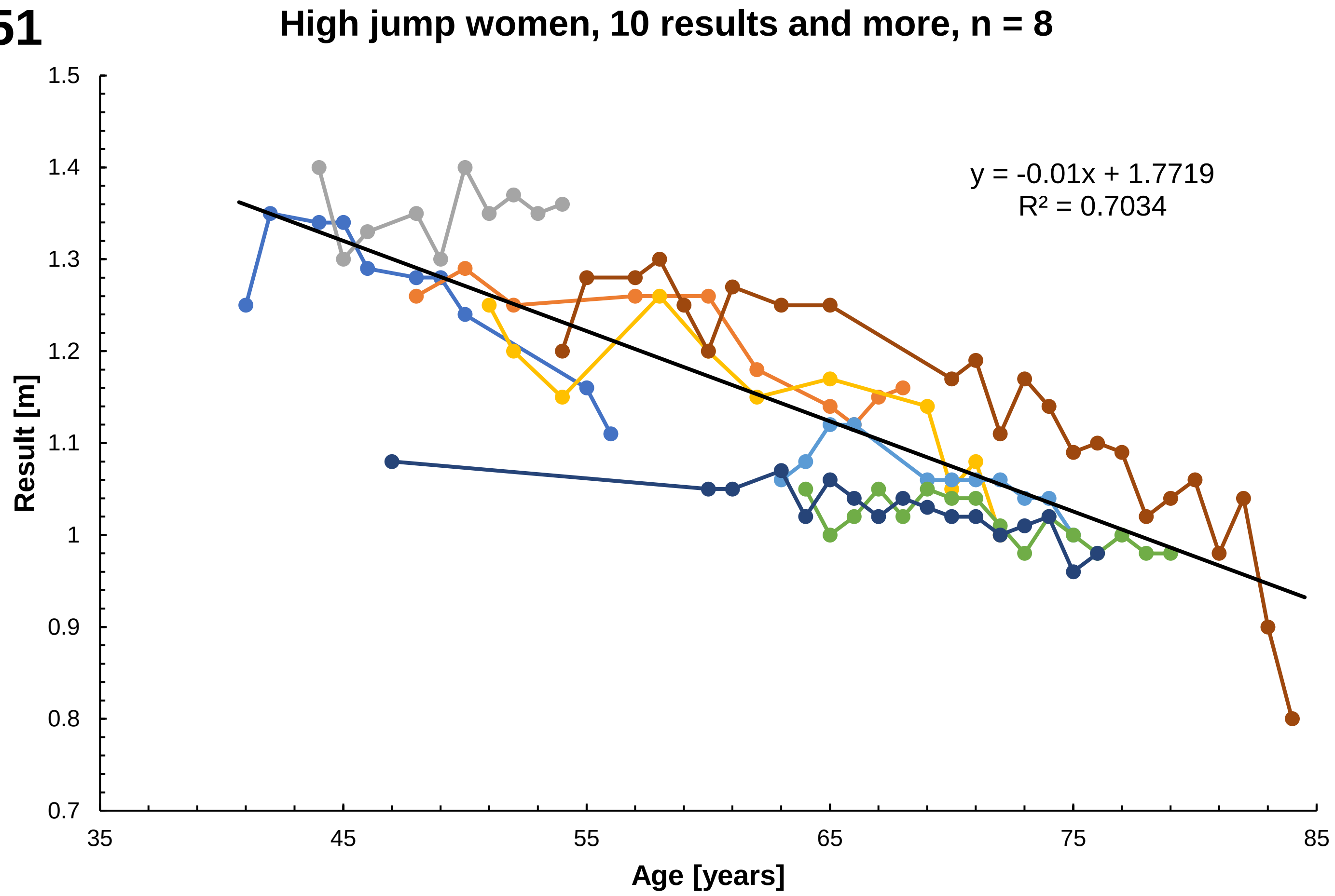
High jump women, 10 results and more, n = 8
y = -0.01x + 1.7719
R² = 0.7034
Result [m]
Age [years]
1.5
1.4
1.3
1.2
1.1
1
0.9
0.8
0.7
35
45
55
65
75
85

52

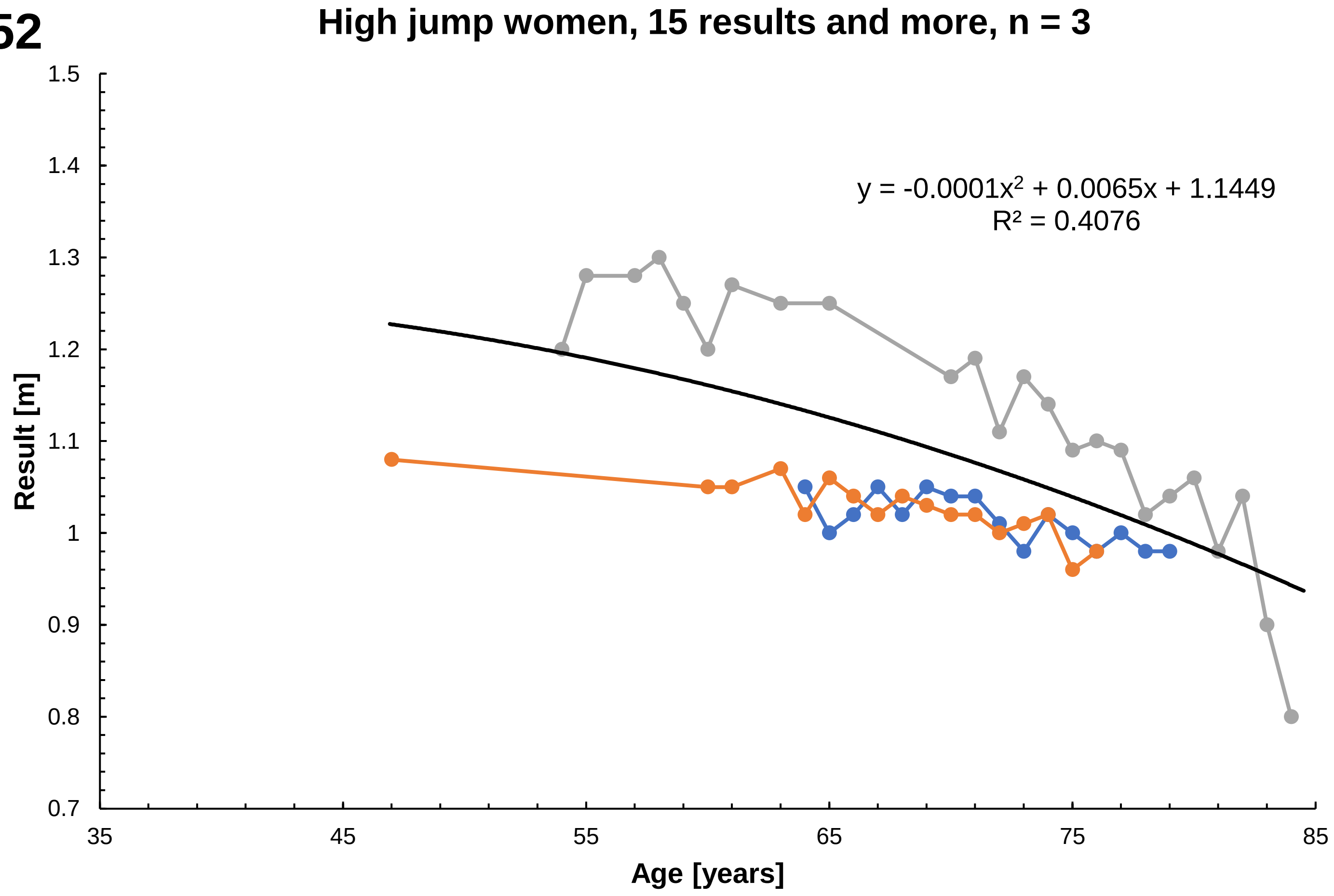
High jump women, 15 results and more, n = 3
y = -0.0001x² + 0.0065x + 1.1449
R² = 0.4076
Result [m]
Age [years]
1.5
1.4
1.3
1.2
1.1
1
0.9
0.8
0.7
35
45
55
65
75
85

53

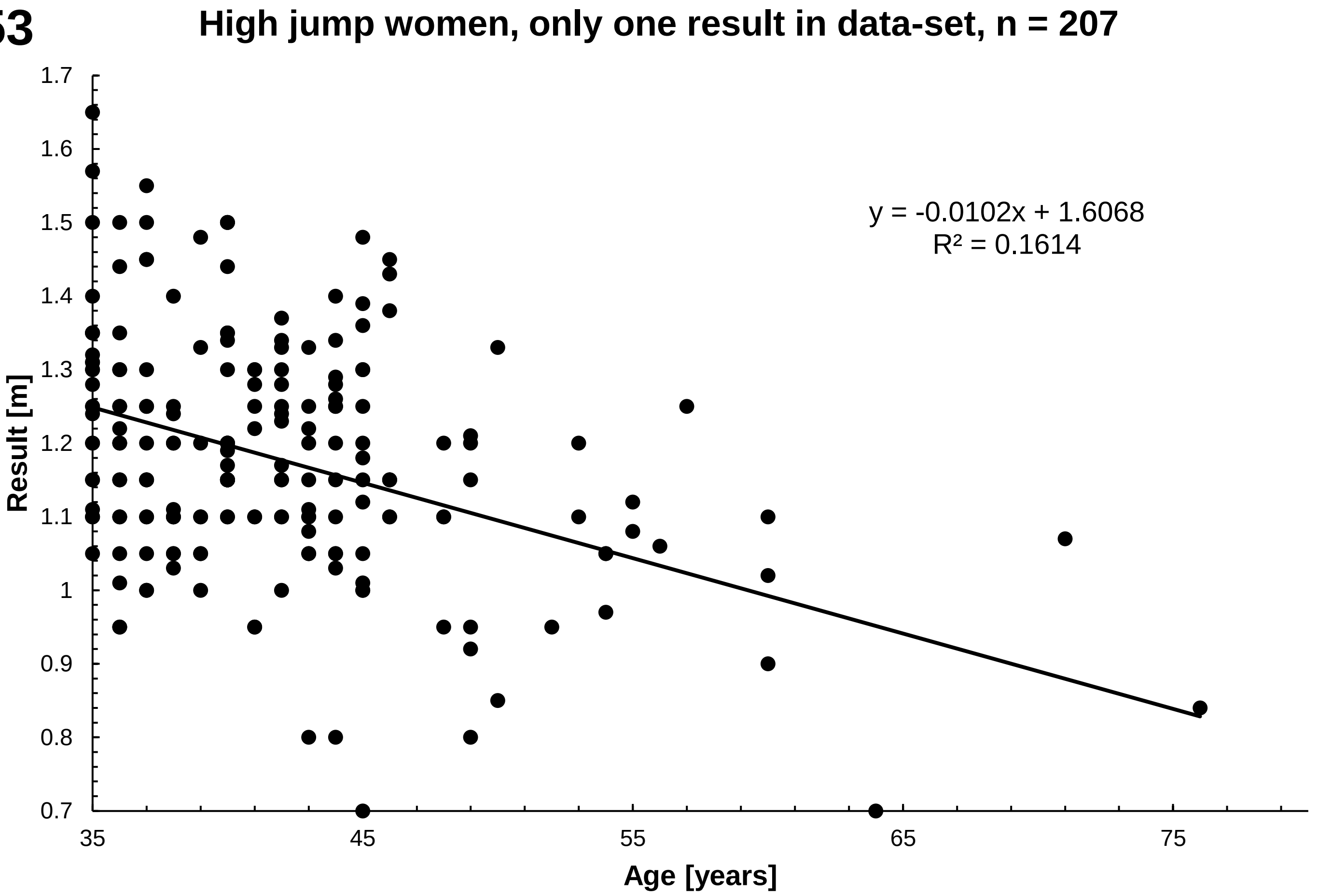
High jump women, only one result in data-set, n = 207
y = -0.0102x + 1.6068
R² = 0.1614
Result [m]
Age [years]
1.7
1.6
1.5
1.4
1.3
1.2
1.1
1
0.9
0.8
0.7
35
45
55
65
75

54

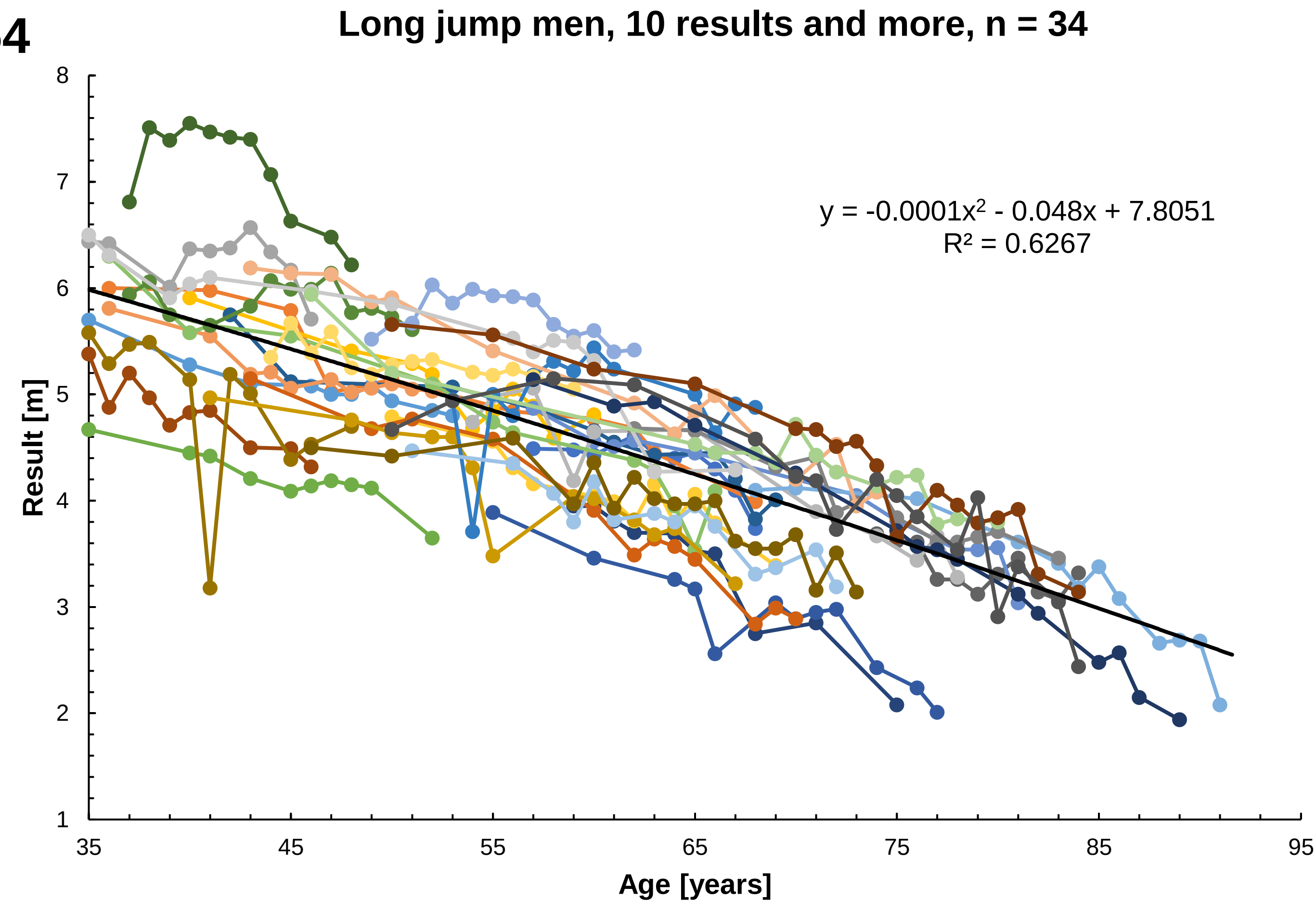
Long jump men, 10 results and more, n = 34
y = -0.0001x² - 0.048x + 7.8051
R² = 0.6267
Result [m]
Age [years]
8
7
6
5
4
3
2
1
35
45
55
65
75
85
95

55 **Long jump men, 15 results and more, n = 5**

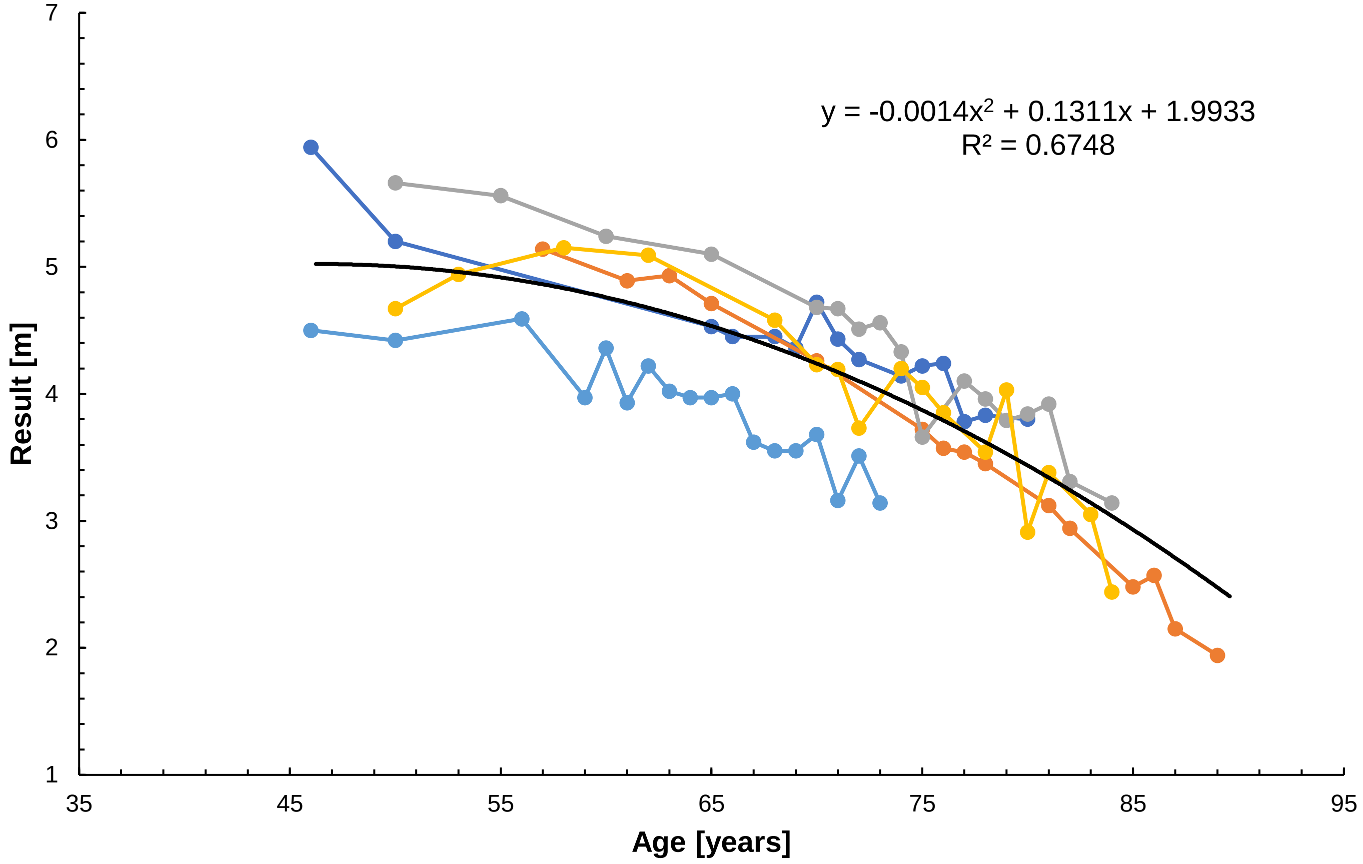

56 **Long jump men, one result only, n = 605**

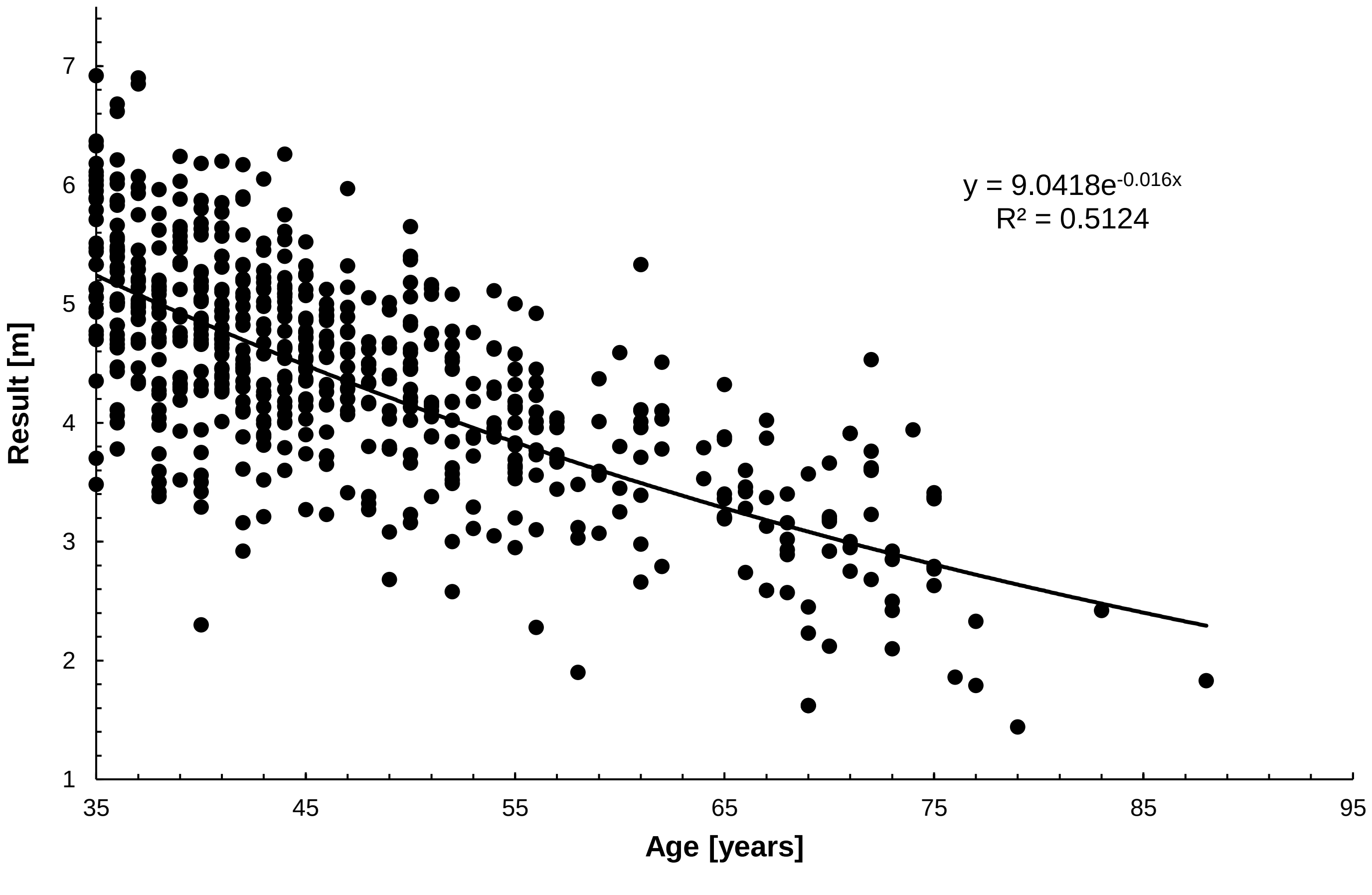

57

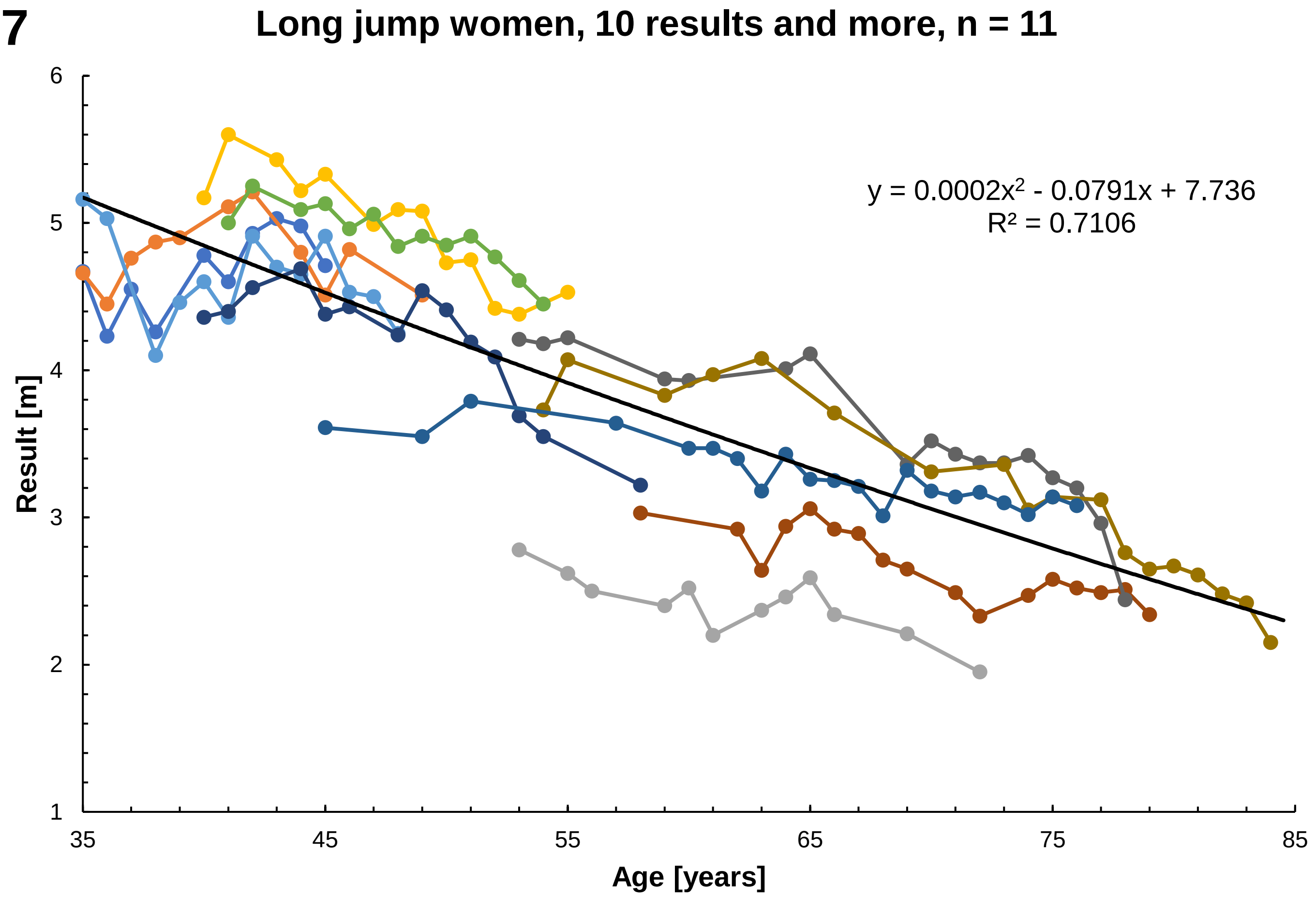

Long jump women, 10 results and more, n = 11
y = 0.0002x² - 0.0791x + 7.736
R² = 0.7106
Result [m]
Age [years]
1
2
3
4
5
6
35
45
55
65
75
85

58

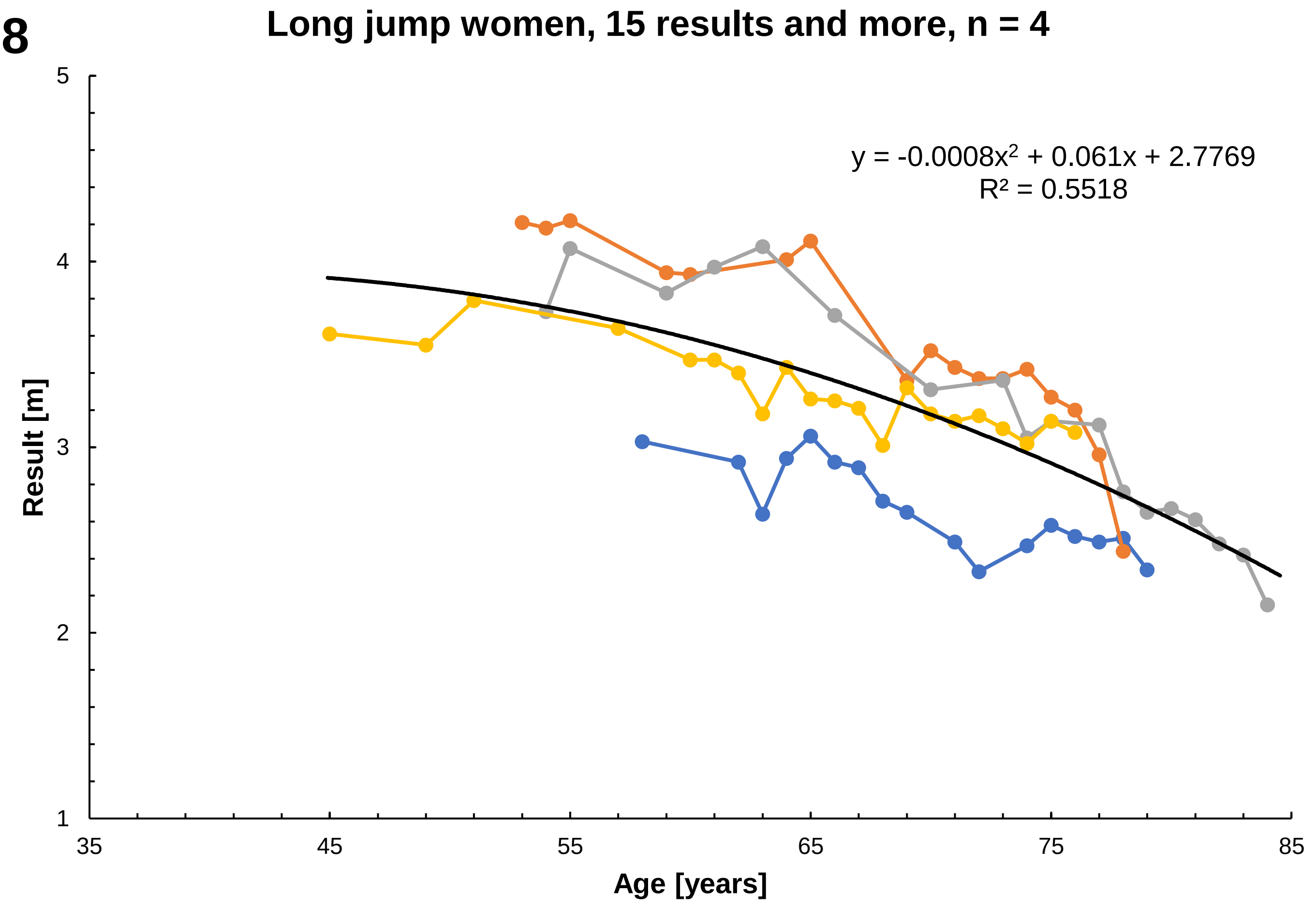

Long jump women, 15 results and more, n = 4
y = -0.0008x² + 0.061x + 2.7769
R² = 0.5518
Result [m]
Age [years]
1
2
3
4
5
35
45
55
65
75
85

59

Long jump women, only one result in data-set, n = 249

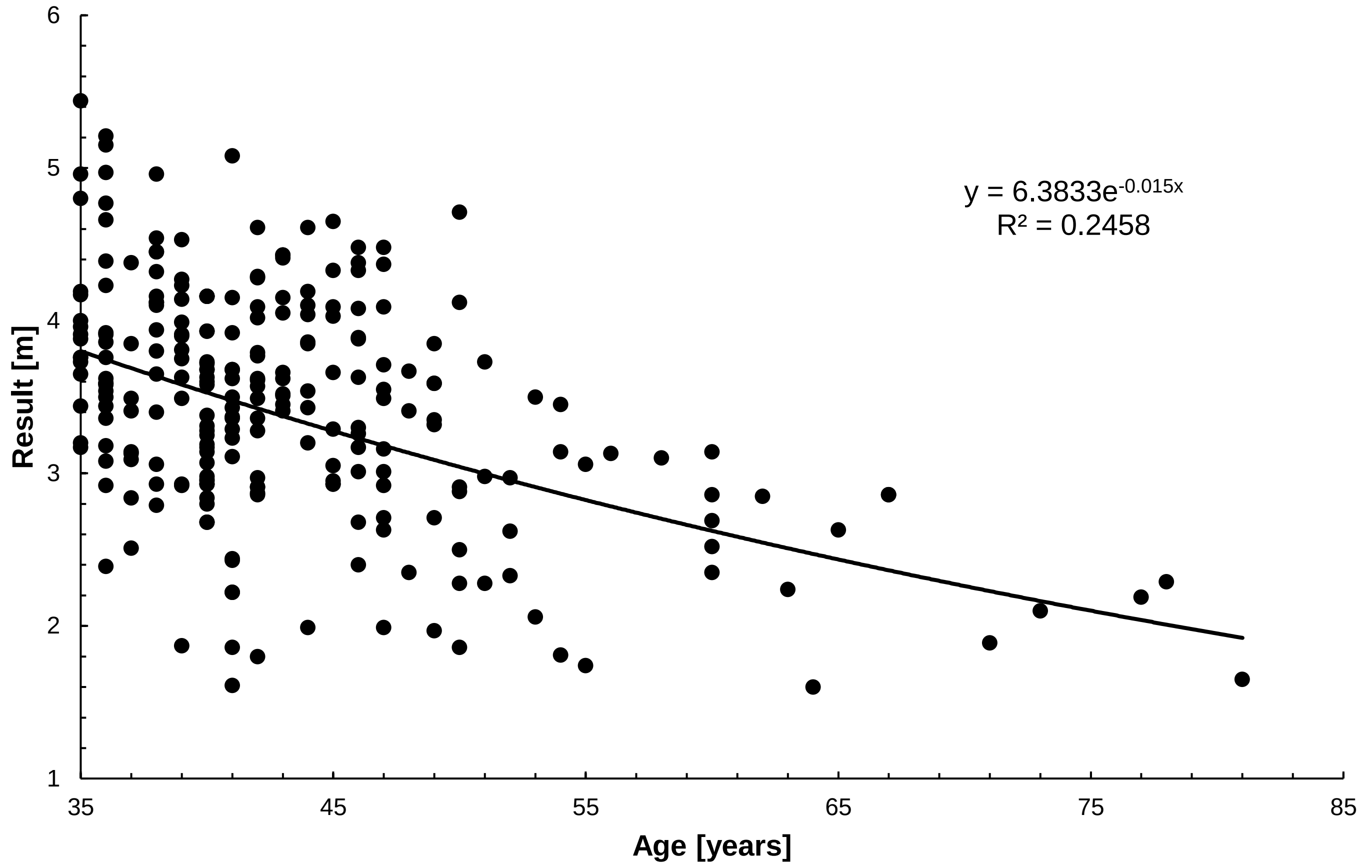

60

Triple jump men, 10 results and more, n = 16

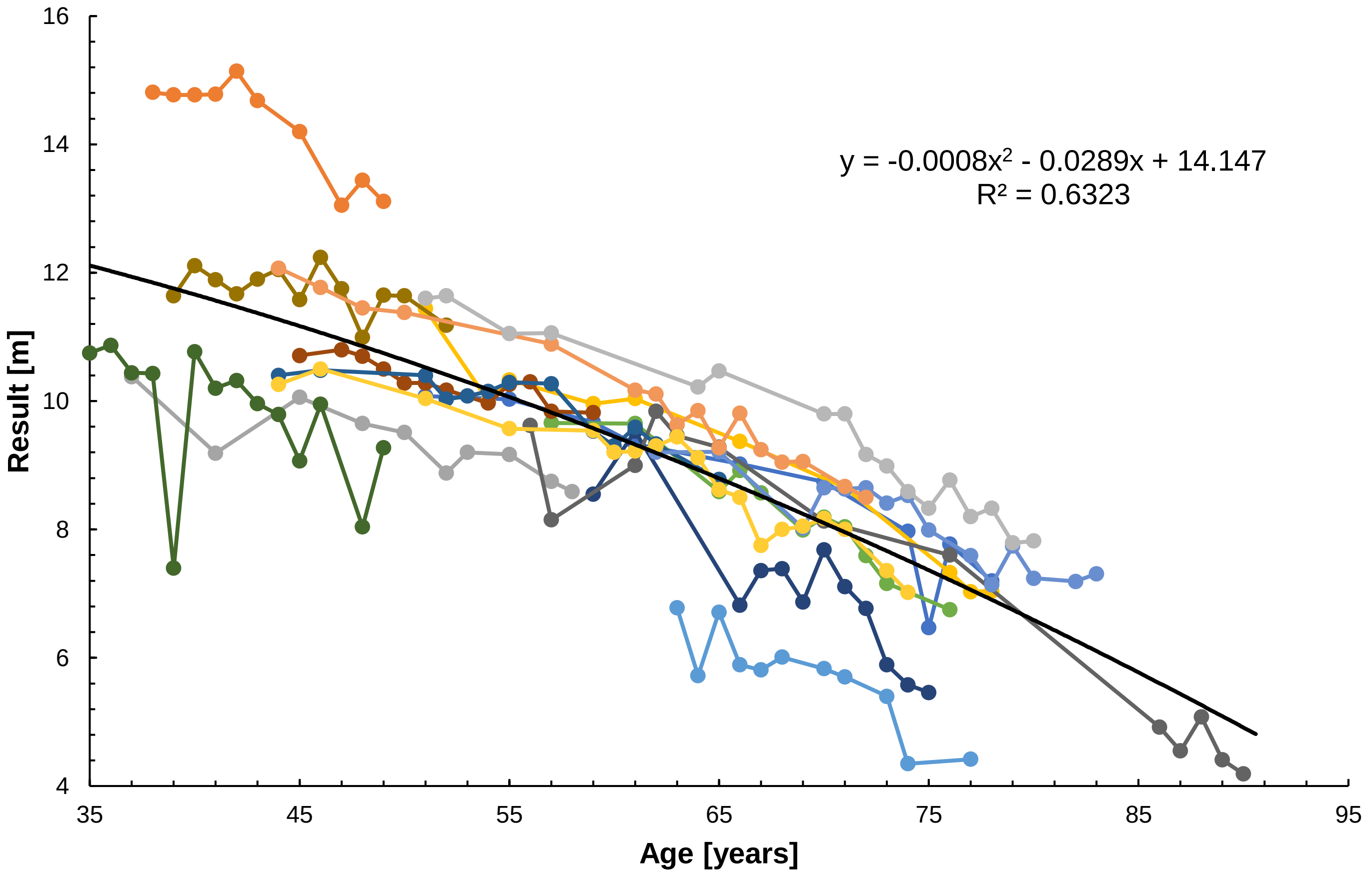

61

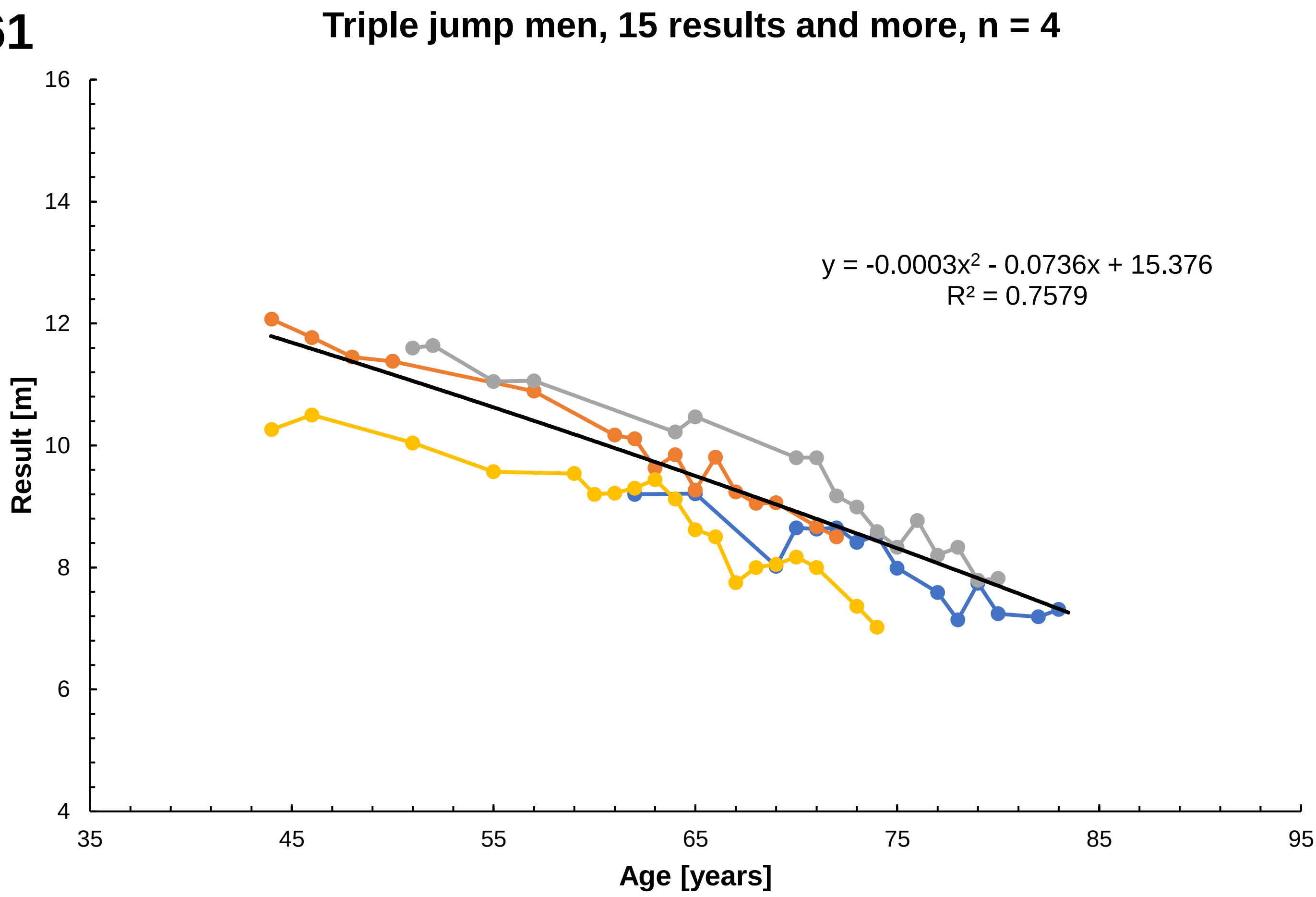
Triple jump men, 15 results and more, n = 4
y = -0.0003x² - 0.0736x + 15.376
R² = 0.7579
Result [m]
Age [years]
16
14
12
10
8
6
4
35
45
55
65
75
85
95

62

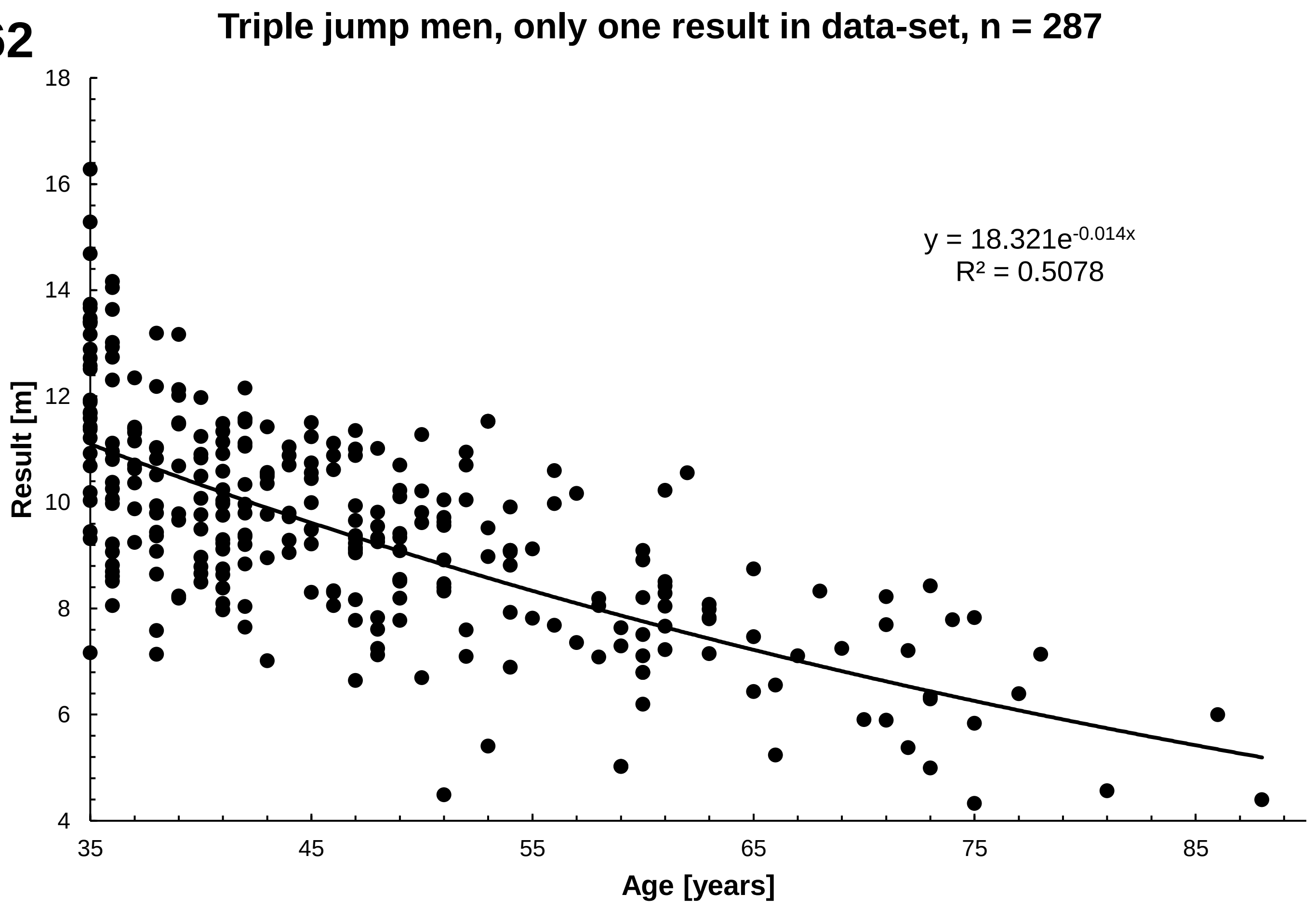
Triple jump men, only one result in data-set, n = 287
y = 18.321e-0.014x
R² = 0.5078
Result [m]
Age [years]
18
16
14
12
10
8
6
4
35
45
55
65
75
85

63

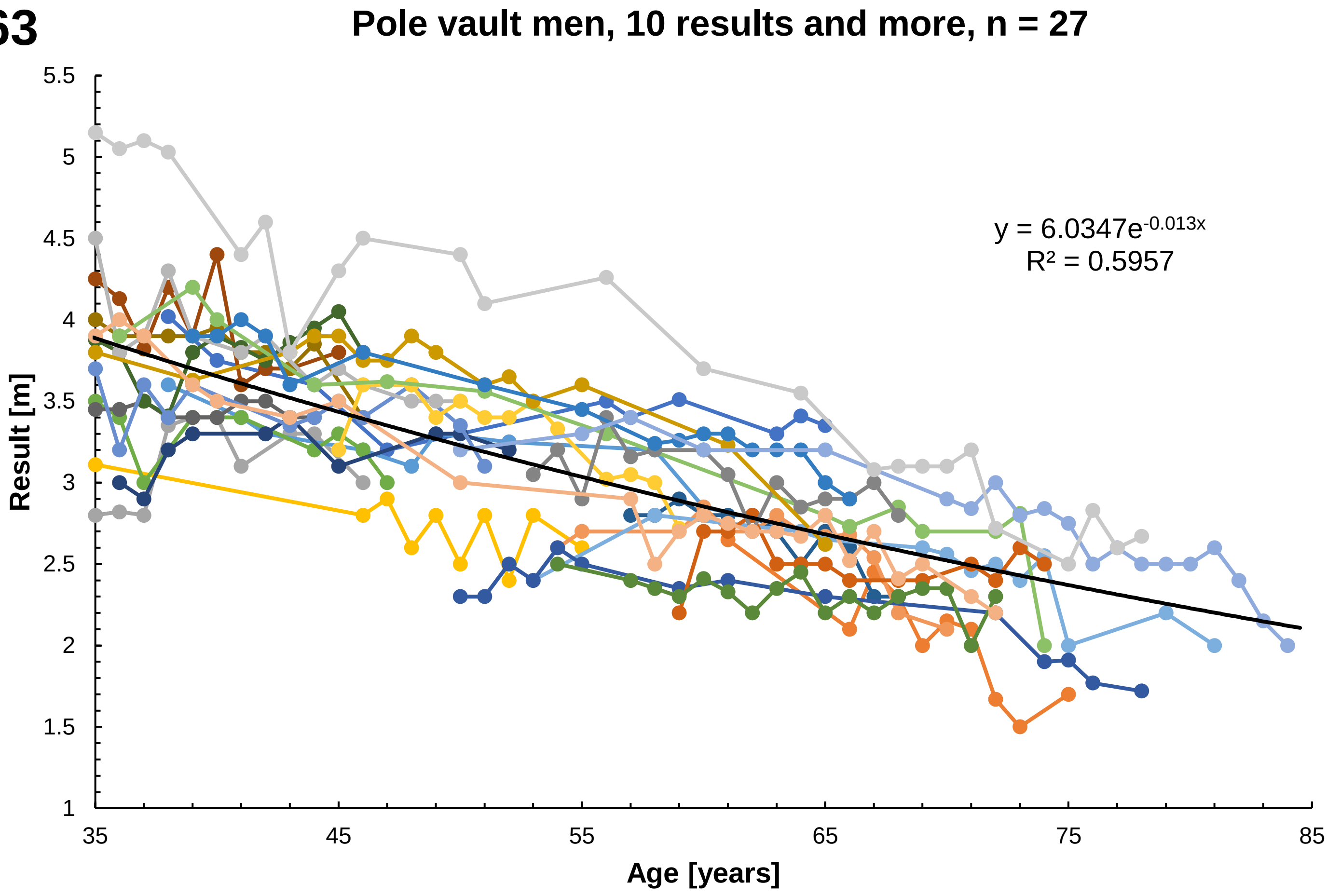
Pole vault men, 10 results and more, n = 27
y = 6.0347e^-0.013x
R² = 0.5957
Result [m]
Age [years]
5.5
5
4.5
4
3.5
3
2.5
2
1.5
1
35
45
55
65
75
85

64

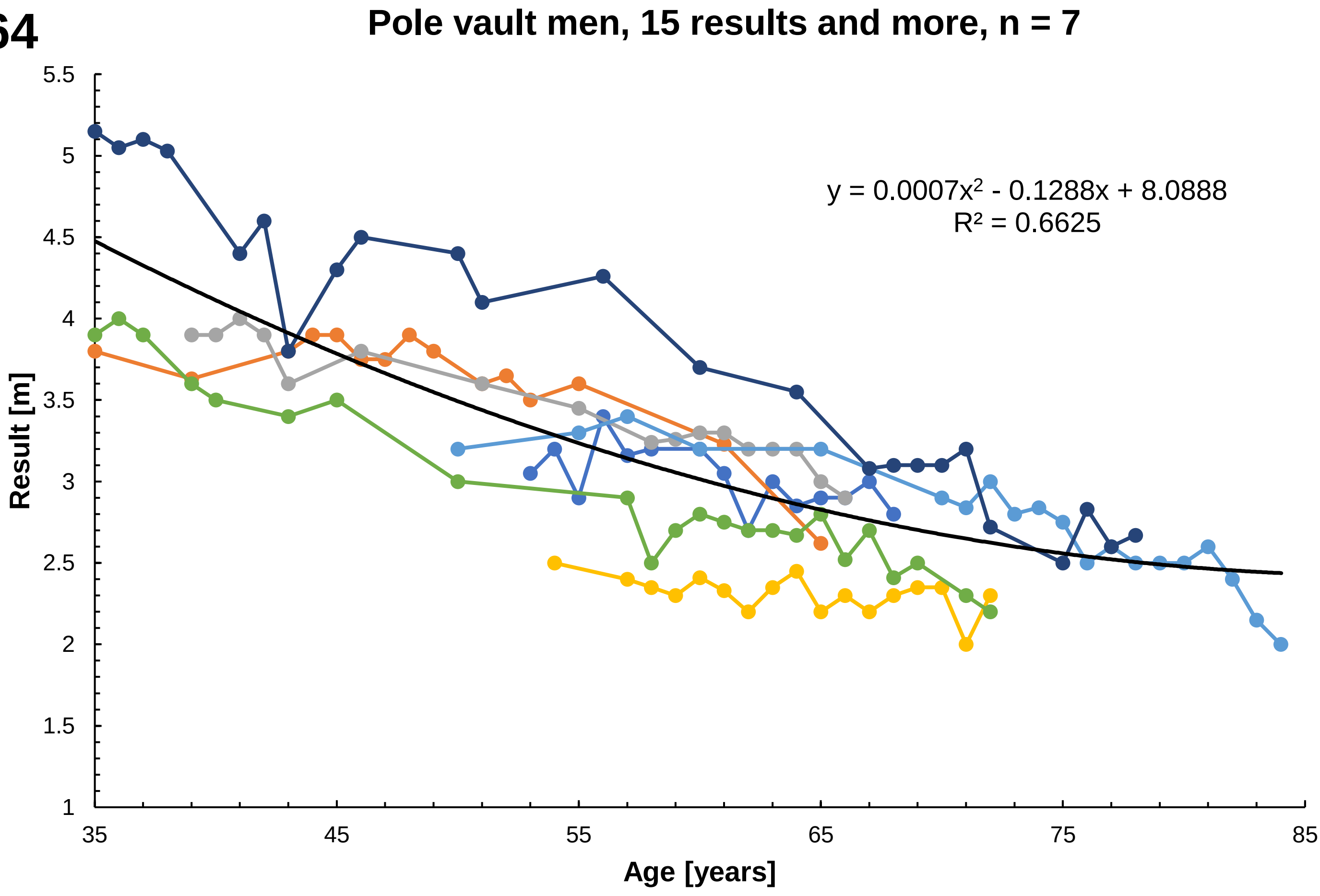
Pole vault men, 15 results and more, n = 7
y = 0.0007x² - 0.1288x + 8.0888
R² = 0.6625
Result [m]
Age [years]
5.5
5
4.5
4
3.5
3
2.5
2
1.5
1
35
45
55
65
75
85

65

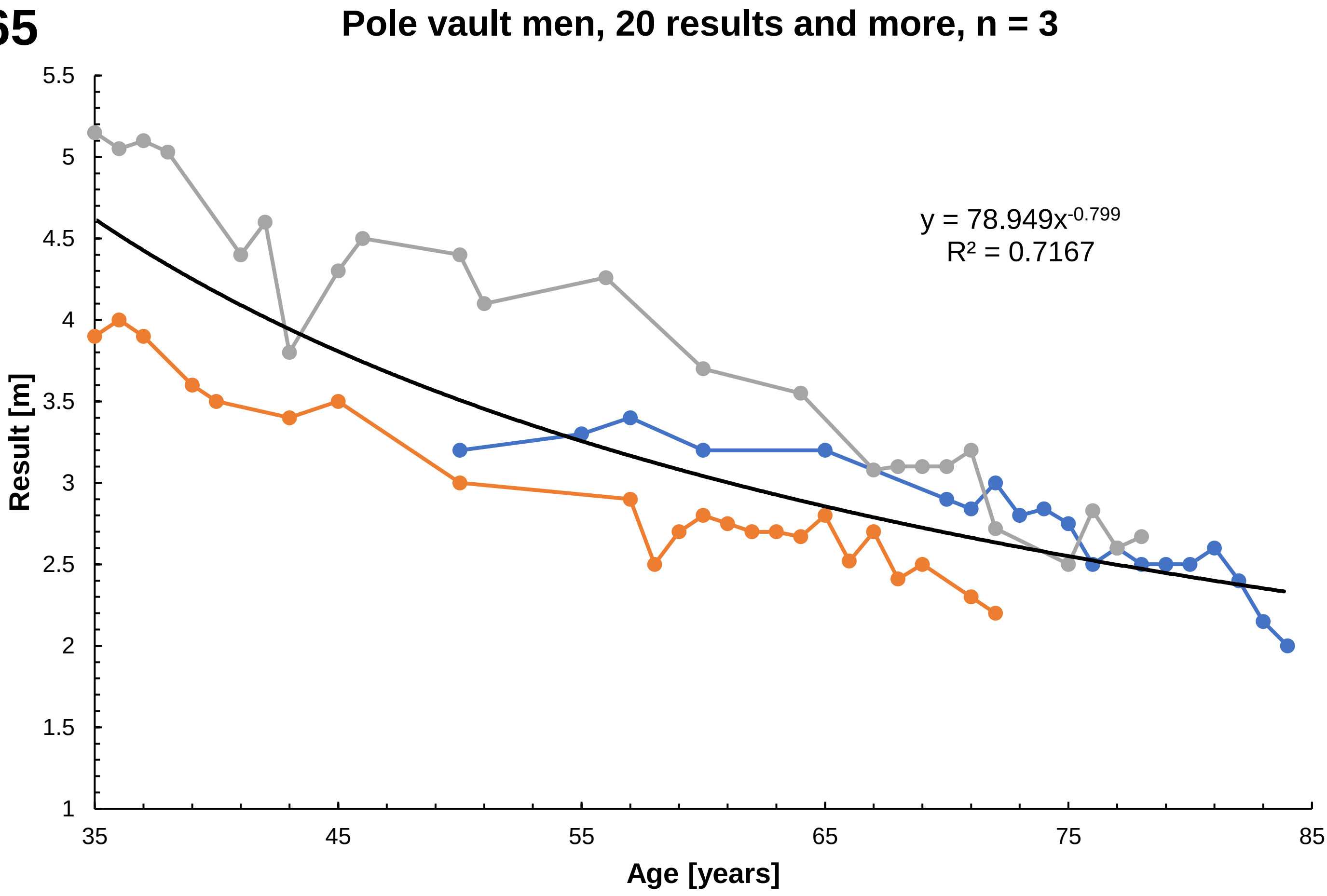
Pole vault men, 20 results and more, n = 3
y = 78.949x^-0.799
R² = 0.7167
Result [m]
Age [years]
5.5
5
4.5
4
3.5
3
2.5
2
1.5
1
35
45
55
65
75
85

66

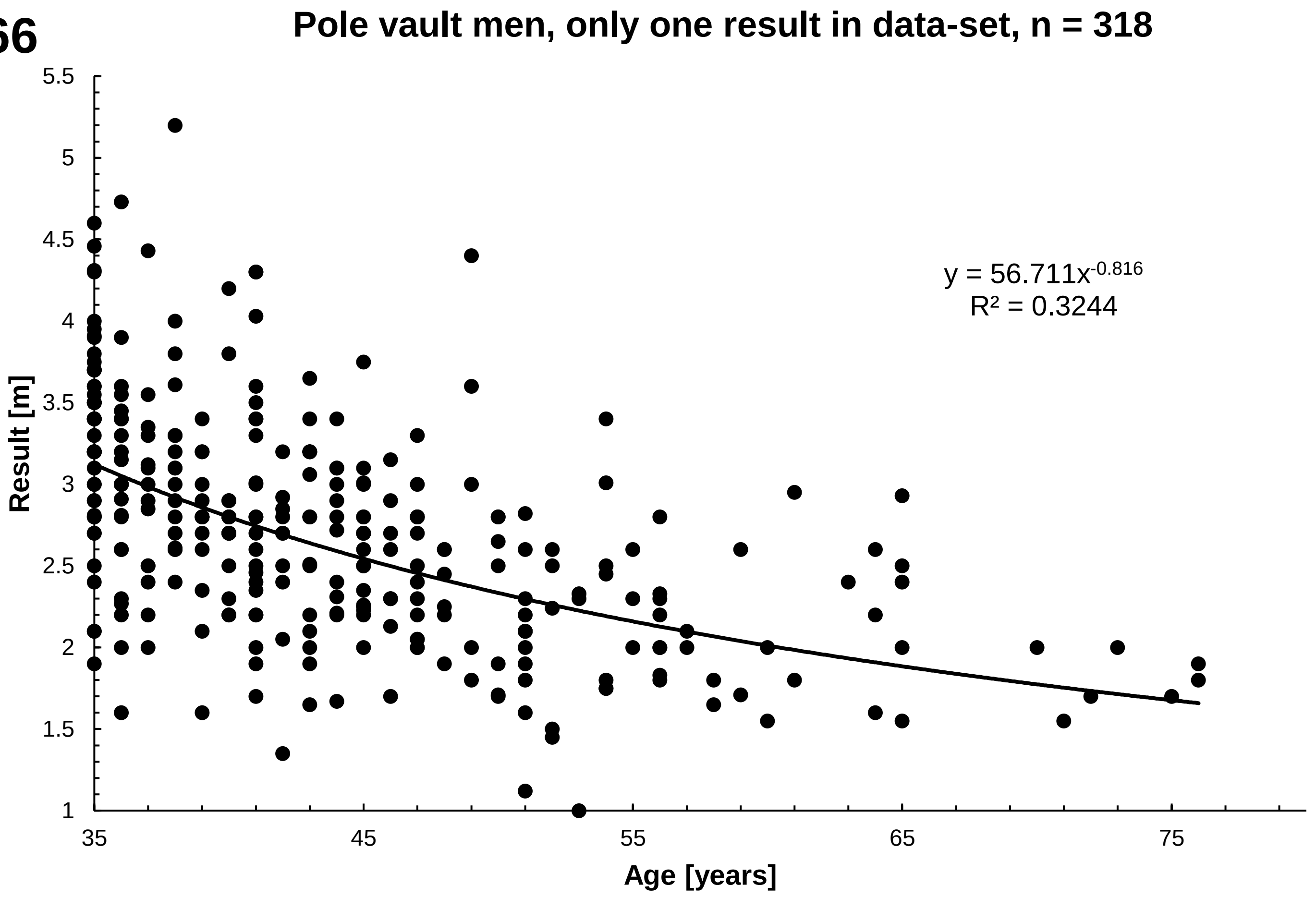
Pole vault men, only one result in data-set, n = 318
y = 56.711x^-0.816
R² = 0.3244
Result [m]
Age [years]
5.5
5
4.5
4
3.5
3
2.5
2
1.5
1
35
45
55
65
75

67

Discus men, 10 results and more, n = 198

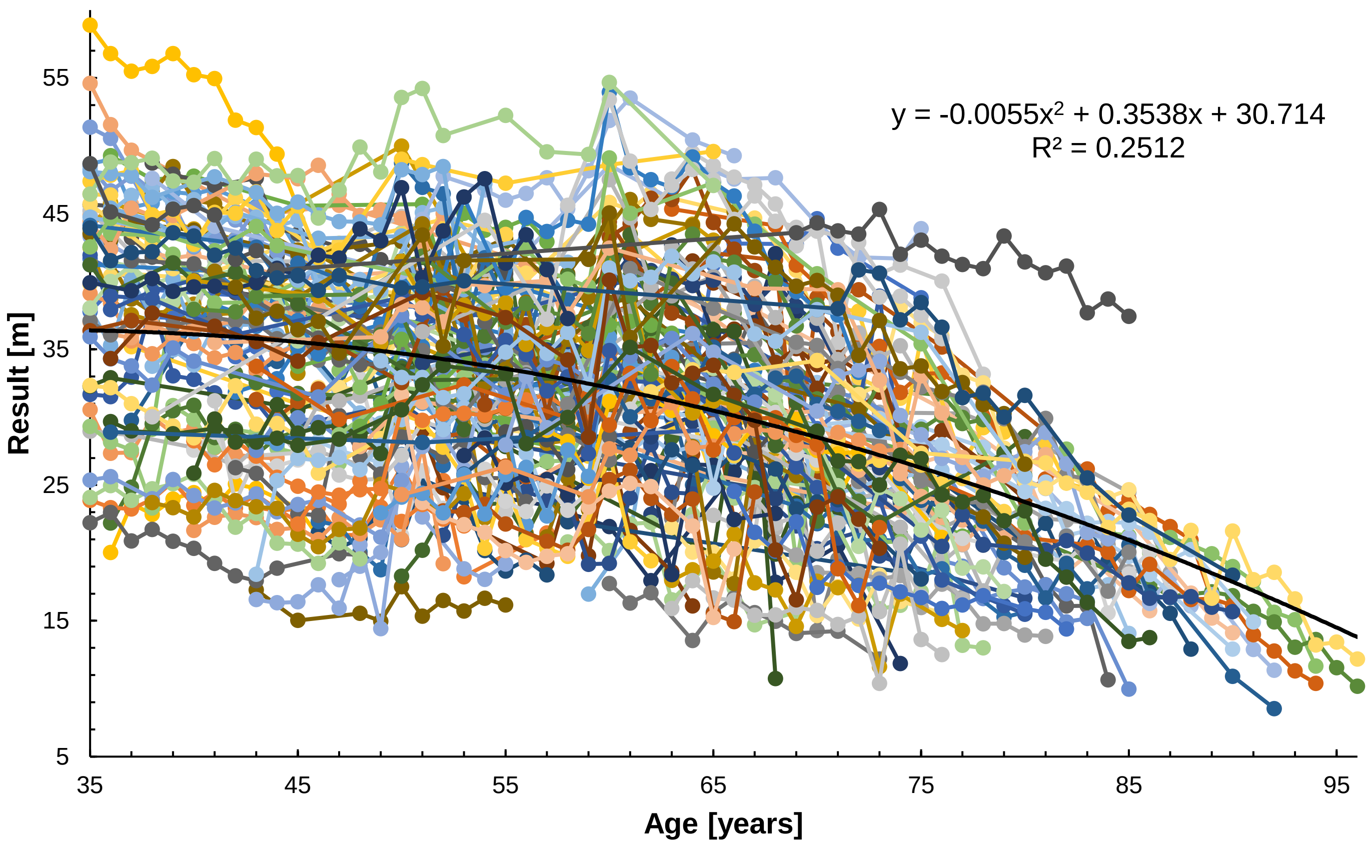

68

Discus men, 15 results and more, n = 49

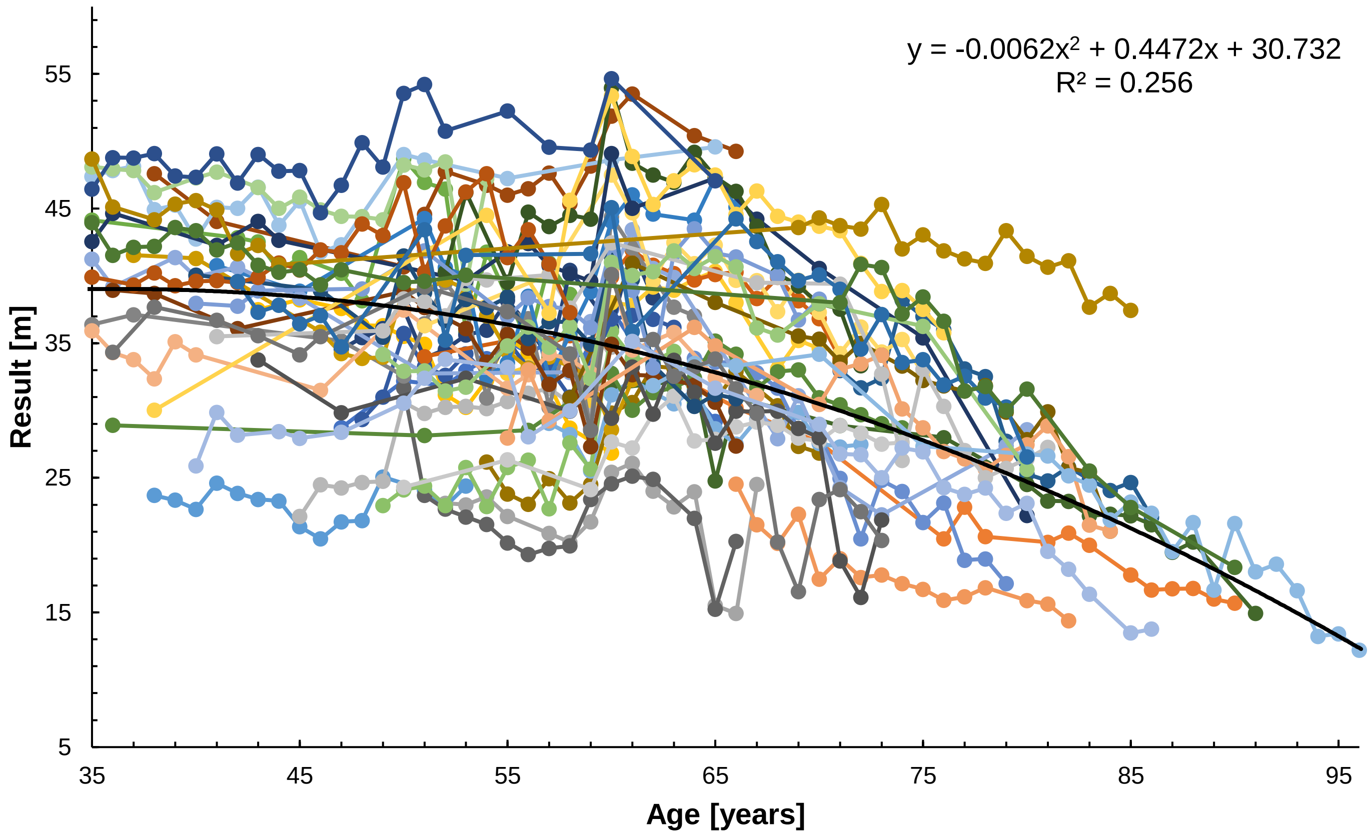

69 **Discus men, 20 results and more, n = 13**

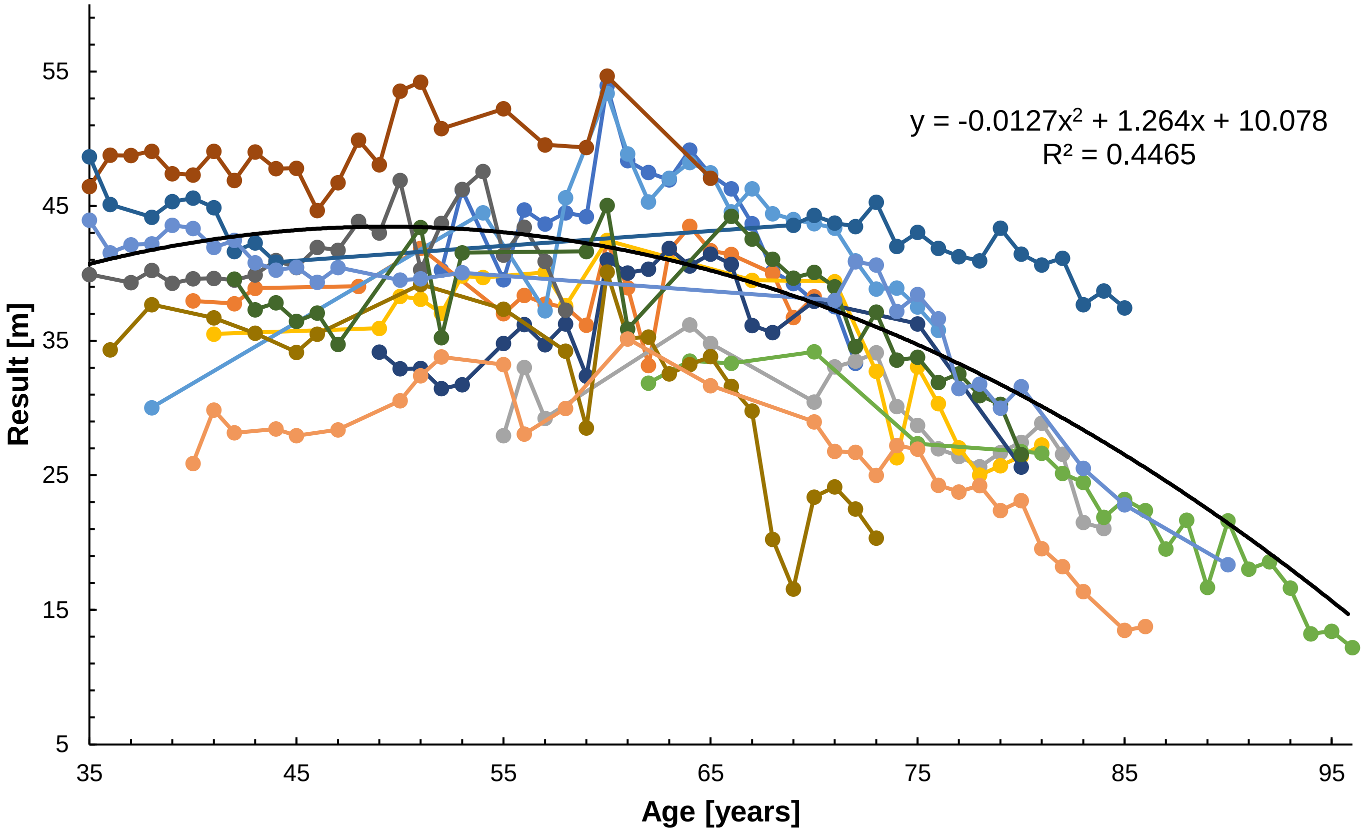

70 **Discus men, only one result in data-set, n = 1368**

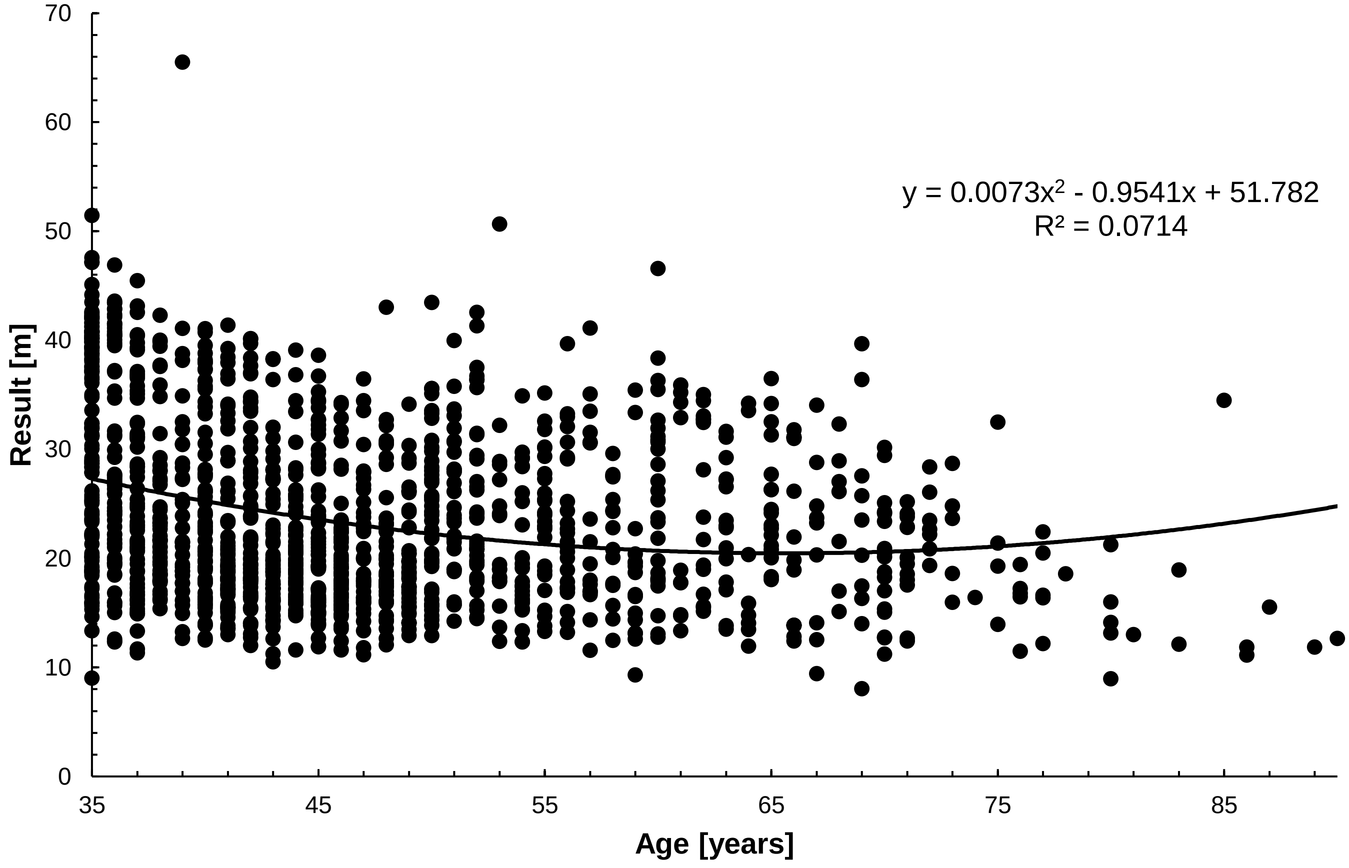

71

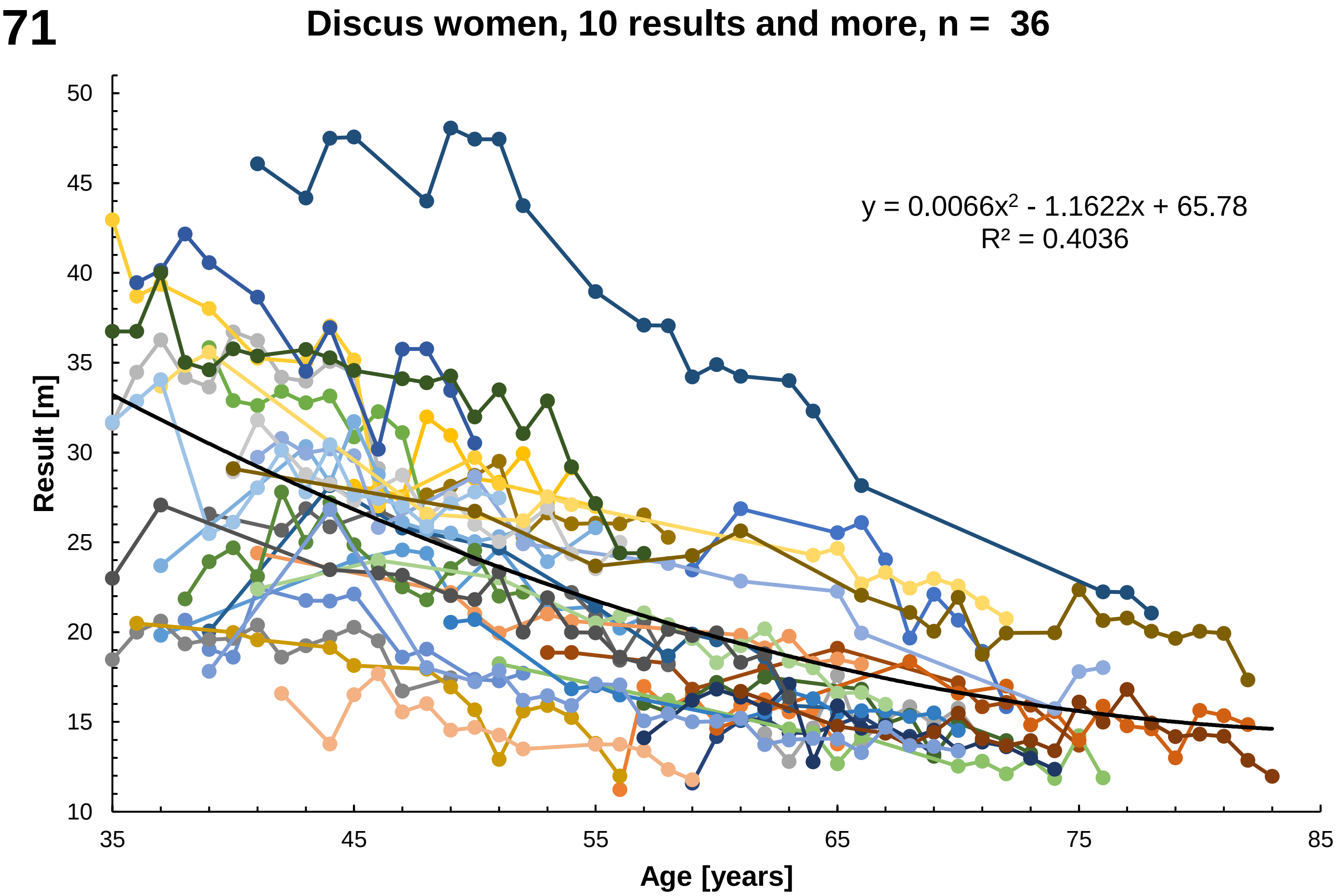
Discus women, 10 results and more, n = 36
y = 0.0066x2 - 1.1622x + 65.78
R² = 0.4036
Result [m]
Age [years]
10
15
20
25
30
35
40
45
50
35
45
55
65
75
85

72

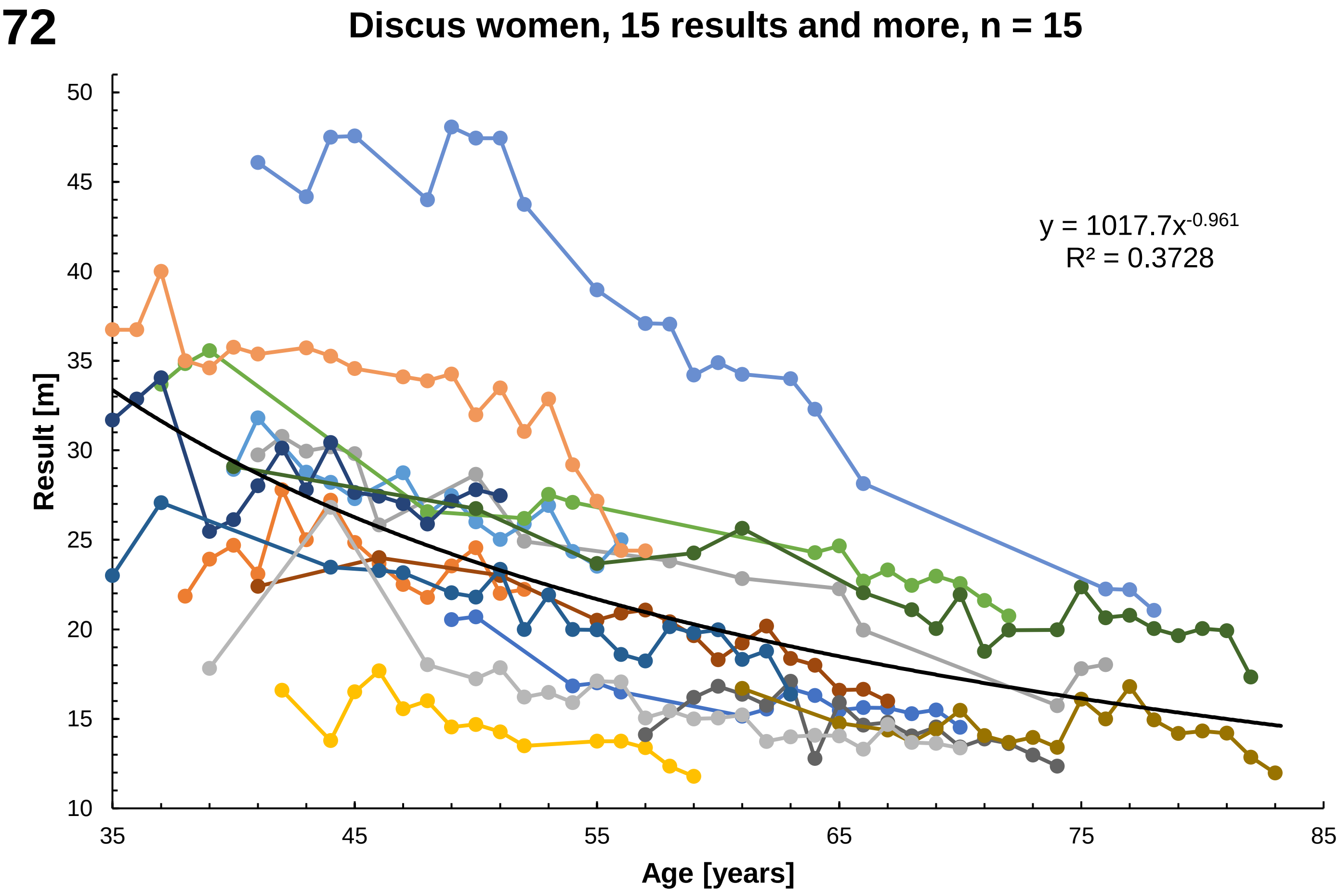
Discus women, 15 results and more, n = 15
y = 1017.7x-0.961
R² = 0.3728
Result [m]
Age [years]
10
15
20
25
30
35
40
45
50
35
45
55
65
75
85

73 Discus women, 20 results and more, n = 5

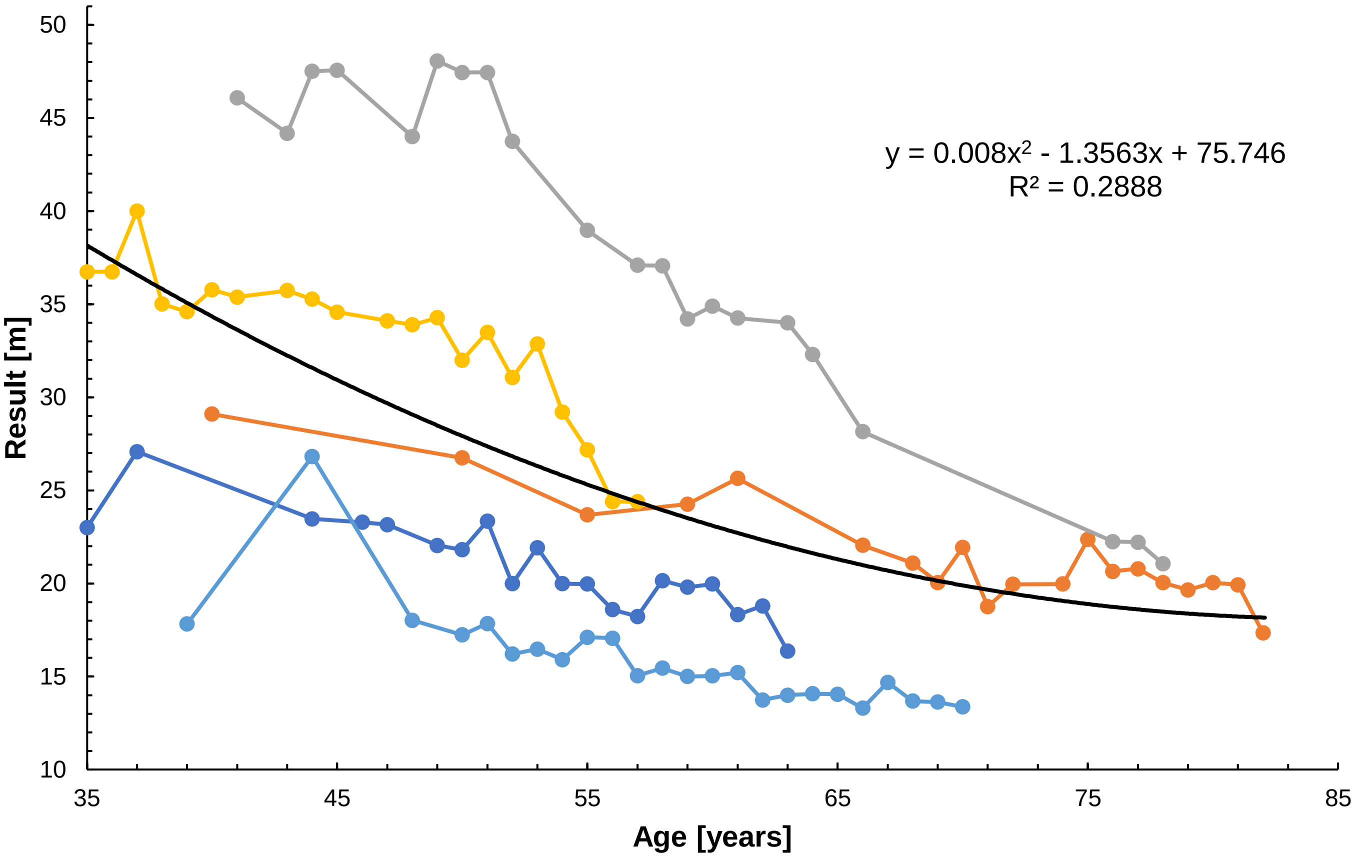

74 Discus women, only one result in data-set, n = 591

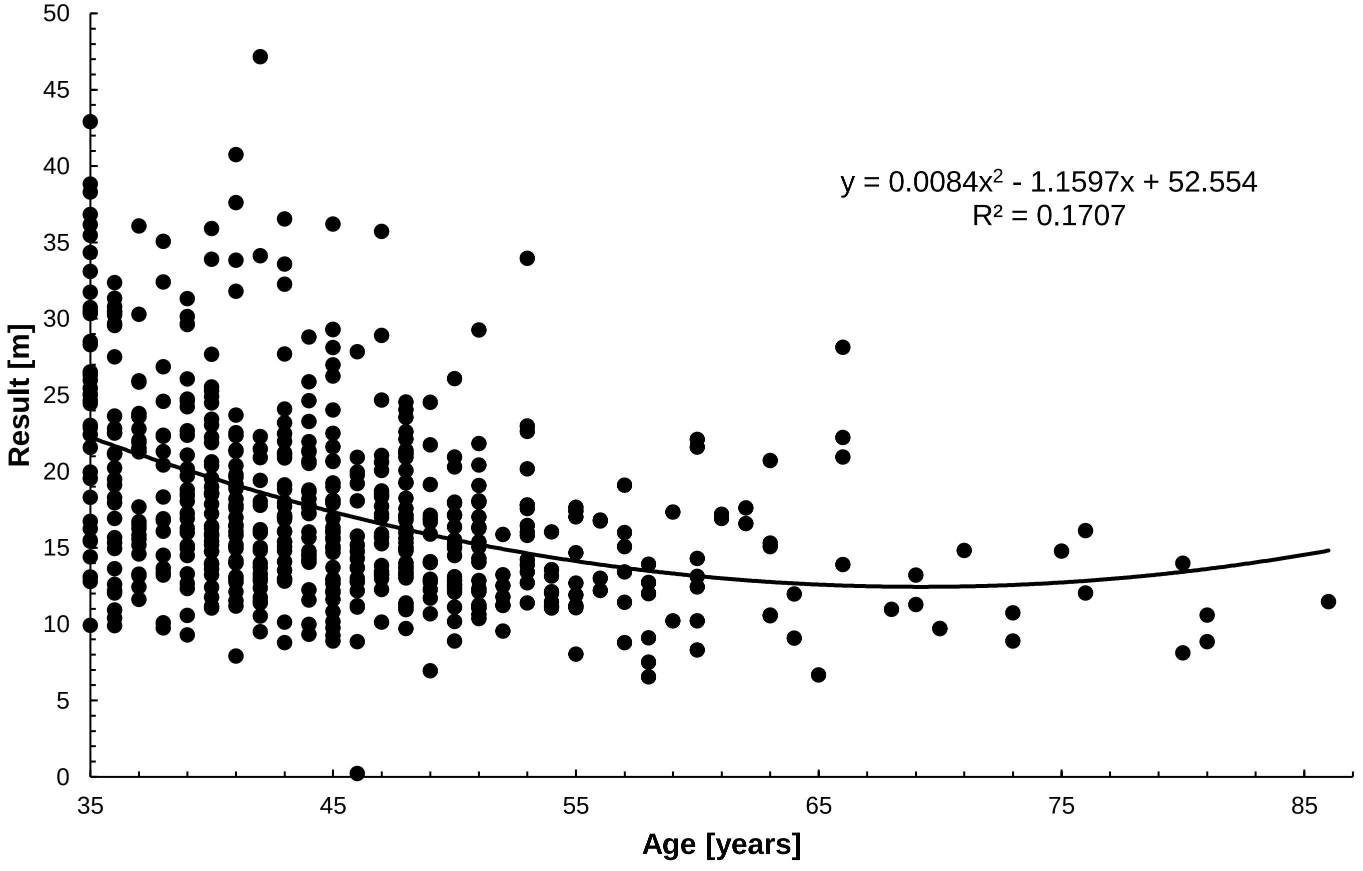

75

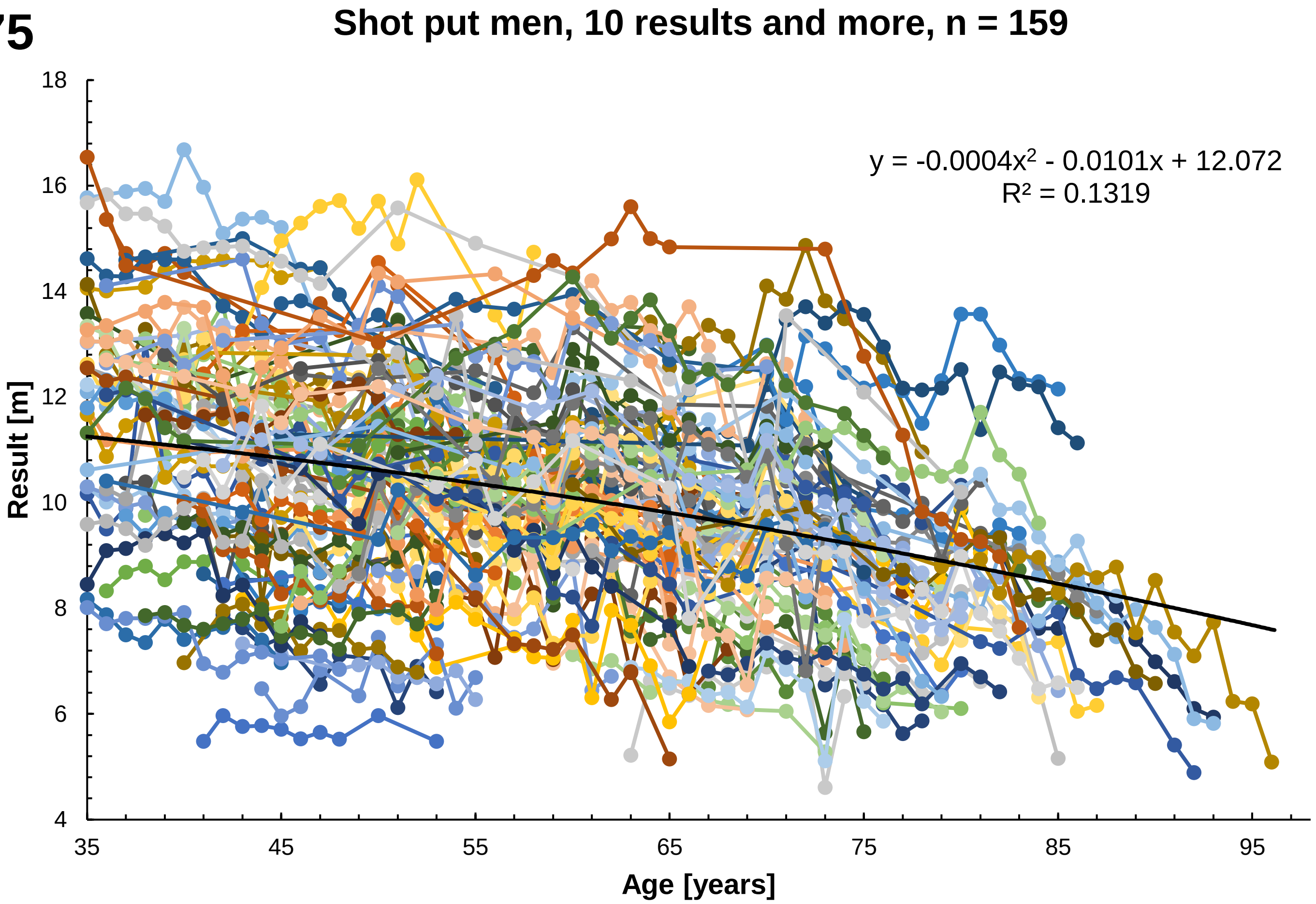
Shot put men, 10 results and more, n = 159
y = -0.0004x² - 0.0101x + 12.072
R² = 0.1319
Result [m]
Age [years]
18
16
14
12
10
8
6
4
35
45
55
65
75
85
95

76

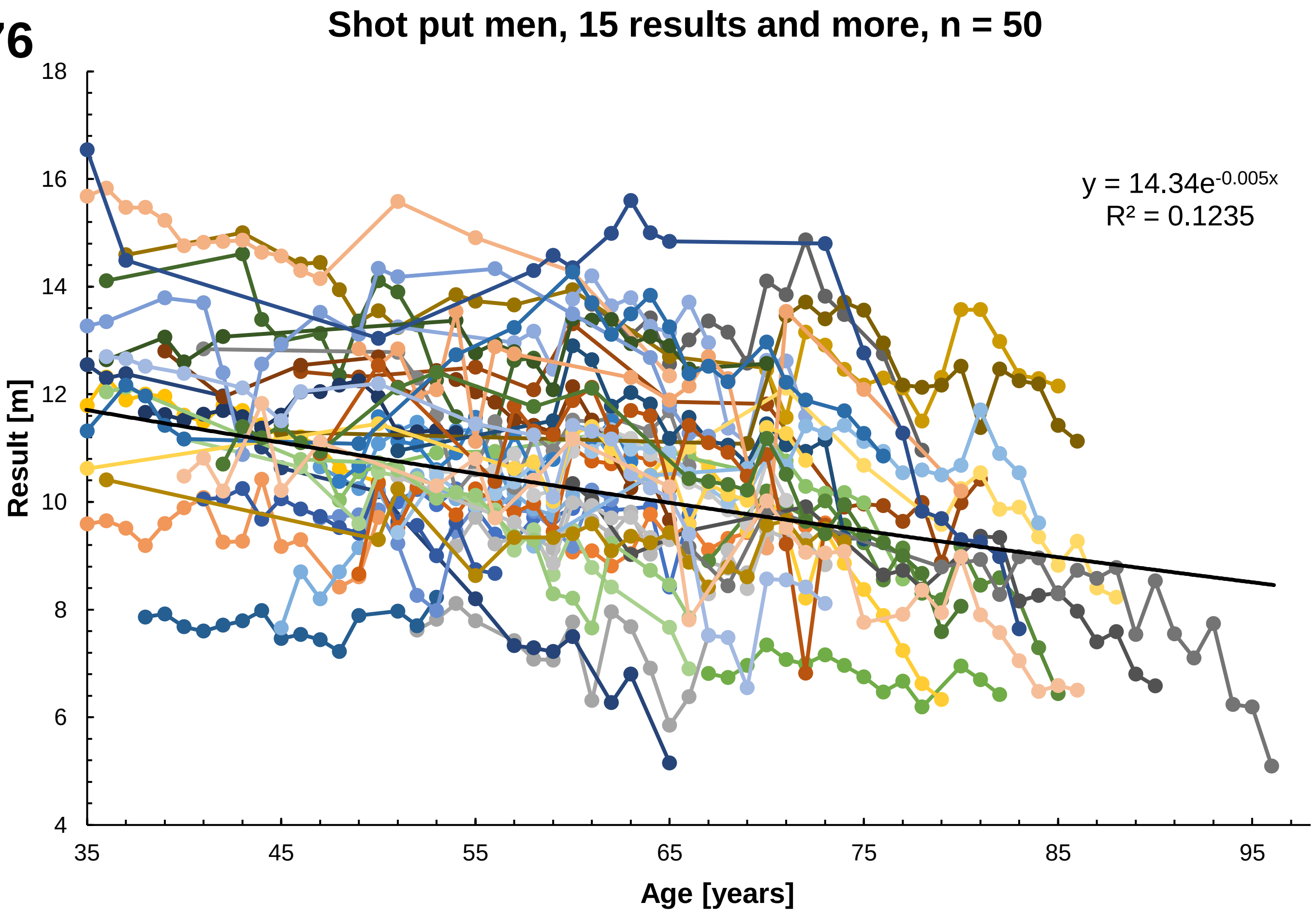
Shot put men, 15 results and more, n = 50
y = 14.34e-0.005x
R² = 0.1235
Result [m]
Age [years]
18
16
14
12
10
8
6
4
35
45
55
65
75
85
95

77

Shot put men, 20 results and more, n = 7

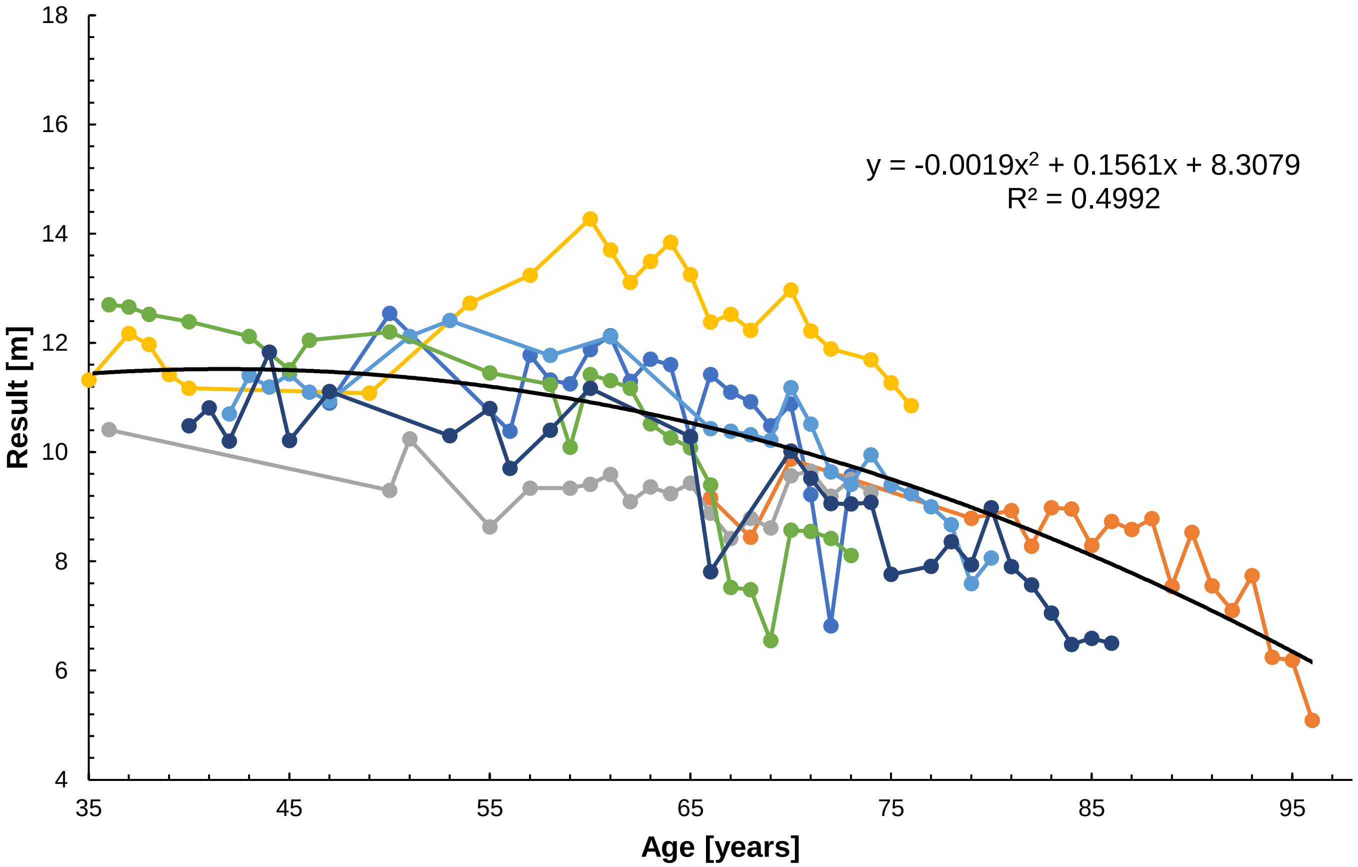

78

Shot put men, only one result in data-set, n = 1501

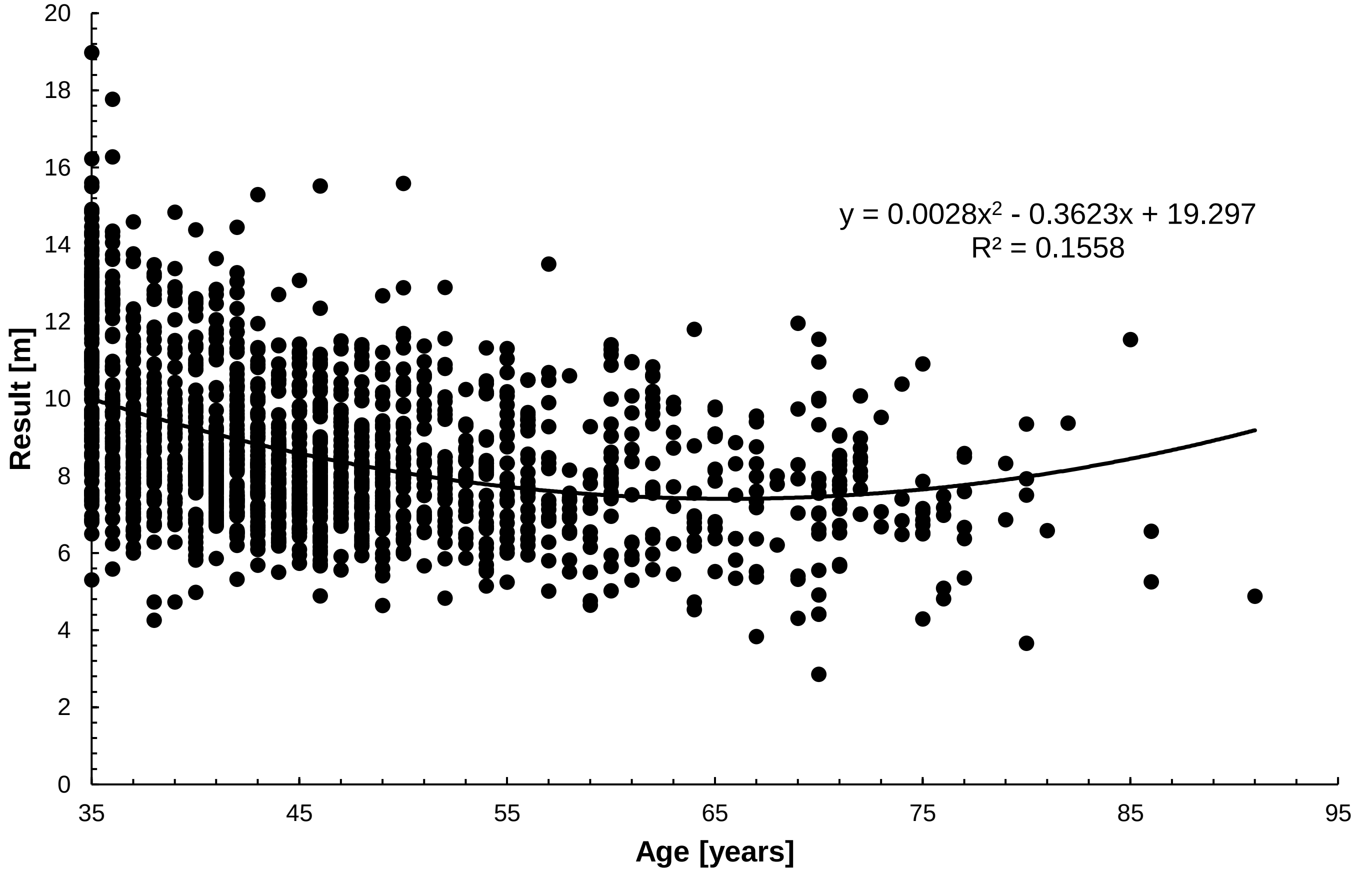

79

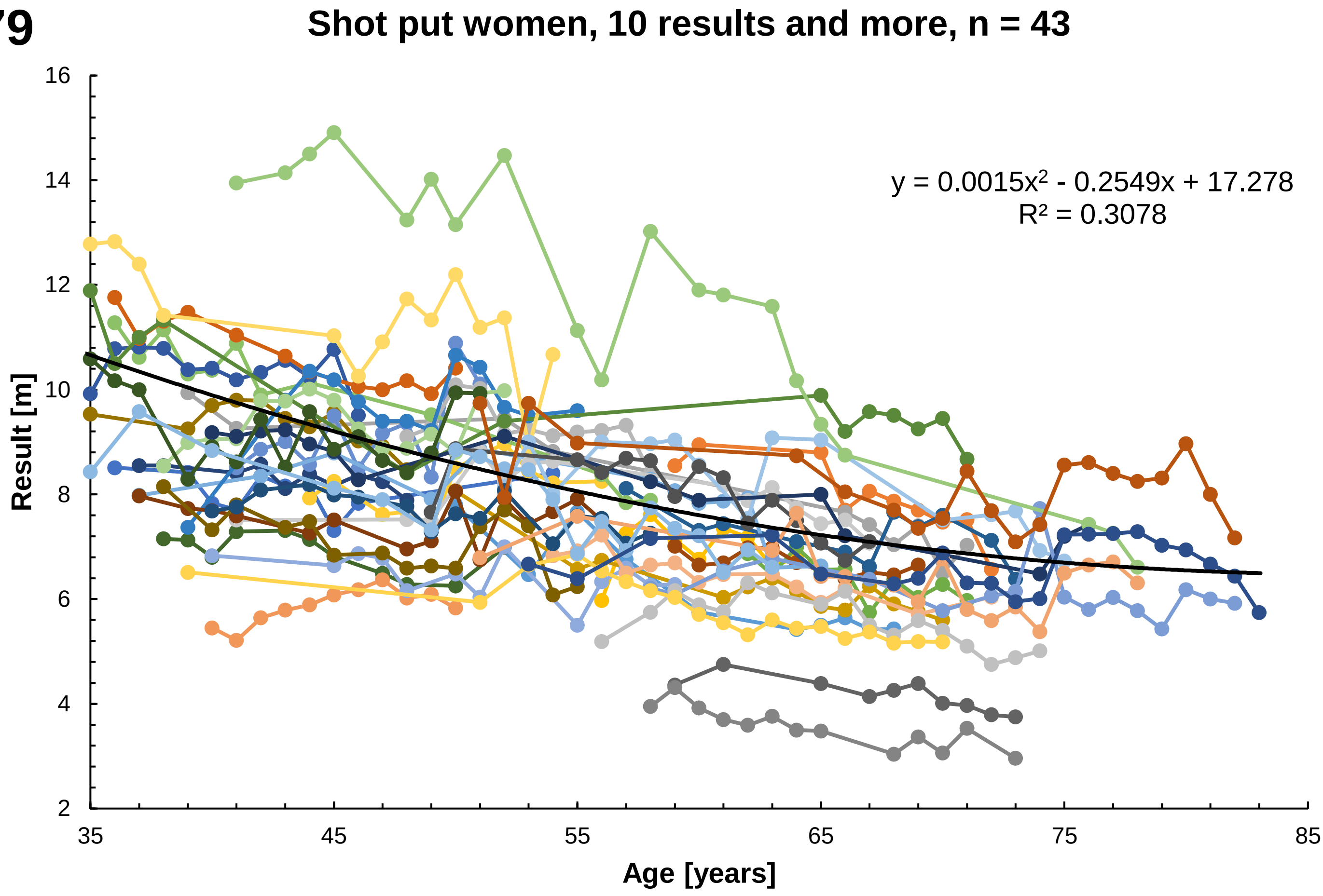
Shot put women, 10 results and more, n = 43
y = 0.0015x² - 0.2549x + 17.278
R² = 0.3078
Result [m]
Age [years]
16
14
12
10
8
6
4
2
35
45
55
65
75
85

80

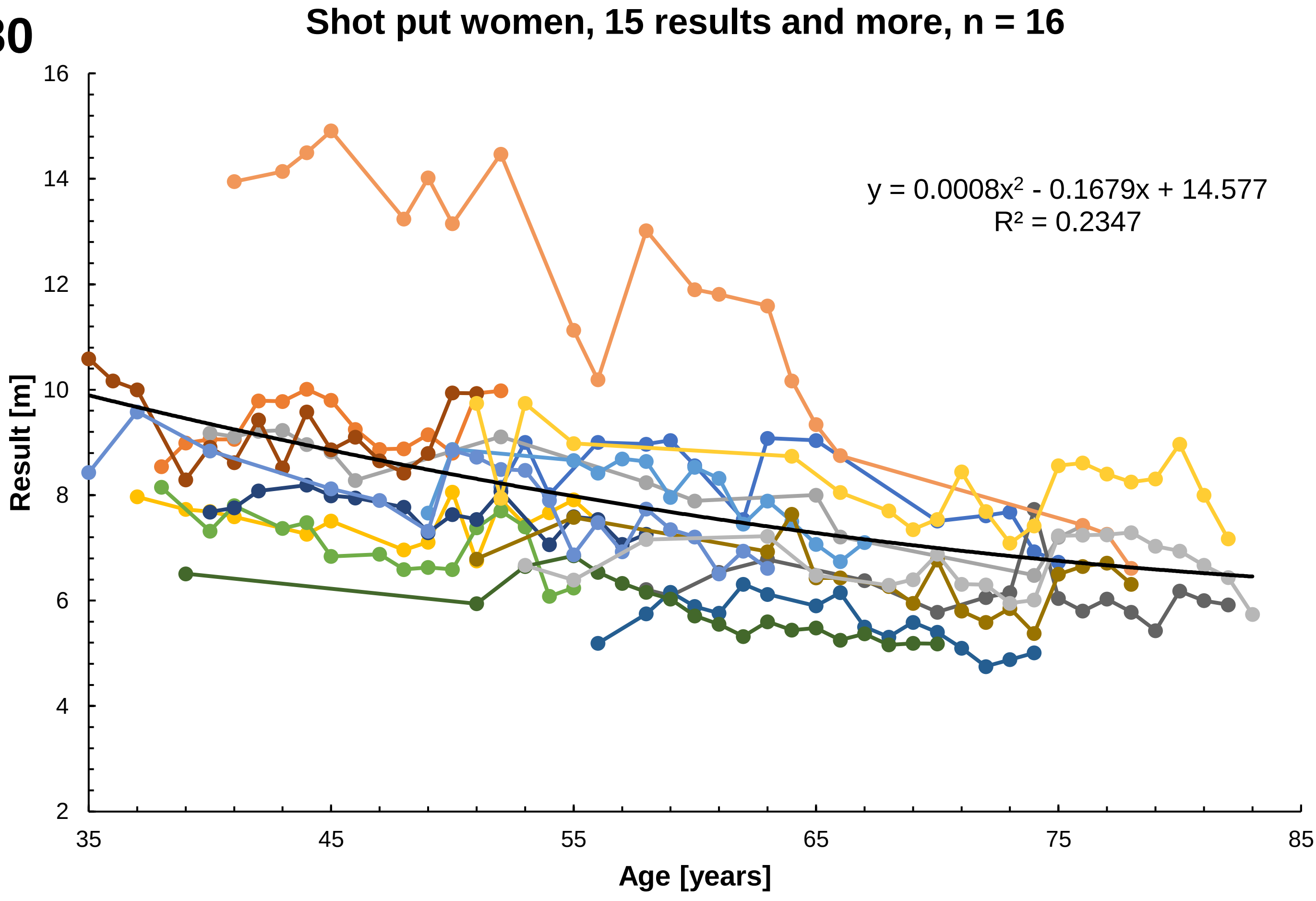
Shot put women, 15 results and more, n = 16
y = 0.0008x² - 0.1679x + 14.577
R² = 0.2347
Result [m]
Age [years]
16
14
12
10
8
6
4
2
35
45
55
65
75
85

81

Shot put women, 20 results and more, n = 4

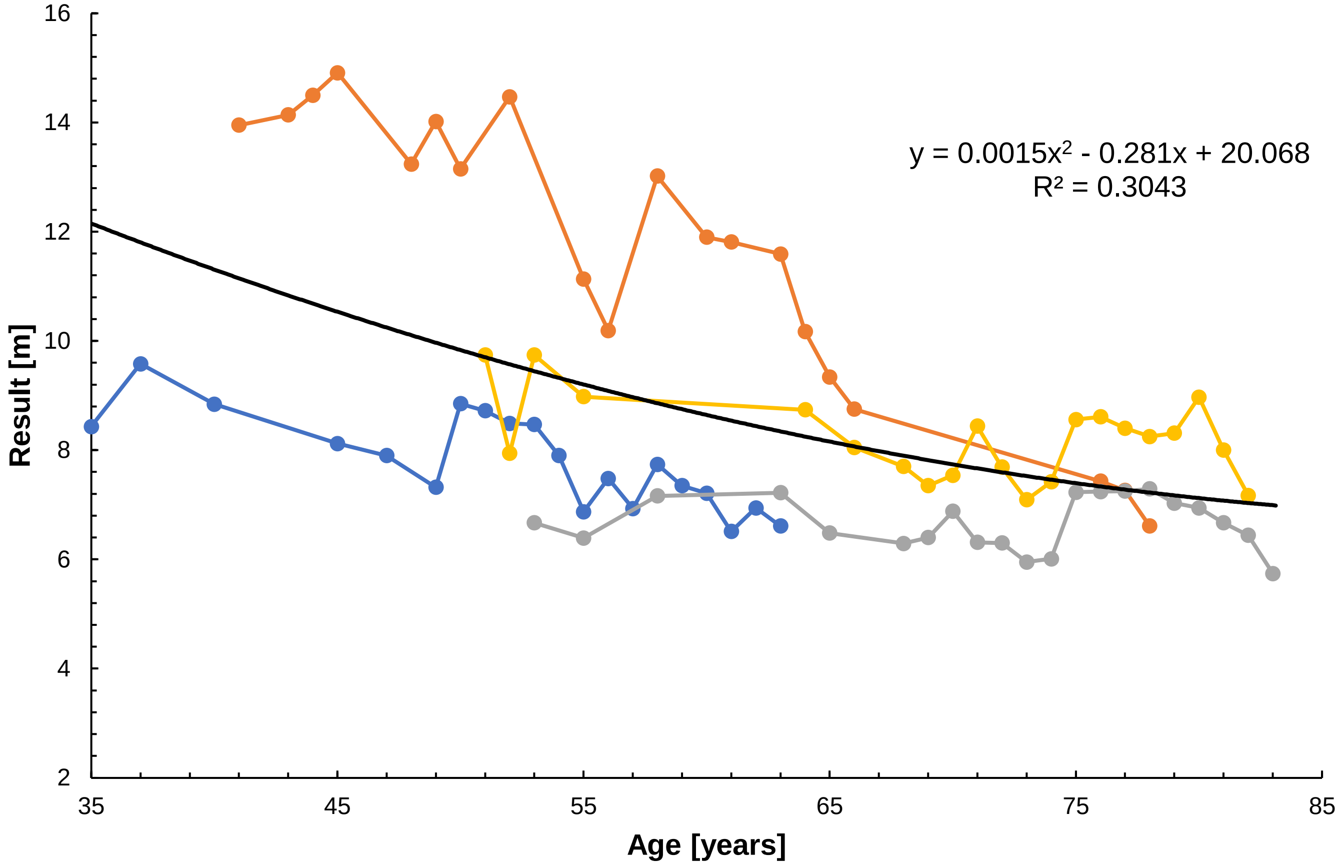

82

Shot put women, only one result in data-set, n = 778

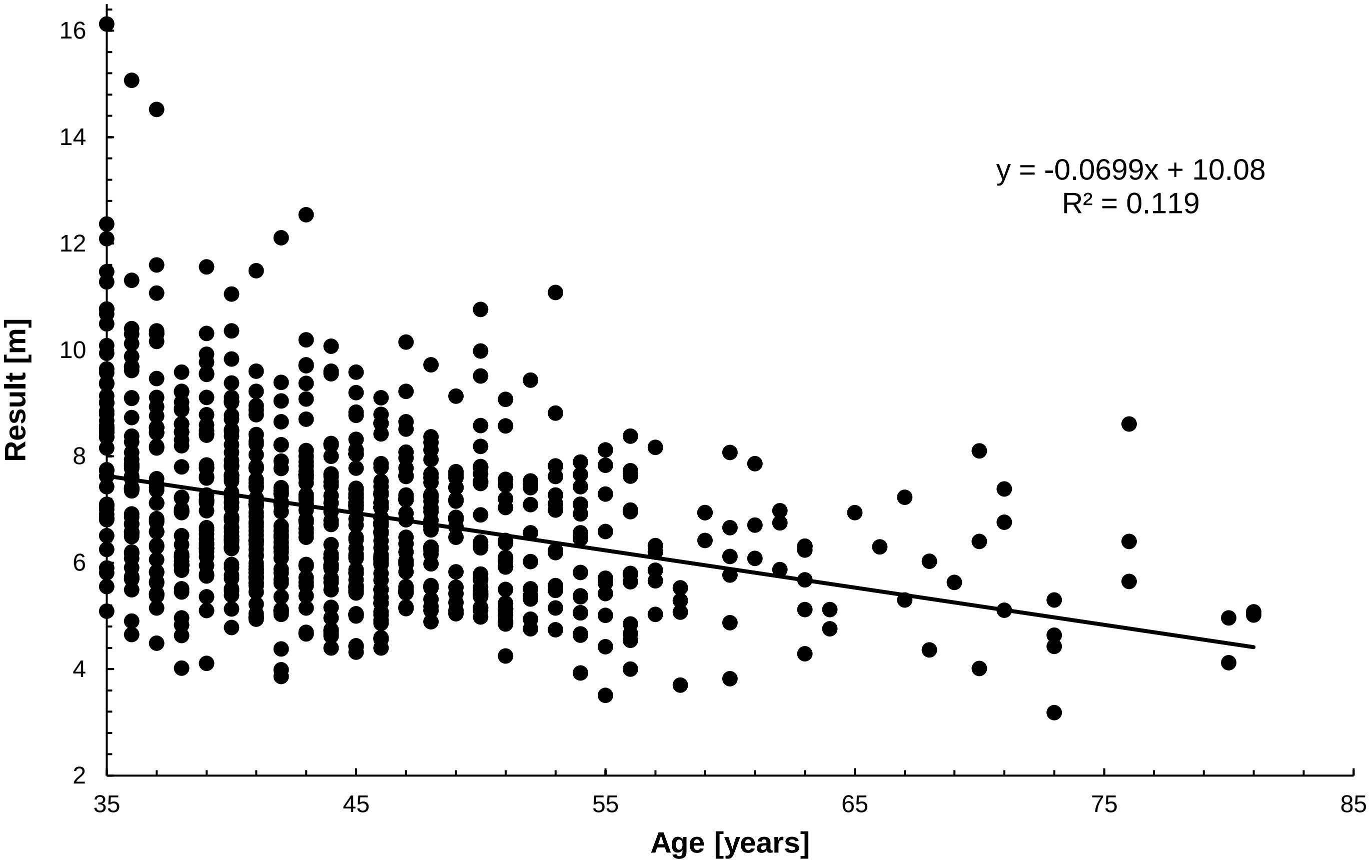

83

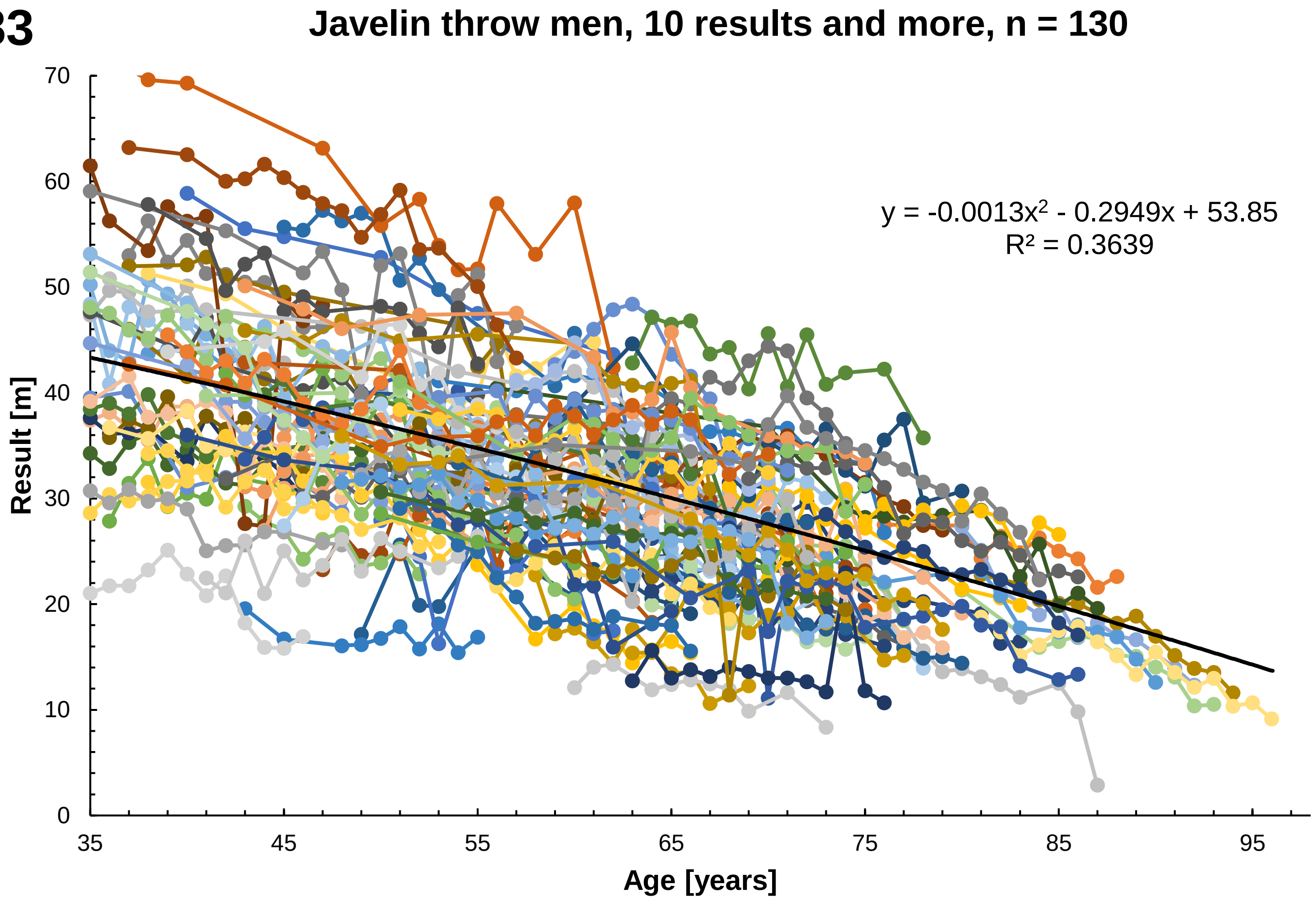
Javelin throw men, 10 results and more, n = 130
y = -0.0013x² - 0.2949x + 53.85
R² = 0.3639
Result [m]
Age [years]
0
10
20
30
40
50
60
70
35
45
55
65
75
85
95

84

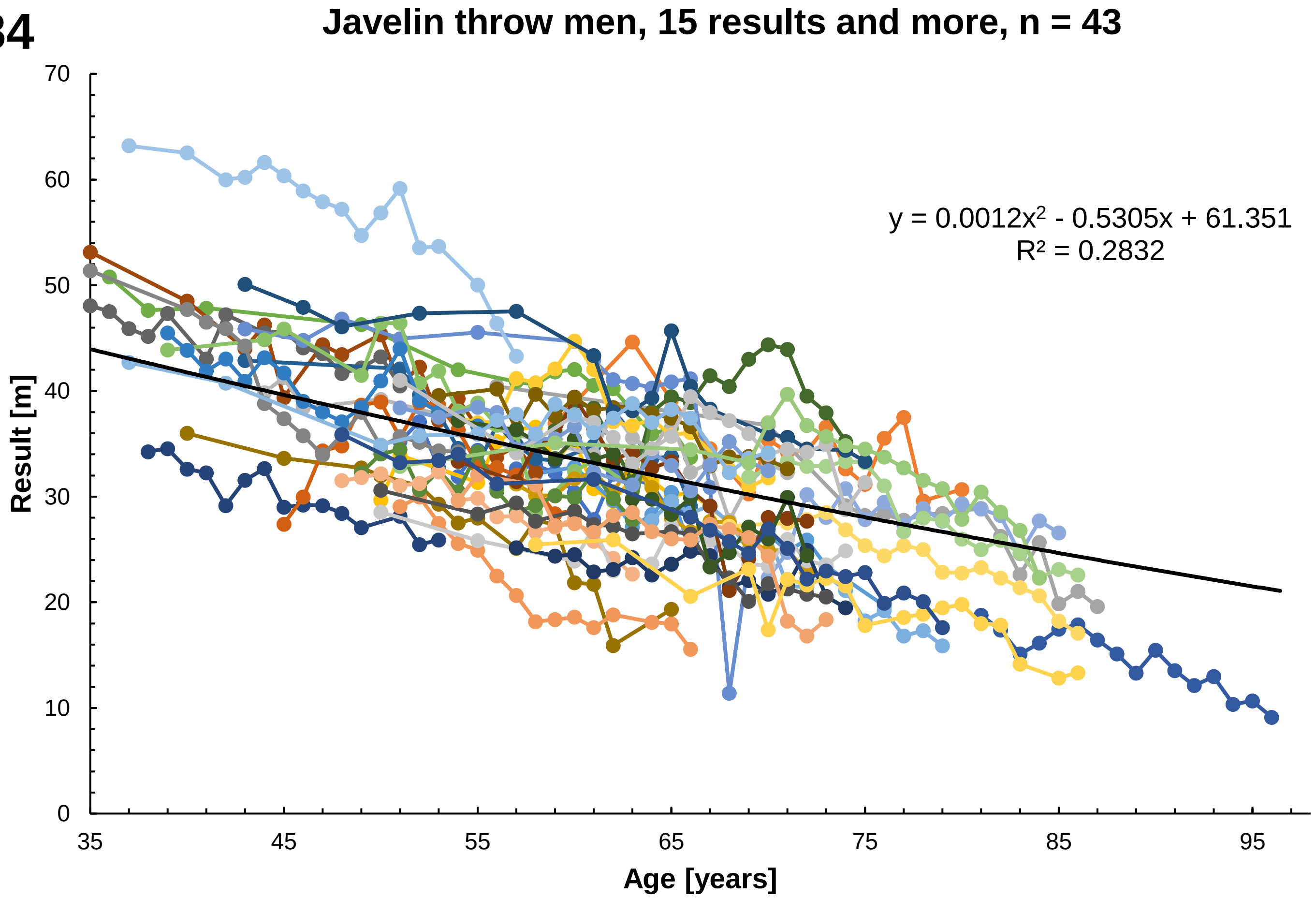
Javelin throw men, 15 results and more, n = 43
y = 0.0012x² - 0.5305x + 61.351
R² = 0.2832
Result [m]
Age [years]
0
10
20
30
40
50
60
70
35
45
55
65
75
85
95

85

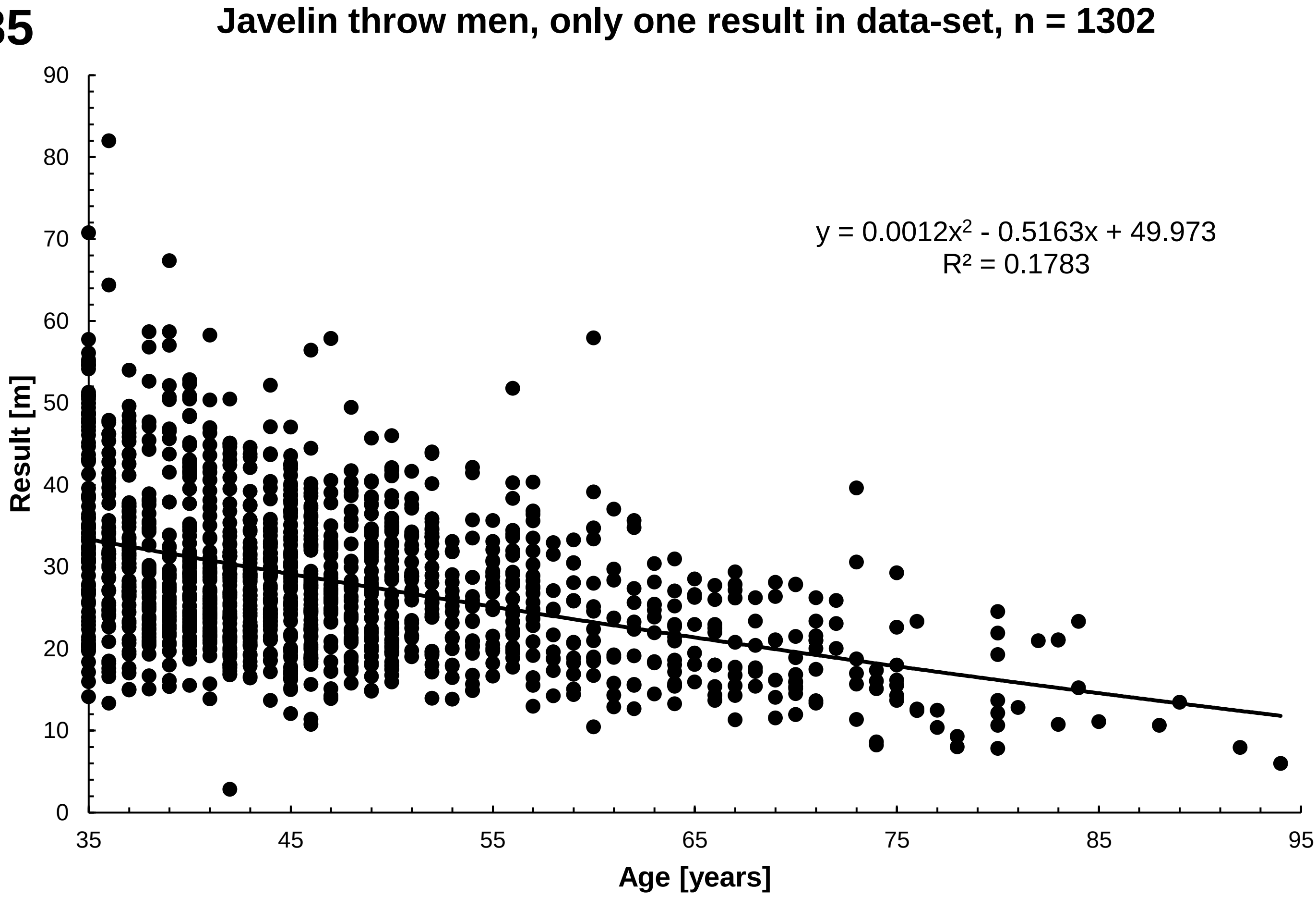
Javelin throw men, only one result in data-set, n = 1302
y = 0.0012x² - 0.5163x + 49.973
R² = 0.1783
Result [m]
Age [years]
0
10
20
30
40
50
60
70
80
90
35
45
55
65
75
85
95

86

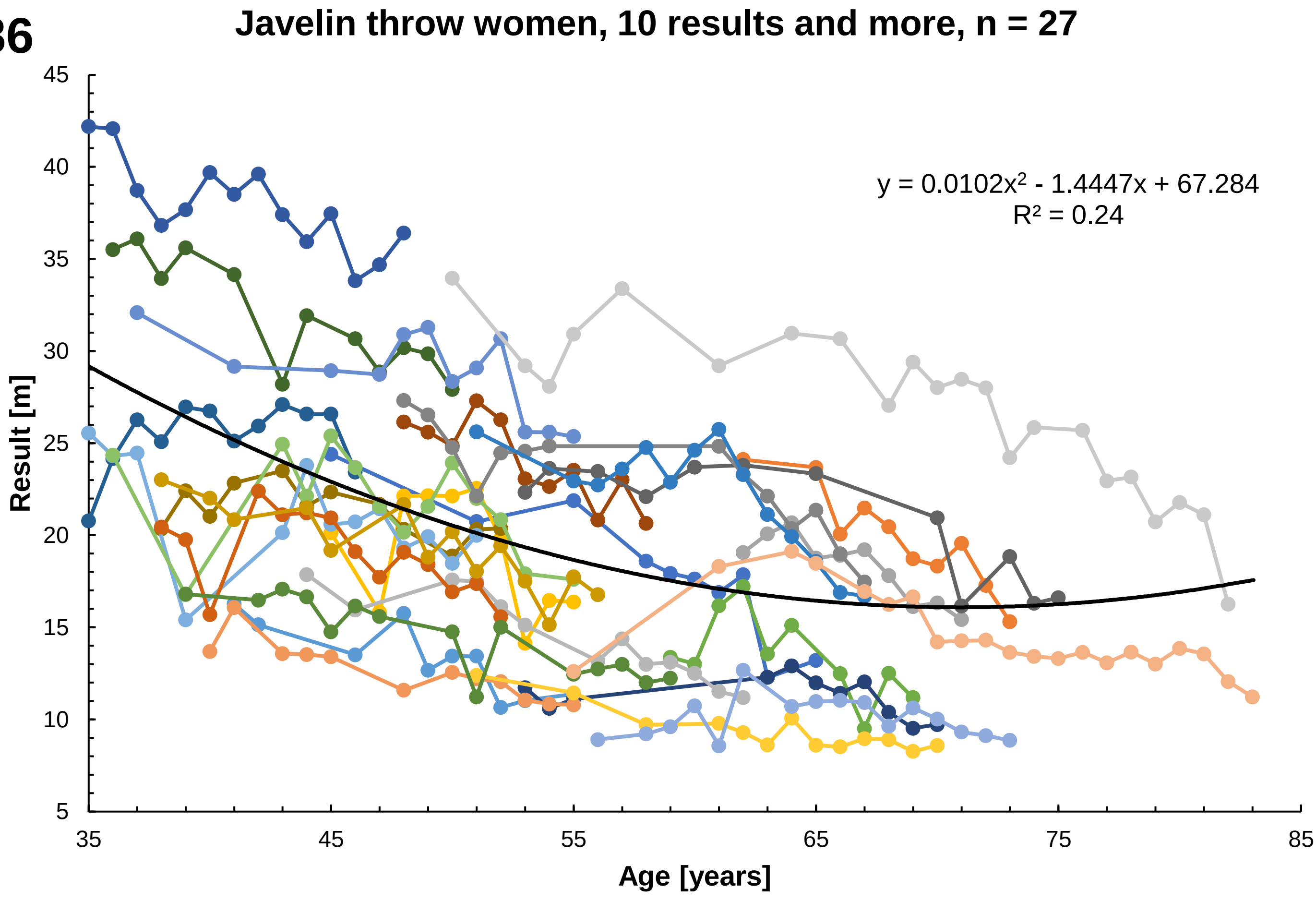
Javelin throw women, 10 results and more, n = 27
y = 0.0102x² - 1.4447x + 67.284
R² = 0.24
Result [m]
Age [years]
5
10
15
20
25
30
35
40
45
35
45
55
65
75
85

87 Javelin throw women, 15 results and more, n = 4

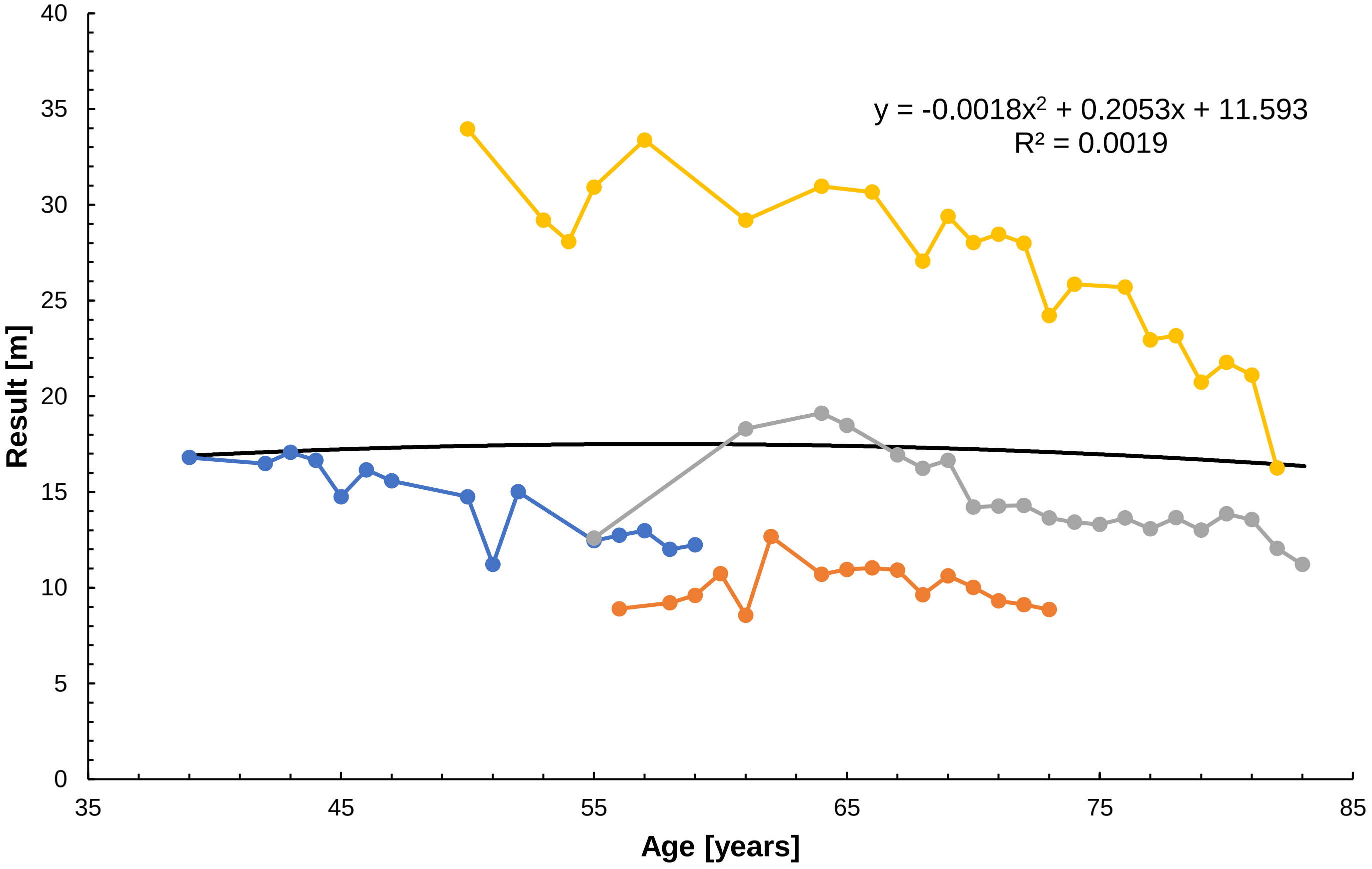

88 Javelin throw women, only one result in data-set, n = 519

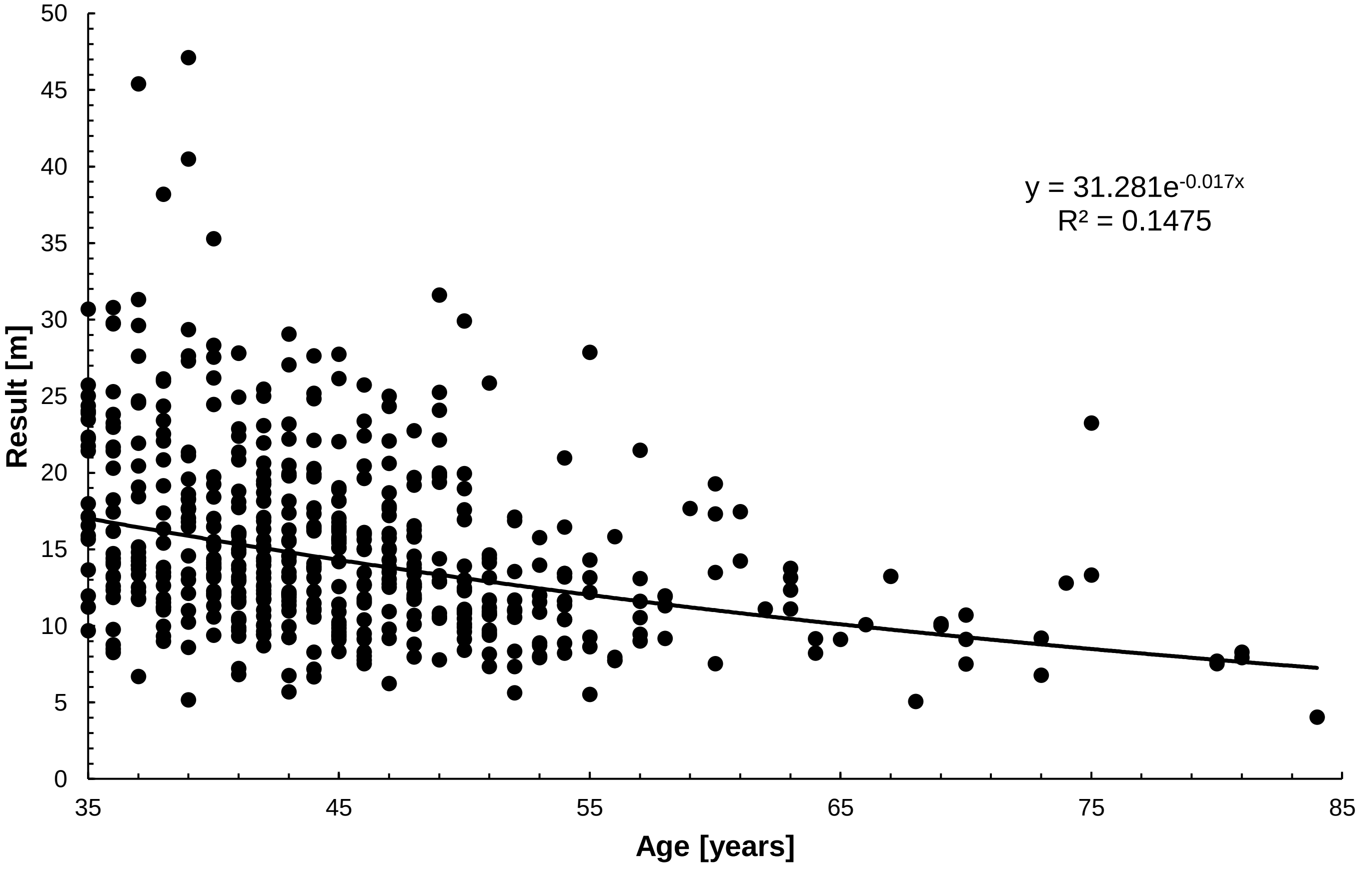

Supplement: Supplementary file 2 — CS and LN data shown for each of the 16 track and field events and both sexes. LN data is shown for athletes with 10, 15, 20 and 30 or more results in the data set, if 3 or more athletes are available. (PDF 3035 kb) [file 11357_2020_275_MOESM2_ESM.pdf]
