# Supplementary material for: Longitudinal trends in master track and field performance throughout the aging process: 83,209 results from Sweden in 16 athletics disciplines
Source: GeroScience. 2020 Oct 13;42(6):1609–20. doi: 10.1007/s11357-020-00275-0 (PMC7732911; doi:10.1007/s11357-020-00275-0)
Supplement: Supplementary file 3 — Supplementary Figs. 1 and 2 showing a comparison of the pooled regression lines of 10, 15, 20 and 30 results. (PDF 955 kb) [file 11357_2020_275_MOESM3_ESM.pdf]

Online Resource 3

GeroScience

Longitudinal trends in master track and field performance throughout the aging process: 83,209 results from Sweden in 16 athletics disciplines

Ganse B*, Kleerekoper A, Knobe M, Hildebrand F, Degens H

* Manchester Metropolitan University, b.ganse@mmu.ac.uk

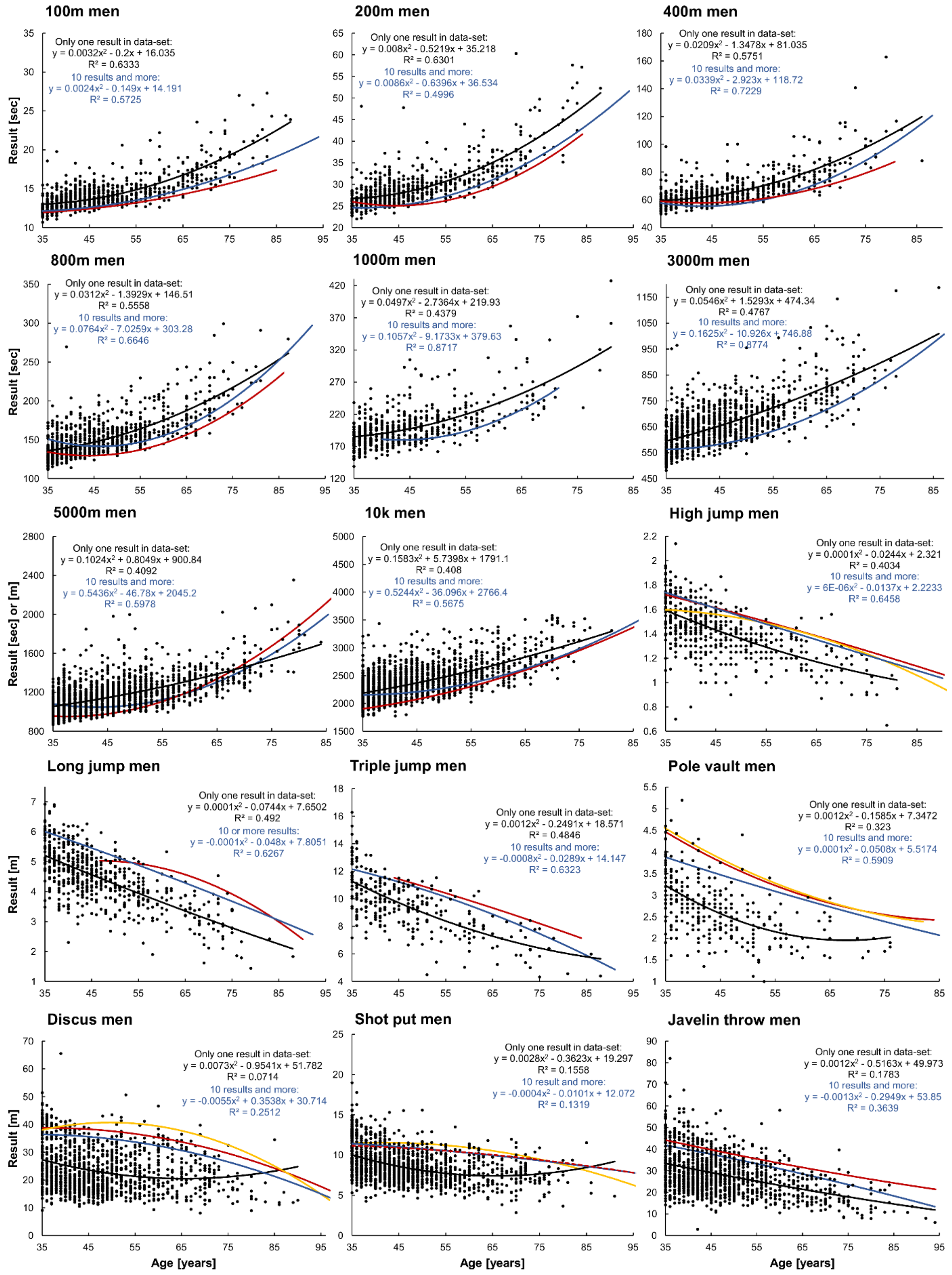

Supplementary figure 1: Results of male athletes with only one data-point in the data-set (for the named discipline) are shown as dots with a black trend line (= CS data). In addition, the regression lines of the LN data of male athletes who have 10 and more results in the data-set (blue), 15 and more (red) and 20 and more (yellow) are displayed, when at least three athletes exist. To allow comparison, all trend lines are shown as second-order polynomial models. See **Online Resource 2** for the regression equations resulting in the highest R^2-values for each group. It can be seen that the CS results have a worse average performance than the LN ones, while the LN regression lines are very close together.

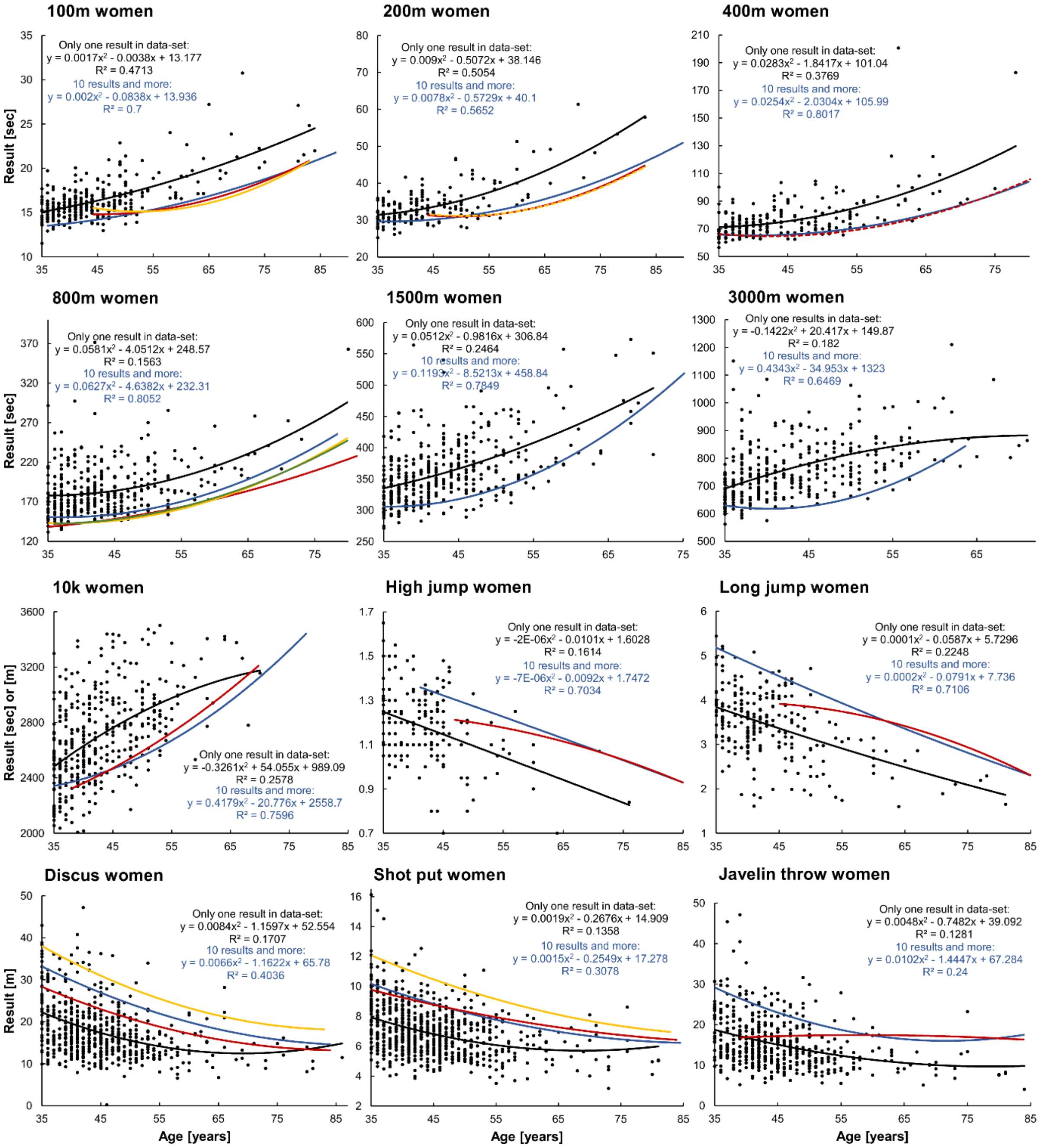

Supplementary figure 2: Results of female athletes with only one data-point in the data-set (for the named discipline) are shown as dots with a black trend line (= CS data). In addition, the regression lines of the LN data of female athletes who have 10 and more results in the data-set (blue), 15 and more (red), 20 and more (yellow) and 30 and more (green) are displayed, when at least three athletes exist. To allow comparison, all trend lines are shown as second-order polynomial models. See **Online Resource 2** for the regression equations resulting in the highest R^2-values for each group. It can be seen that the CS results have a worse average performance than the LN ones, while the LN regression lines are very close together.
